# Deployment of non-canonical splicing in tunicate genomes is mediated by divergent U2AF function and changing m6A modification in U1 and U6 snRNA

**DOI:** 10.1093/nar/gkag659

**Published:** 2026-07-02

**Authors:** Tze Chiew Christie Soo, Anthony Leon, Eugénie Waymel, Aubin Barais, Benjamin Porta, Daniel Chourrout, Simon Henriet

**Affiliations:** Michael Sars Centre, University of Bergen, Bergen NO-5020, Norway; Michael Sars Centre, University of Bergen, Bergen NO-5020, Norway; Michael Sars Centre, University of Bergen, Bergen NO-5020, Norway; Michael Sars Centre, University of Bergen, Bergen NO-5020, Norway; Michael Sars Centre, University of Bergen, Bergen NO-5020, Norway; Michael Sars Centre, University of Bergen, Bergen NO-5020, Norway; Michael Sars Centre, University of Bergen, Bergen NO-5020, Norway

## Abstract

Spliceosomal small nuclear RNA (snRNA) U1 and the U2AF heterodimer play critical functions by recognizing the highly conserved GT and AG dinucleotides, respectively, located at the start and at the end of introns. Here, we explore how changing these components contributed to maintaining splicing function in genomes where 95% of introns escape the GT/AG rule. By gaining access to new tunicate genomes, we could reveal that the emergence of non-canonical introns in the *Fritillaria borealis* lineage coincides with the duplication of U2AF subunits. Our findings indicate that paralogs U2AF1α and U2AF2α have preserved conserved functions, while divergent paralogs U2AF1β and U2AF2β form novel heterodimers that recognize introns with non-canonical 3′ ends. The conserved m6A present on U6 snRNA has been considerably reduced in *F. borealis*, but its U1 snRNA retains a stable 5′-terminal m6A, which is typically suppressed in humans and other chordates. We propose that this unique m6A pattern stabilizes the binding of snRNA to non-canonical 5′ splice sites. Although the core components of the spliceosome remain preserved, functional changes implemented through gene duplication and post-transcriptional modifications can significantly broaden the range of target splice sites.

## Graphical Abstract

**Graphical Abstract:**
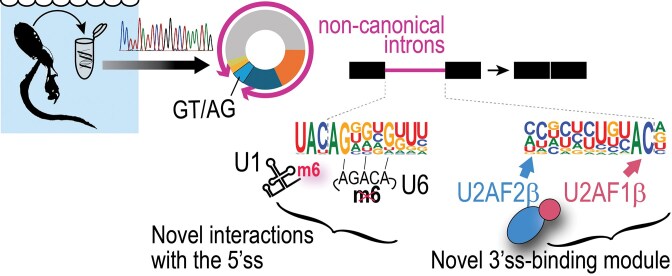

## Introduction

The spliceosome is a large complex composed of snRNPs, e.g. ribonucleoprotein particles (RNPs) assembled around a spliceosomal small nuclear RNA (snRNA), whose function is to remove introns from eukaryotic pre-mRNA. For most eukaryotic introns, the 5′ splice site (5′ss) is a GT and the 3′ splice site (3′ss) is an AG. Recognizing these terminal dinucleotides and their surrounding sequences is a critical prerequisite to spliceosome assembly on the intron, usually carried out by the U1 snRNP [1] and the U2AF complex [2]. The GT/AG rule prevails in nearly all eukaryotic introns, but it is largely unknown why these dinucleotides have been selected as dominant splice signals during evolution, whereas other termini represent typically <1% of introns. The essential function of splicing could be viewed as a considerable obstacle preventing changes at intron ends, however, some organisms challenge the GT/AG rule on a normal basis during development and on a significant scale in the genome, without compromising spliceosome function [3, 4].

**Figure 1.**
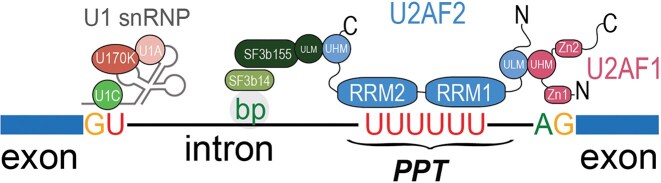
Recognition of splicing signals. The 5′ end of the intron is recognized by the U1 snRNP, the branchpoint nucleotide (bp) is recognized by the SF3b complex, and the U2AF complex recognizes the 3′ end of the intron. Each protein is indicated with a color code. In the case of U2AF1, U2AF2, and SF3b155, protein domains and interaction modules are represented.

The 5′ end of U1 snRNA is complementary to the eukaryotic 5′ss consensus, which includes the 5′GT and adjacent exonic and intronic nucleotides (Fig. 1). The 3′ end of introns is recognized by the U2AF heterodimer composed of two highly conserved proteins [2], a small subunit U2AF1 that contacts the 3′ss and a large subunit U2AF2 that binds to a polypyrimidine tract (PPT) found upstream [5, 6]. Stable association between pre-mRNA and these molecules is required for recruiting U2, U5, and U6 snRNPs that organize the catalytic site of the spliceosome. Minor introns are *stricto sensu* defined as recognized by U12-type spliceosome components U11 snRNA and ZRSR2 [7], which are the functional equivalents of U1 snRNA and U2AF in the U2-type spliceosome. Splice sites of minor introns include hallmark AT/AC termini, and they also include sequences differing only to a small extent from GT/AG introns consensus. However, splice sites differing substantially from the GT/AG consensus can be removed in organisms that possess only the U2-type spliceosome [8, 9]. Structural information gained on Group II introns and eukaryotic spliceosomes revealed that despite different splice site selectivity, their active sites are organized around a similar architecture [10–12]. The conserved stereochemistry could impose the limits of sequence evolution at intron ends, but it may also tolerate a larger variety of splice sites than what genomes usually show.

Our previous study reported an abundance of non-GT/AG introns in *Fritillaria borealis* [4], a larvacean tunicate found in the marine zooplankton. Although other deviations have been documented [3], the case of *F. borealis* remains exceptional for its amplitude, since <10% of introns adhere to the GT/AG rule. The U12-type spliceosome was lost in the larvacean ancestor [4, 13], and we could show that the U2-type spliceosome is necessary for removing all classes of introns in *F. borealis*. However, the mechanisms behind non-canonical splicing remained elusive at that time

Except for intron-poor organisms, components of the U2-type spliceosome have remained conserved during eukaryotic evolution [14, 15]. Presumably, a massive modification of splice sites would imply important changes in molecules that process introns but instead, we documented extensive conservation of the U2-type spliceosome in *F. borealis*—including a strict preservation of the 5′ end of U1 snRNA [4]. The 5′ss has been shown to interact with sequence motifs of other snRNA, such as the ACAGAGA box of U6 and Loop 1 of U5 [10]. These sequences, and the branchpoint recognition sequence present in U2 snRNA, are strictly conserved in *F. borealis*. Base substitution patterns in *F. borealis* snRNA also indicate selective pressure to maintain the consensus secondary structure of chordate snRNA [4]. It suggests that shifting spliceosome selectivity is rather mediated by discrete changes that emerged in the *F. borealis* lineage during evolution. Such changes may include the gain of novel splicing factors able to recognize RNA motifs associated with non-canonical introns. Chemical modifications present on conserved snRNA sequences or on the pre-mRNA could also contribute to the recognition of a larger variety of introns, by modulating interactions established between splice sites and snRNPs. Experiments described in this article allowed us to characterize the origins and the nature of these modifications, and to illuminate their role for recognizing non-canonical introns. By comparing the repertoire of conserved proteins that bind to splice sites in different genomes, we identified U2AF as one of the molecules in *F. borealis* that could contribute to identify non-GT/AG introns. We gained functional indications by documenting the functional divergence of *F. borealis* U2AF, and by showing that differences present on the modified bases of snRNA U1 and U6 could promote the flexible recognition of 5′ss in this species.

## Materials and methods

The detail of experimental procedures and statistical analyses, the list of reagents, and the list of databases used in this study are available in the “extended methods” section of the Supplementary data.

### Biological resources

Specimens of *F. borealis* originate from laboratory populations maintained at the Michael Sars Centre [16]. Specimens of *Fritillaria haplostoma* and *Appendicularia sicula* were collected near Bergen [4, 16]. Specimens of *Fritillaria pellucida* were collected near La Jolla (CA, USA) [4]. Cultures of HEK293T cells were established from frozen stocks [17] originally sourced from ATCC and were grown in complete Dulbecco’s modified Eagle’s medium (DMEM). A culture of HEK293T cells defective for AHCYL1 expression was prepared by transfecting Clustered Regularly Interspaced Short Palindromic Repeats / Cas9 (CRISPR/Cas9) AHCYL1 gene editing plasmid and AHCYL1 Homology Directed Repair plasmid (Santa Cruz) following the manufacturer’s instructions.

### Animal genome and transcriptome sequencing

We extracted high-molecular-weight genomic DNA from mature *F. borealis* gonads dissected under a microscope, with the Chomczynski procedure. The DNA of 190 *F. borealis* individuals was sequenced on MinION (ONT, Oxford Nanopore Technologies). We prepared Illumina libraries from the DNA of four mature *F. haplostoma* individuals, and barcoded libraries were sequenced together with *A. sicula* libraries prepared for an earlier study [4] at the Norwegian Sequencing Centre (NSC, Oslo, Norway). In parallel, we sequenced *A. sicula* DNA on PromethION (ONT) using material produced by whole-genome amplification from single individuals (WGA-DNA).

We extracted total RNA from *F. borealis, F. haplostoma*, and *A. sicula* specimens. Individual transcriptomes were processed following PacBio’s guidelines for isoform sequencing. SMRTbell libraries were sequenced on a Sequel II instrument at the NSC with 20 subread passes. Mean length of full-length non-concatemer reads was comprised between 1.4 and 1.6 kb and a poly-A tail was found 99% of the time.

### Genome assembly

We used long-read sequencing data to improve an existing assembly of the *F. borealis* genome [18]. The hybrid assembly was polished with Illumina reads from a single individual. For the *A. sicula* genome, we assembled ONT reads separately and corrected errors with Illumina reads produced from the same individual. For the *F. haplostoma* genome, we used paired-end Illumina reads for genome assembly, scaffolding, and gap-closing steps. Assembly statistics are described in Supplementary Table S1.

### Gene and intron annotations

For *F. borealis, F. haplostoma*, and *A. sicula* gene evidence and intron positions were obtained by aligning high-quality PacBio isoforms to genome assemblies. We first collected introns whose borders could be aligned without ambiguity. This allowed us to establish consensus rules (Supplementary Fig. S1) to precisely map additional introns with ambiguous borders. We used the same approach to annotate *F. haplostoma* and *A. sicula* introns. Introns of the *F. pellucida* genome were annotated based on gaps in BLASTX matches, using 30 987 proteins predicted from *F. borealis* as queries. We selected only cases for which amino-acid identity is strictly conserved in at least two-fifth codons in flanking exons. Gene orthologs were annotated with BLAST, using human protein sequences as queries. Top hits were checked for protein length and domain conservation, and annotation was confirmed by reciprocal BLAST against Uniprot.

### Expression and purification of U2AF heterodimers

Full-length open-reading frames (ORFs) were amplified from *F. borealis* complementary DNA (cDNA) and cloned in pRSFDUET-1 (see list of oligonucleotides in Supplementary Table S3). The U2AF2 ORF was fused with an N-terminal 6xHis tag (6His-U2AF2). The U2AF1 ORF was fused with an N-terminal tag that consists of the Twin-Strep-tag (TS-tag, TS-U2AF1), followed by amino acids 2–56 of the B1 domain of Streptococcal Protein G [19]. Protein expression was induced at 15°C with 50 µM IPTG (isopropyl β-D-1-thiogalactopyranoside), in the presence of 0.4% l-arabinose and 0.1 M ZnCl_2_. Cell pellets were lysed on ice in HHS buffer (Hepes 20 mM, NaCl 0.5 M, 10% glycerol, 1% Tween-20, pH 7.8) with lysozyme, 1 mM TCEP, and EDTA (ethylenediaminetetraacetic acid)-free protease inhibitors. Lysates were briefly sonicated and then incubated on ice with DNase, RNase, and 3% BioLock (IBA). Clarified lysates were loaded on a Strep-Tactin column. Bound proteins were successively washed with HHS, HLS buffer (Hepes 20 mM, NaCl 0.1 M, 5% glycerol, 0.5% Tween-20, pH 7.8), and HLS-5 mM biotin. After eluting proteins with HLS-0.1 M biotin, buffer was exchanged to HLS with 10 kDa MWCO membranes.

### Electrophoretic mobility shift assays

We cloned probe sequences between the T7 promoter and a SapI site. Templates cut with SapI were transcribed *in vitro* (IVT) and the reactions were DNase-treated before RNA dephosphorylation and purification on microcolumns (RNA-CC5, Zymo Research). We labeled 5′ ends with [γ^32^P]-ATP and purified the radioactive probes on PAGE (polyacrylamide gel electrophoresis)–urea. For binding assays, 100 cpm denatured probe was incubated for 1 h on ice in EMSA buffer [HLS, 50 ng/µl bovine serum albumin (BSA), 0.5 mM TCEP, 0.5 mM EDTA] with U2AF proteins purified from *Escherichia coli*. Our conditions provide at least five-fold molar excess of U2AF compared to the probe. RNPs were separated on 6% PAGE/TBE 0.5× at 4°C under 5 V/cm. Signals were recorded on fixed gels with phosphor screens.

### RNAcompete assays

We designed a pool of 5633 unique sequences corresponding to the 20 last nucleotides of *F. borealis* introns, followed by 10 nucleotides of the downstream exon. Sequences were inserted between the T7 promoter and SapI site, flanked by priming sites for polymerase chain reaction (PCR). Oligo constructs synthesized at Twist Biosciences were PCR-amplified before IVT and dephosphorylation as described above.

We cloned U2AF1 ORFs in pGEX-6P-1. Protein expression was induced at 18°C with 0.4 mM IPTG. Cell lysis and nucleic acid removal were performed as described above in LYB buffer (25 mM Hepes, 0.35 M NaCl, 10% glycerol, 0.5% Tween-20, pH 7.5). Clarified lysates were incubated with Glutathione (GSH)-sepharose 4B slurry at room temperature. Bound proteins were washed with LYB, eluted with LYB-50 mM GSH, and exchanged in LYB as described above.

For binding assays, 2 pmol GST-U2AF1 were bound to GSH magnetic agarose beads in 0.2 ml RBB buffer (25 mM Hepes, 0.5 M NaCl, 5% glycerol, 1 mM EDTA, 1 mM dithiothreitol (DTT), 2 mM MgCl_2_, 0.5% Tween-20). Beads were washed with RBB washes, and we added 1 µM of RNA pool in 0.5 ml RBB with 10 µg BSA and 4 µg Heparin. Beads were incubated at 4°C and washed with RBB. Proteins were digested with Proteinase K–sodium dodecyl sulfate (SDS), and we recovered RNA with RNA-CC5. We prepared small RNA sequencing libraries and sequenced samples at the NSC. Reads were mapped on the original intron pool with Bowtie2.

### Mammalian cell expression, transfection, and GST-pulldown

We cloned U2AF2 ORFs in pCDNA3.1, in C-terminal fusion with a V5/6xHis tag. U2AF1 ORFs were cloned in pSF-CMV, in N-terminal fusion either with GST or with the TS-tag and solubilization domain described above. Control Env constructs have been described previously [17]. Plasmids were transfected with Lipofectamine to HEK293T cells grown in DMEM, and cultures were harvested 48 h post-transfection. GST-pulldowns were performed as described by Tari *et al.* [20].

### Long-read sequencing of mammalian cDNA amplicons

Cells transfected with either combinations of pCDNA-U2AF2.6His/V5 and pSF-CMV-TS-U2AF1 or a pCDNA-LacZ control were treated with 0.15 M cycloheximide for 8 h prior harvest, or with 0.1% dimethyl sulfoxide (DMSO) for controls. After RNA extraction and DNAse treatment, 1 µg RNA was reverse-transcribed, with random 10-mers and oligo-dT priming. Each target cDNA was amplified separately with 20 PCR cycles. We pooled the cDNA from the same transfection to prepare barcoded libraries for sequencing amplicons (SQK-LSK114, ONT). We sequenced the library pool on a PromethION and selected Q9 reads for basecalling. We processed the reads for isoform classification with TALON, using a database corresponding to regions targeted by the gene-specific primers.

### Transcriptome analysis of U2AF2-expressing cells

We enriched mRNA from Trizol-extracted RNA with the Dynabeads mRNA Purification Kit (Ambion). Barcoded Illumina sequencing libraries were prepared from poly-A^+^ RNA and sequenced at the NSC. The data were mapped with STAR to *Homo sapiens* GRCh38.p14 primary genome assembly using the latest GENCODE annotation. We determined alternative splicing (AS) events using the rMATS turbo software.

### Crosslinking and immunoprecipitation followed by sequencing

Cells transfected with either pCDNA-U2AF2.6His/V5 or pCDNA-Env.6His/V5 were irradiated on ice with 254-nm bulb (150 mJ). After cell lysis, transcripts were fragmented for 3 min at 37°C with 20–1000 U/ml RNaseI. Clarified lysates were added to Anti-V5 magnetic beads and after binding at 4°C, RNP wash, RNA isolation, and preparation for sequencing were conducted as described [21]. Libraries were sequenced at the NSC. Reads were processed as described by Busch *et al.* [22], and we used Pureclip [23] to call U2AF2-V5 crosslink peaks, using Env-V5 crosslinks as an input control. Sequence analysis was conducted on unique peaks.

### Identification of U2AF2 partners

Cells transfected with V5-tagged proteins were lysed in HLS supplemented with 1 mM TCEP and EDTA-free protease inhibitors. We used 6 µg protein for immunoprecipitation (IP). Samples were pre-cleared with Anti-HA magnetic beads at 4°C, and the supernatant was bound to Anti-V5 magnetic beads at 4°C. After washes with HLS, proteins were eluted in Laemmli buffer and separated on Bis–Tris SDS–8% PAGE. Peptides were digested in gel and analyzed on an Orbitrap Eclipse Mass Spectrometer at the Proteomics Unit of the University of Bergen. After peptide identification, we established a database that include all proteins identified in the samples, and without common contaminants such as keratins. For each sample, we used the Crux pipeline [24] to determine protein abundance with a semi-quantitative approach. We used the Normalized Spectral Abundance Factor (NSAF) computed for each protein of the database to score its relative abundance across IPs. For validation of protein interactions, we tested IP fractions with either SAHH antibody or AHCYL1/SAHH-3 antibody.

### Detection of N6-methyladenosine in snRNA

Total RNA was separated on 10% PAGE–urea and transferred on a positively charged nylon membrane, prior to detection with an m6A antibody. We detected snRNA with northern blot and a pool of oligoribonucleotides end-labeled with ^32^P. Membranes were hybridized in ULTRAhyb-Oligo, washed following the manufacturer’s recommendations, and signals were recorded as described above. For transfected cells, each RNA sample was tested with m6A antibody and ran in parallel for northern blot. For each snRNA, we quantified m6A and normalized against the corresponding radioactive signal.

### Mapping of m6A in U1 snRNA

We followed the procedure described by Hong *et al.* [25], using 1.5 µg total RNA annealed to 1.5 × 10^5^ cpm oligonucleotide 5′-labeled [γ^32^P]-ATP, complementary to positions 13–33 of U1 snRNA. After 2 min at 90 °C, the hybridization mix was placed for 2 min at 50 °C, and 10 U Superscript IV reverse transcriptase (Invitrogen) was added in RT (reverse transcription) buffer (50 mM Tris–HCl, 4 mM MgCl_2_, 10 mM DTT, 50 mM KCl, 30 µM dATP, 30 µM dGTP, 30 µM dCTP, pH 8.3) in the presence of 30 µM of either dTTP or 4SedTTP (Jena Biosciences). After 20 min of incubation at 50 °C, cDNA synthesis was stopped by the addition of urea gel loading buffer and denatured elongation products were resolved on 20% PAGE–urea sequencing gels. Signals were recorded as described above.

## Results

### Duplication of U2AF subunit genes coincides with the emergence of a new class of introns

**Figure 2.**
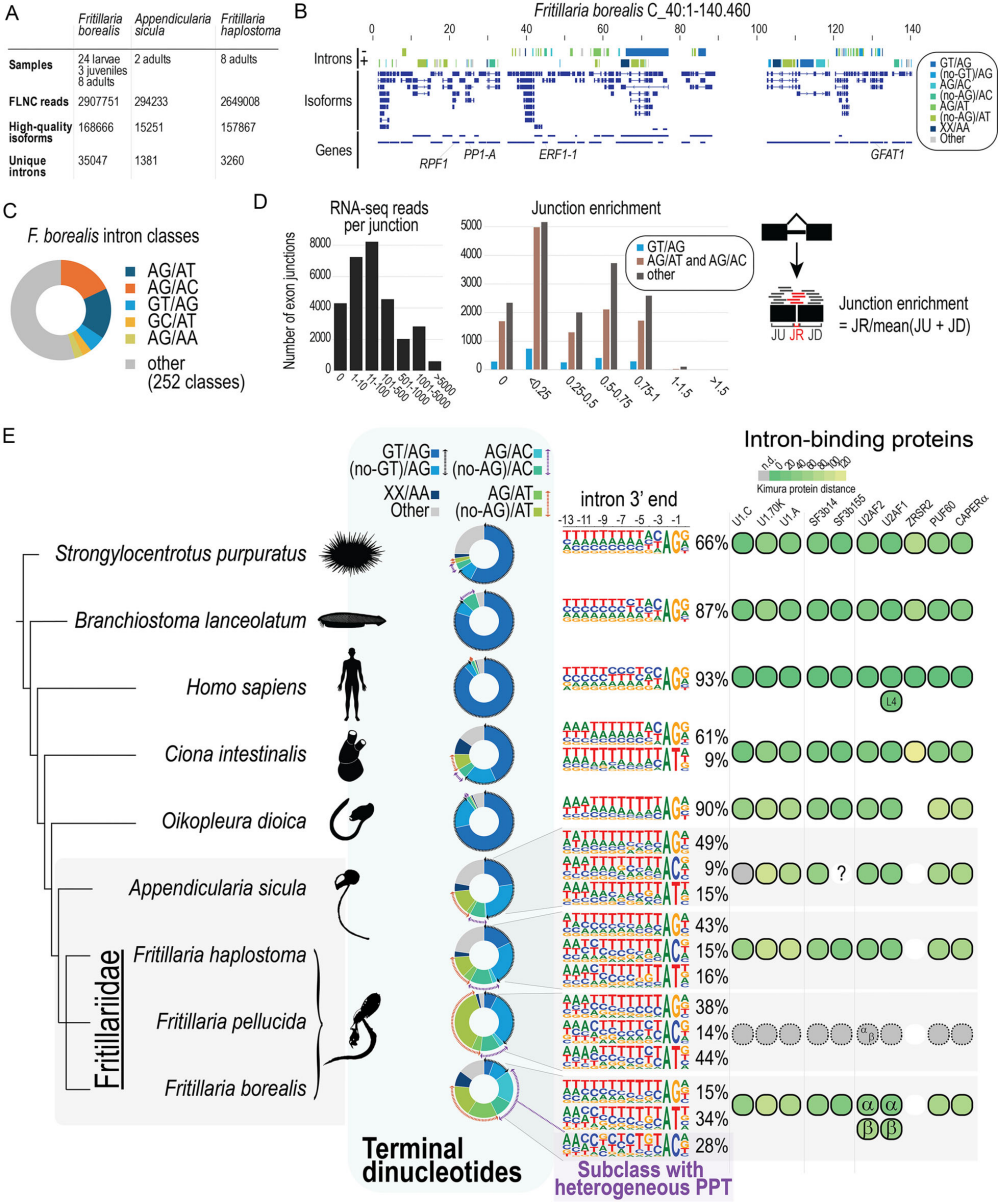
Evolution of splice sites and U2AF. (**A**) Samples, sequencing results, and number of annotated introns obtained with PacBio Iso-seq. (**B**) Genome browser snapshot showing alignment of PacBio isoforms, mapped introns, and gene predictions in a contig of the *F. borealis* genome assembly. (**C**) Classification of annotated *F. borealis* introns based on terminal dinucleotides. (**D**) Illumina split read alignment on *F. borealis* exon junctions. Left, total number of reads per junction counted across five RNA-seq libraries. Right, splice junction usage for classes of canonical and non-canonical introns. Values shown in the graph correspond to maximum junction enrichment measured across five RNA-seq libraries. (**E**) Evolution of intron classes. Species were placed on the tree based on published phylogenies [18]. For each genome, introns were grouped into classes based on their terminal dinucleotides. Statistics for *S. purpuratus, B. lanceolatum, H. sapiens*, and *C. intestinalis* were retrieved from Minor Intron Database [27]. Logos show nucleotide composition at the 3′ end of main intron classes, along with their frequency. Dots represent gene copies identified for proteins that bind splicing signals, with full borders showing evidence gained with transcripts and genomic DNA, and dashed borders showing evidence from genomic DNA only. We could not find a SF3b155 gene in *A. sicula*, possibly due to incomplete genomes and transcriptomes. Greek alphabet letters represent *F. borealis* U2AF paralogues and hybrid U2AF2 found in *F. pellucida*. The color palette represents the evolutionary distance of each gene compared to the human sequence, calculated using the Kimura multiple substitution correction algorithm for protein sequences (Supplementary Fig. S2).

Genetic resources for *F. borealis* were initially limited by specimen availability, preventing the production of robust and contiguous genome assemblies [18]. Recent improvements in animal culture [16] allowed us to access different stages during the lifecycle, and to collect the material required for long-read sequencing of cDNA and genomic DNA. We used PacBio Iso-seq to sequence full-length transcripts from *F. borealis* individuals corresponding to hatched larvae and adults (Fig. 2A, extended methods 6). Higher contiguity in the latest assembly (extended methods 7 and Supplementary Table S1) proved useful for full-length alignment of cDNA. After mapping transcripts on the genome (Fig. 2B, extended methods 8), introns borders were identified using hallmark sequences flanking alignment gaps in the cDNA. For the major classes of non-canonical introns AG/AC and AG/AT, as previously observed [4], a YAY triplet is often present immediately upstream of the 5′ss and almost invariably, a YAY triplet also composes the last three bases that include the 3′ss (Supplementary Fig. S1). The presence of a YAY triplet is infrequent upstream canonical GT/AG introns, and we used this signature to precisely map other classes of non-canonical introns. Among the 31 093 intron borders mapped in the genome, we counted only a small fraction of canonical introns (5%), while AG/AC and AG/AT were the non-canonical termini observed most frequently (respectively, 18% and 16%; Fig. 2C). We examined the coverage of exon junctions in a collection of Illumina RNA-seq reads (extended methods 6 and 8), which corresponds to a partial representation of transcriptomes sequenced with PacBio Iso-seq. The dataset confirmed that most junctions mapped with PacBio correspond to a splicing event, with 85% of annotated junctions supported by at least five reads (Fig. 2D). Exon junctions with lower coverage or without supporting reads may represent rare splice variants present only in samples sequenced with PacBio Iso-seq. Split read enrichment at exon junctions did not reveal significant differences associated with intron classes, suggesting that splicing efficiency remains similar between canonical and non-canonical introns.

To gain better insight into intron evolution in larvacean tunicates, we also sequenced DNA and RNA of two uncultured Fritillariidae species, *F. haplostoma* and *A. sicula* [16]. These genomes remain of lower quality, which prevented to reveal number of introns comparable to *F. borealis*. However, long-read transcriptomes obtained with all three species allowed us to find gene orthologs and to annotate introns in *F. pellucida*, another species whose genome was sequenced during our previous study [4], but for which no RNA-seq data is available.

We next compared our dataset to a recent survey of U2- and U12-type introns [7]. We found a higher diversity of intron termini among the six Tunicate genomes we examined than in other chordates. In agreement with earlier results [4], intron diversity was highest in the Fritillariidae genomes (Fig. 2E). In four Tunicate genomes, the most frequent 3′ss corresponds to the dinucleotide AG present in GT/AG and GV/AG introns, with frequencies ranging from 43% to 90%. The situation is different in the *F. pellucida* and *F. borealis* genomes, where the 3′AG is less frequent than other termini (respectively, 38% and 15%). Instead, these genomes most frequently use a 3′AT, found in 44% of *F. pellucida* introns and 34% of *F. borealis* introns. In *F. borealis*, 3′AC is the second-highest 3′ss in frequency (28%), while in other species it does not exceed 15%. Another remarkable feature of the *F. borealis* subclass of 3′AC introns is a heterogeneous PPT often interrupted by purines, while for all other genomes, the uridine-rich PPT is a typical feature of most introns, including those ending with 3′AC or 3′AT.

**Figure 3.**
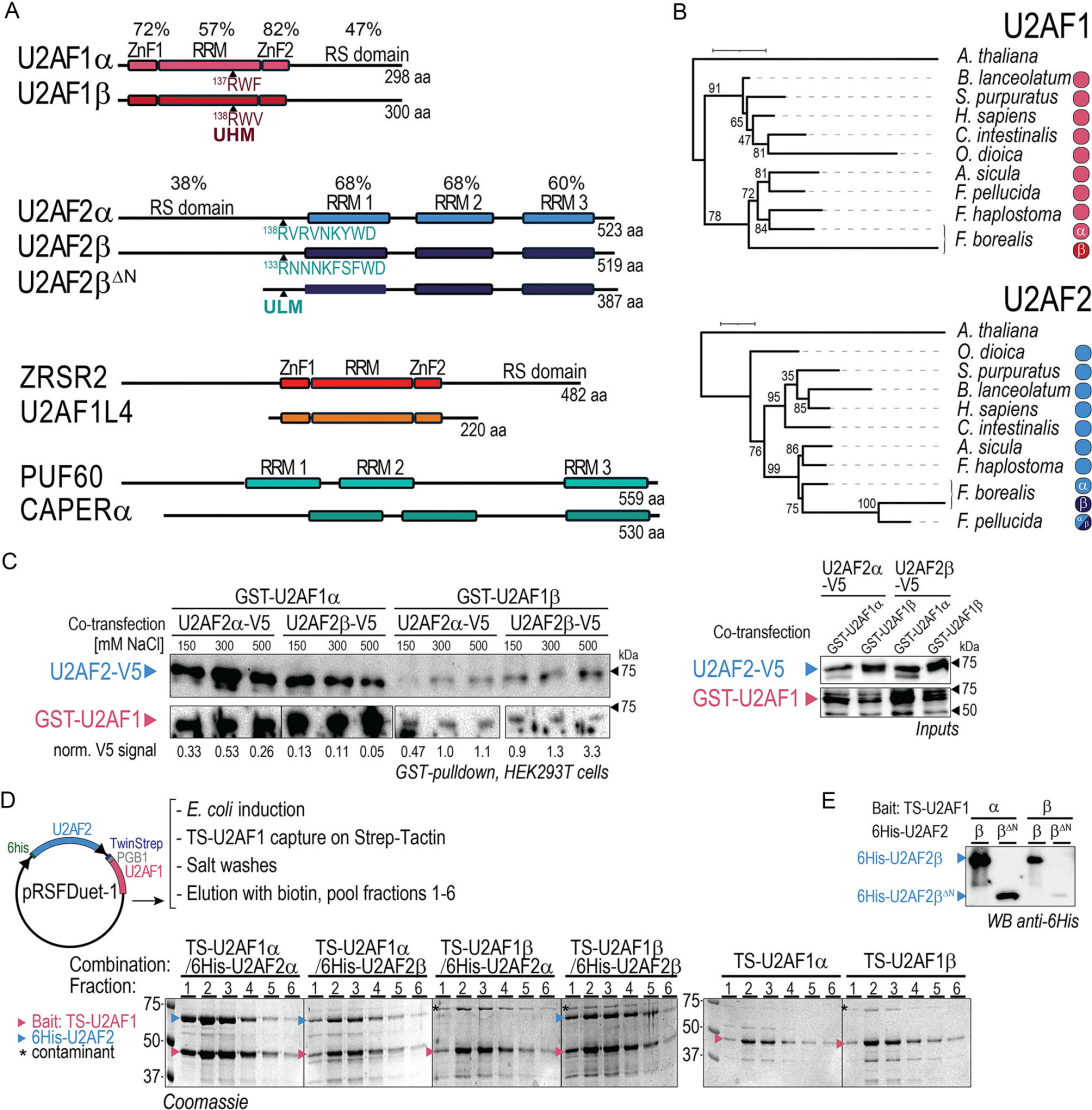
Protein–protein interactions between *F. borealis* U2AF subunits. (**A**) Domains and interaction modules in *F. borealis* U2AF1/2 paralogs α and β (top), and U2AF1/2-like proteins (bottom). Pairwise identity between paralogs is shown above domain names. (**B**) Maximum-likelihood phylogeny of U2AF subunits. Scales correspond to 0.1 substitution per site. (**C**) GST-pulldown assays in HEK293T cells. U2AF1 baits were co-expressed with U2AF2. Left panel is a western blot image showing U2AF2-V5 and GST-U2AF1 proteins remaining after pulldown, under increasing salt concentration. Values correspond to the ratio of V5/GST signal intensities acquired with separate exposures. Right panel is a western blot image showing expression of V5- or GST-tagged inputs. (**D**) Copurification of U2AF subunits expressed in *E. coli*. On the left, map of the construct used for tandem expression of U2AF subunits and outline of the purification procedure. Gel images correspond to Coomassie-stained SDS–PAGE showing co-eluted U2AF subunits (series on the left) and U2AF1 subunits eluted after separate induction. See Supplementary Fig. S8 for separate induction of U2AF2 subunits. Copurification failed only for the U2AF1β/2α combination. (**E**) The RS domain contributes to U2AF1β/2β protein interaction. We detected U2AF2 proteins that co-eluted with U2AF1 baits with an antibody against the 6His tag. Comparison of lanes 2 and 4 show that deletion of the RS domain specifically reduces the amount of U2AF2β that copurifies with the U2AF1β bait.

We examined the complement of spliceosome genes in these new genomes (extended methods 9), and we could identify all major components of the U1 snRNP and the U2AF complex. In the *F. borealis* genome, however, we found an extra copy for each U2AF1 and U2AF2 subunit (Fig. 2E, right panel). Several indications allowed us to distinguish these pairs of genes from other U2AF paralogs known in deuterostomes (Fig. 3A), showing they likely correspond to a recent duplication in the *F. borealis* lineage. Consistent with the loss of the minor spliceosome, we could not find orthologs of ZRSR2 in larvacean genomes. The *F. borealis* U2AF1 genes lack the N-terminal disordered domain found in ZRSR2, and their protein sequence is closer to deuterostome U2AF1. The U2AF1L4 is a mammalian paralog unrelated to the U2AF1 duplication in *F. borealis*. In *F. borealis*, U2AF2 genes conserve the characteristic protein domain arrangement of canonical U2AF2 and they lack the linker domain found between RRM2 and RRM3 of U2AF2-like proteins PUF60 and CAPERα, genes whose orthologs could be found in the *F. borealis* genome (Fig. 2E, right panel). Versions named U2AF1α and U2AF2α share high sequence identity with single copy genes found in other deuterostomes, while versions U2AF1β and U2AF2β show higher divergence, especially outside RNA-binding domains (Fig. 3A and B). For each U2AF subunit, both paralogs are transcribed at comparable levels in the transcriptome and *in situ*, suggesting these genes could still have a functional impact after duplication (Supplementary Fig. S3). In *F. pellucida*, U2AF1 is homologous to U2AF1α, but U2AF2 corresponds to a chimera between U2AF2α and U2AF2β, indicating that the common ancestor with *F. borealis* possessed two copies, which merged into one in *F. pellucida* (Fig. 2E and Supplementary Fig. S4).

### Divergent U2AF subunits form heterodimers with novel RNA binding properties

In principle, four different U2AF heterodimers could form if each subunit paralog was expressed in the same *F. borealis* cell. If RNA binding preferences differed between these proteins, the variety of U2AFs could be instrumental for recognizing the diverse 3′ss of *F. borealis*. We first tested whether U2AF1 and U2AF2 paralogs could bind to each other. U2AF assembly is mediated by interactions between the U2AF homology motif (UHM) of U2AF1 and the U2AF ligand motif (ULM) of U2AF2 [2] (Fig. 3A). Using GST-U2AF1α as bait, we could recover both versions of U2AF2 after co-expression in mammalian cell culture (Fig. 3C and Supplementary Fig. S5). Cellular expression levels were equivalent for U2AF2 paralogs, and U2AF1α interacted better with U2AF2α than U2AF2β, as we could detect 10%–35% excess protein bound on glutathione beads. The bait U2AF1β bound less efficiently to U2AF2 paralogs, possibly a consequence of changing the conserved phenylalanine by a valine in U2AF1β UHM, that causes a loss of π-interaction with the ULM (Supplementary Figs S6 and  S7). However, U2AF1β binds better to U2AF2β than U2AF2α, and monovalent salts increase the affinity of the interaction. This is unlike the interaction observed between U2AF1α and U2AF2β, which is weakened by increasing salt concentration. These experiments show that *F. borealis* U2AF subunits conserve the ability to interact after expression in mammalian cell lines. In the next sections of the results, we describe how we used this heterologous system to characterize U2AF2 interaction with endogenous RNA and proteins.

We transformed *E. coli* with protein expression vectors carrying the four combinations of U2AF1 and U2AF2 paralogs (Fig. 3D, extended methods 10). We could not detect U2AF2α protein expression when the gene was co-expressed with U2AF1β, but combinations corresponding to U2AF1α/2α, U2AF1α/2β, and U2AF1β/2β were successfully expressed (Supplementary Fig. S8A). After passing bacterial lysates on an affinity resin to capture the protein tag fused to U2AF1, we could also recover the U2AF2 subunit in all three cases (Fig. 3D). Control experiments performed in the absence of tagged U2AF1 show that U2AF2 subunits do not bind to the affinity resin (Supplementary Fig. S8B), indicating that co-purification of U2AF2 with U2AF1 could involve protein–protein interactions. Removing the N-terminal part of U2AF2β significantly reduced binding to U2AF1β but not to U2AF1α (Fig. 3E), showing that interactions established with the RS domain of U2AF2β compensate for reduced affinity caused by UHM divergence in U2AF1β.

**Figure 4.**
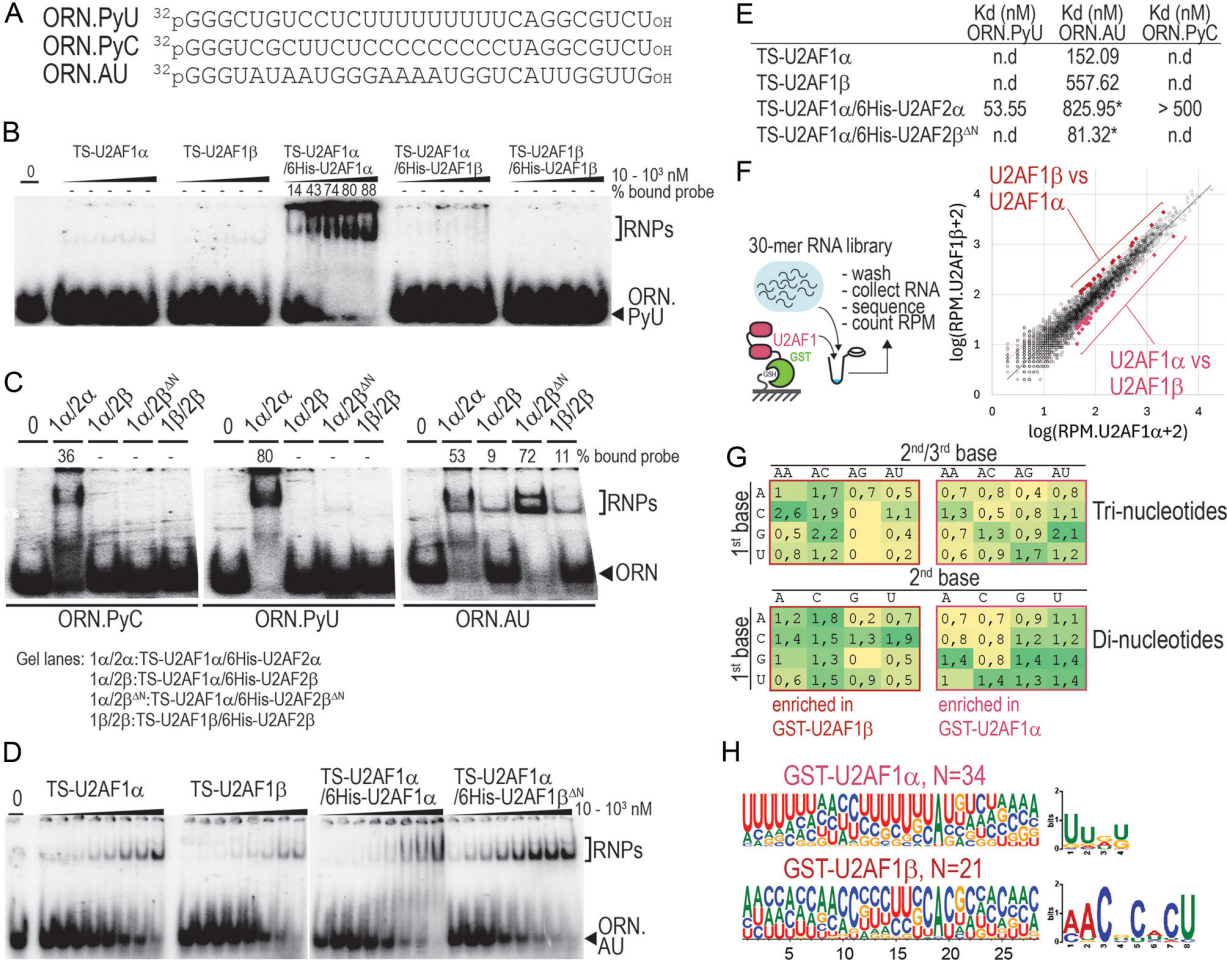
RNA-binding assays with *F. borealis* U2AF. (**A**) Sequence of RNA probes used for EMSA. (**B**) EMSA with ORN.PyU. Quantification of bound probe is reported only for values superior to 5%. Arrowhead shows unbound probe; bracket indicates probe present in RNPs. (**C**) EMSA comparing binding efficiency to different probes. We used 330 nM U2AF per assay. (**D**) EMSA with ORN.AU. (**E**) U2AF dissociation constants determined for ORN.PyU and ORN.AU. Values marked with a star correspond to the average of two titrations. (**F**) RNAcompete experiments with U2AF1. Left side, schematic of the approach; right side, plot of read counts showing differential enrichment with GST-U2AF1β (*x*-axis) and with GST-U2AF1β (*y*-axis). Colored diamonds represent groups of high affinity RNA selected for sequence analysis. (**G**) Frequency of three- and two-mer motifs in high affinity RNA. Values correspond to the average frequency in collected RNA relative to the average frequency measured in the input library. (**H**) Sequence logos produced by aligning high affinity RNA collected with GST-U2AF1α or GST-U2AF1β (left side) and over-represented motifs detected with STREME (right panel).

We tested RNA binding first with EMSA (extended methods 11) and oligoribonucleotide probes designed to mimic an intronic 3′AG, preceded either by a U-rich (ORN.PyU) or a C-rich PPT (ORN.PyC). We tested a third probe derived from an *F. borealis* intron, where the 3′ss is an AU, preceded by a purine-rich sequence (ORN.AU) (Fig. 4A). Consistent with preferences reported for human U2AF2 [5], proteins eluted from the U2AF1α/2α co-purification interacted best with ORN.PyU, followed by ORN.AU and ORN.PyC (Fig. 4B and C). Other protein preparations, which include U2AF1α/2β, U2AF1β/2β, and single U2AF1 paralogs, interacted better with ORN.AU than with other probes, whose fraction bound to protein never exceeded 5% (Fig. 4C, gel on the right side). It remains unclear whether reduced affinity for pyrimidine-rich probes is caused by substitutions in RNA-binding domains of U2AF2β (Supplementary Fig. S9). However, the affinity for ORN.AU improved dramatically when testing a U2AF1α/2β^ΔN^ preparation obtained by co-purifying U2AF1α with a U2AF2β^ΔN^ mutant missing the N-terminal part of U2AF2β (Fig. 4C–E).

The U2AF1 paralogs differ by a few substitutions in the first zinc finger involved in RNA binding (Supplementary Figs S6 and S10). We used an approach based on RNAcompete [26] (extended methods 12) to test the interaction of U2AF1 immobilized on beads with a collection of short RNA derived from *F. borealis* 3′ intron–exon junctions (Fig. 4F). After sequencing bound RNA, we first examined the frequency of triplets with central adenine. Compared to U2AF1α, triplets ending with AG are largely depleted in RNA recovered with U2AF1β, whereas those ending with AC are most frequent (Fig. 4G). In U2AF1α-bound RNA, UAG triplets are more frequent than other triplets ending with AG, suggesting a conserved ability to discriminate U_−3_. Dinucleotide frequencies revealed a preference for C in U2AF1β-bound RNA and for G and U in U2AF1α-bound RNA, and this composition bias was also revealed with other methods of motif analysis (Fig. 4H). Similar preferences were observed with U2AF1β when comparing it to the *O. dioica* U2AF1 ortholog (Supplementary Fig. S11). Taken together, these indications about RNA-binding preferences suggest that U2AF1β could be involved in the processing of introns ending with 3′AC.

### U2AF2α and U2AF2β induce distinct splicing events in mammalian cells

At least three stable combinations of U2AF may function in *F. borealis*, presumably with different RNA targets. Addressing protein function in *F. borealis* would imply developing several experimental tools, and we decided to gain rapid functional clues by testing whether the expression of *F. borealis* U2AF can influence splicing in mammalian cells.

**Figure 5.**
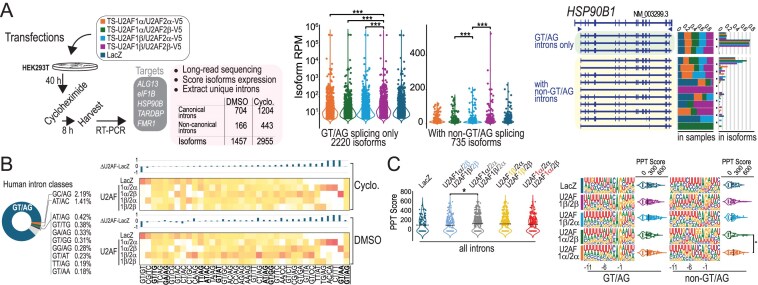
Impact of *F. borealis* U2AF expression on splicing. (**A**) Combinations of subunit paralogs induce the expression of distinct isoforms. Panel on the left side describes the experimental approach and the total number of introns and isoforms discovered in the cDNA of either control cells or cells treated with cycloheximide. Scatter plots show, for treated cells, read count of isoforms produced only by canonical splicing events (left), or by the splicing of at least one non-canonical intron (right). Stars show Wilcoxon signed-rank test values (*, >0.05; **, >0.01; ***, >0.005). For each top-expressed isoform of the HSP90B1 gene, the panel on the right side indicates frequency among samples or among isoforms of the same sample. Arrowheads show primers used for RT-PCR. (**B**) Frequency of non-canonical splicing. The top chart shows the frequency of non-GT/AG intron classes in the human genome, based on Olthof *et al*. [27]. Heatmaps show the average expression of isoforms produced by at least one non-GT/AG splicing in transfected cells treated or not with cycloheximide. For each intron class, we report the normalized expression of all associated splice variants. The ΔU2AF-LacZ values displayed on top of heatmaps indicate the difference of expression between the strongest U2AF samples and the control divided by the sum of all expressions. (**C**) U2AF2β-V5 induces splicing at weak PPT. Top plot shows PPT scores of unique introns found processed with different subunit paralogs. Bottom plot displays PPT scores for distinct U2AF combinations, next to the sequence logo of introns aligned on their 3′ss. Dashed lines show the dataset median.

To maximize the detection of rare and aberrant splicing events, we focused on the splicing of a set of highly expressed genes after blocking translation-coupled nonsense-mediated decay with cycloheximide (Fig. 5A and extended methods 13). The treatment permitted to raise the number of detected splicing events at GT/AG borders by 1.7-fold and at other borders by 2.7-fold. Sequencing target cDNA with long-read approaches revealed that U2AF1β/2β expression significantly increases splicing at non-GT/AG borders, even though the isoforms produced by these events remain rare. Non-GT/AG splicing events induced by U2AF expression include some typical classes of human minor introns [27]. Transfection of U2AF1α/2β resulted in a strong activation of GT/AA introns, whose splicing is otherwise undetected in the LacZ control (Fig. 5B, right side of the heatmap). We could detect isoforms produced by the splicing of other minor intron classes such as AT/AC and AT/AG in LacZ transfections, but their expression was generally lower compared to U2AF transfections. Each U2AF combination could be linked to the expression of a distinct set of splicing isoforms, and by examining the unique introns spliced in one transfection but left unprocessed in others, we could link differences at the PPT sequence with the expression of U2AF2 paralogs (Fig. 5C). We scored these differences by measuring the density of uridines in the 25 bases upstream of the last intron position [28]. The expression of U2AF2β was associated with the splicing of introns with weaker PPTs, while stronger PPTs were found in introns processed under U2AF2α expression.

**Figure 6.**
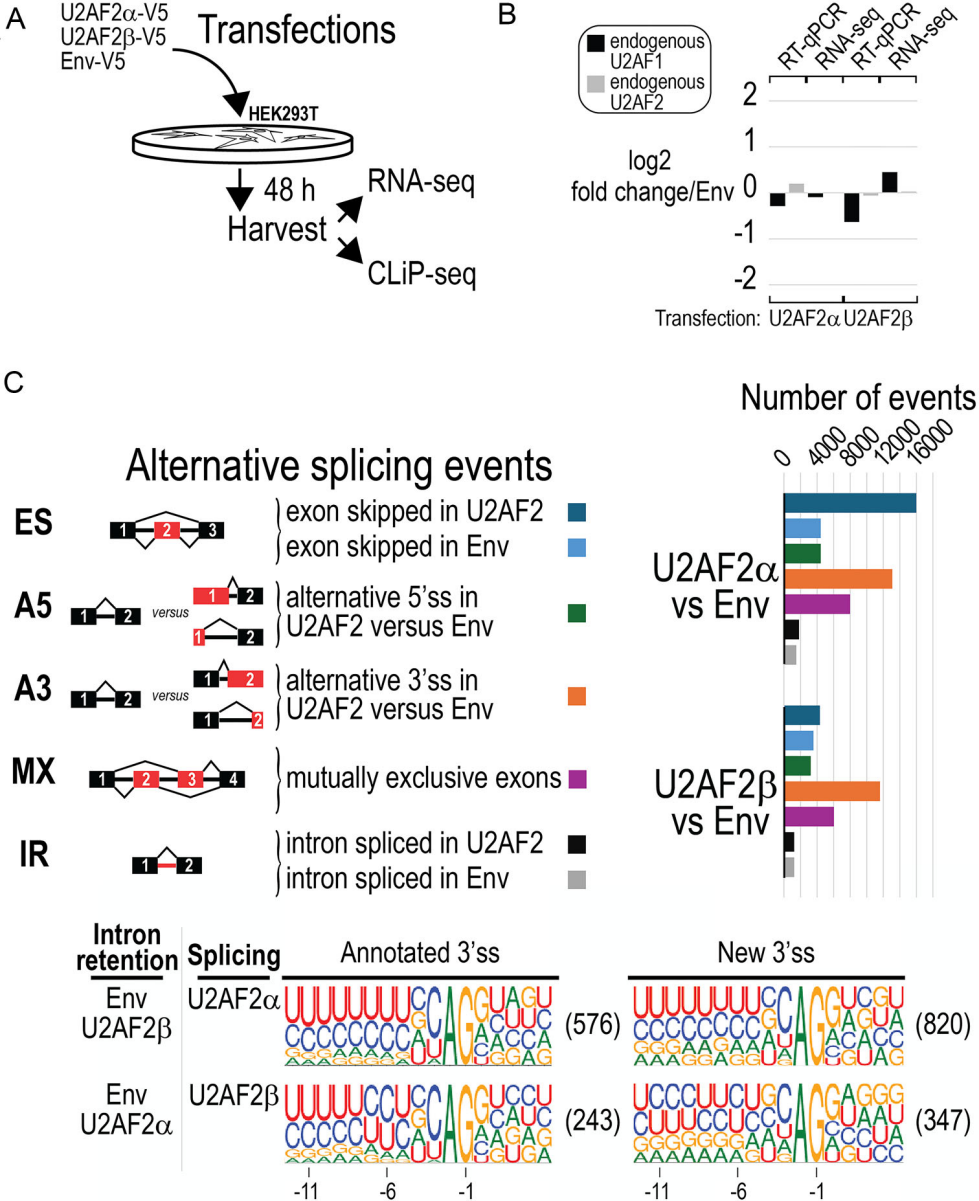
Impact of *F. borealis* U2AF2-V5 expression on splicing in mammalian cells. (**A**) Approach followed to characterize splicing events and protein binding on RNA (see Fig. 7) in U2AF2-transfected cells. (**B**) Expression of endogenous U2AF genes in cells transfected with *F. borealis* U2AF2-V5, compared to control cells transfected with Env-V5. Changes in expression level were measured either with the RNA-seq data or with RT-qPCR performed on an independent series of transfections. (**C**) Top, AS events detected by rMATS on the RNA-seq data, and number of classified events detected in U2AF2-V5 transfections but absent from Env-V5 controls. Bottom, sequence logos produced with 3′ss associated with IR events. Logos correspond to alignments of 3′ss spliced in a U2AF2-V5 transfection but left unprocessed in other samples.

We examined the transcriptome-wide impact of *F. borealis* U2AF2 paralogs, compared to a control Env gene [17] (Fig. 6A and extended methods 14). We could document the stronger effect of U2AF2α on gene expression (Supplementary Fig. S12A) but GO term analysis did not reveal a specific regulation of transcription programs. Over a third of top differentially expressed genes between U2AF2α and U2AF2β correspond to non-coding genes (Supplementary Fig. S12B). Expression levels of endogenous U2AF genes remained stable between Env- and U2AF2-transfected cells (Fig. 6B and Supplementary Fig. S12C). We observed antagonistic effects on the antisense lncRNA transcribed from SMN1 and SMN2 genes, with SMN-AS1 overexpressed in U2AF2α transfections and SMN2-AS overexpressed in U2AF2β transfections. It is unclear whether these differences arise during lncRNA processing or whether they are related to the expression of SMN1 and SMN2 genes [29]. After mapping reads with the human genome annotation, rMATS revealed 42 931 AS events associated with U2AF2α expression, and 26 373 AS events associated with U2AF2β expression. Excess events detected with U2AF2α are largely contributed by exon skipping (ES) (Fig. 6C). Human U2AF2 bound on deep intronic sites can induce skipping of the following exon by interfering with 3′ss selection [5]. While transfected U2AF2α may participate to this mechanism, fewer ES registered with U2AF2β suggest it does not contribute to the same extent—possibly due to different affinity for RNA. We could notice differences at the 3′ss when examining intron retention (IR). Introns removed only during U2AF2α expression possess uridine-rich PPTs, while introns spliced during U2AF2β expression show higher heterogeneity.

**Figure 7.**
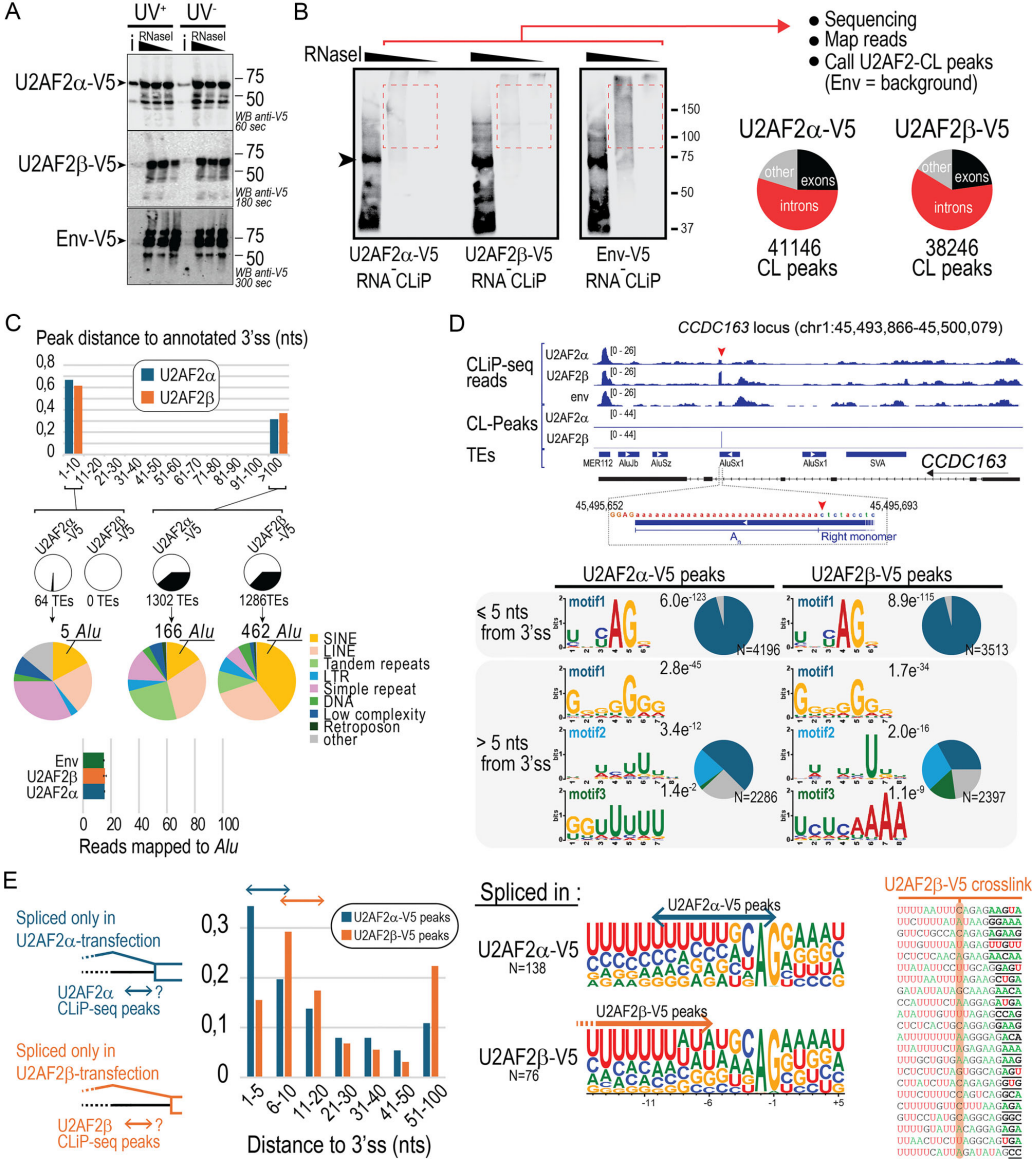
Target introns of *F. borealis* U2AF2 in HEK293T cells. (**A**) IP results during CLiP-seq experiments. Cell monolayers were irradiated (lanes 1–4) or left untreated (lanes 5–8) prior harvesting, and cell lysates were treated with increasing concentration of RNAse I (lanes 3 and 4, and 6–8) prior to incubation with beads. Lanes 1 and 5 were loaded with 8% of the input prior to RNAse treatment, lanes 3 and 4, and 5–8 were loaded with 40% of the material retained on beads coupled to an anti-V5 Ig. After electrophoresis, proteins were transferred on a nitrocellulose membrane and revealed with an anti-V5 western blot. Arrows show the band with the expected size of the over-expressed protein. (**B**) Nitrocellulose membrane with radiolabeled, crosslinked protein–RNA complexes transferred after fragmentation with RNase, IP, and electrophoresis. Dashed squares show areas selected for RNA extraction; arrow shows complexes fully digested with RNase (U2AF2 and Env size is between 65–70 kDa). Pie charts show the distribution of U2AF2 crosslink (CL) peaks in exons, introns, and other annotated genomic features. (**C**) Top, distribution of U2AF2 peaks inside annotated introns. Nearly 60% peaks located 100 nt away from the 3′ss are matching to an annotated transposable element (TE), predominantly *Alu* in the case of U2AF2β peaks. Bottom, fraction of reads mapping to *Alu* in RNA-seq samples. (**D**) Left, genome browser screenshot showing CLiP-seq reads mapped on the *CCDC163* locus of the human genome; crosslink peaks; and TEs and *Alu* with their orientation. The U2AF2β crosslink site (arrowhead) in *Alu* sequence is detailed. Right, results of STREME analysis showing motifs differentially enriched between U2AF2α and U2AF2β in an 11-nt window centered on crosslink peaks. Pie charts show frequencies of significant motifs in input sequences; gray area corresponds to non-significant motifs. (**E**) U2AF2β binding to A-rich intron ends is linked to 3′ss selection. Distance of U2AF2 binding site from novel 3′ss induced with U2AF2 expression (see Fig. 6). Sequence logos were produced by aligning 3′ss for which U2AF2 peaks are found 20 nt upstream. On the right, intron ends have been aligned on the U2AF2β crosslink; the downstream exon is underlined.

We used Cross-Linking and Immunoprecipitation followed by Sequencing (CLiP-seq) to recover RNA bound to transfected U2AF2 (Fig. 7A and extended methods 15). Consistent with results reported for human U2AF2 [5, 30], crosslinks mapped predominantly (60%) to annotated introns and the majority of intronic peaks mapped to PPTs close from the 3′ss (Fig. 7B). Despite poor affinity for U-rich RNA *in vitro*, U2AF2β can be recruited to PPTs of annotated introns, possibly through heterodimerization with endogenous U2AF1. However, U2AF2β crosslinks located further upstream of the 3′ss clearly identified binding sites inside *Alu* elements (Fig. 7C). *Alu* expression remained stable across different transfections, showing these crosslinks reflect genuine binding preference rather than background caused by increased expression. Sequences surrounding the crosslink consist in pyrimidines at the 5′ half and the poly-A stretch of *Alu* at the 3′ half (Fig. 7D), a composition that also appears among U2AF2β binding sites in exons (Supplementary Fig. S13A).

Repeated and low-complexity sequences made it difficult to unambiguously assign splicing events to U2AF2β binding sites discovered in *Alu*. For most binding sites located close to intron ends, bias linked to 3′ss conservation prevented to distinguish sequences preferred by U2AF2α from those preferred by U2AF2β. We decided to examine the novel 3′ss identified with rMATS, which are specifically processed under U2AF2 paralog expression (Fig. 7E). For each 3′ss we measured, within the 100 nt upstream, the distance to the closest U2AF2 crosslinks. U2AF2α binding sites were generally found very close (<5 nt) from 3′ss, whereas U2AF2β binding frequently occurred between 6–20 nt upstream. These differences disappeared when comparing crosslinks obtained with a U2AF2 paralog, to the 3′ss processed with the other paralog (Supplementary Fig. S13B). Adenines are more frequent upstream of the 3′ss spliced with U2AF2β and based on their position downstream of the crosslink, we infer they correspond to the 3′ half of U2AF2β binding sites, consistent with the motif revealed in *Alu*. While those adenine-rich sites could explain the preference for the probe ORN.AU *in vitro*, U2AF2β interaction with other sequences suggested that RNA recognition could be regulated by other proteins in mammalian cells.

### U2AF2β binds to AHCYL1 in HEK293T cells

**Figure 8.**
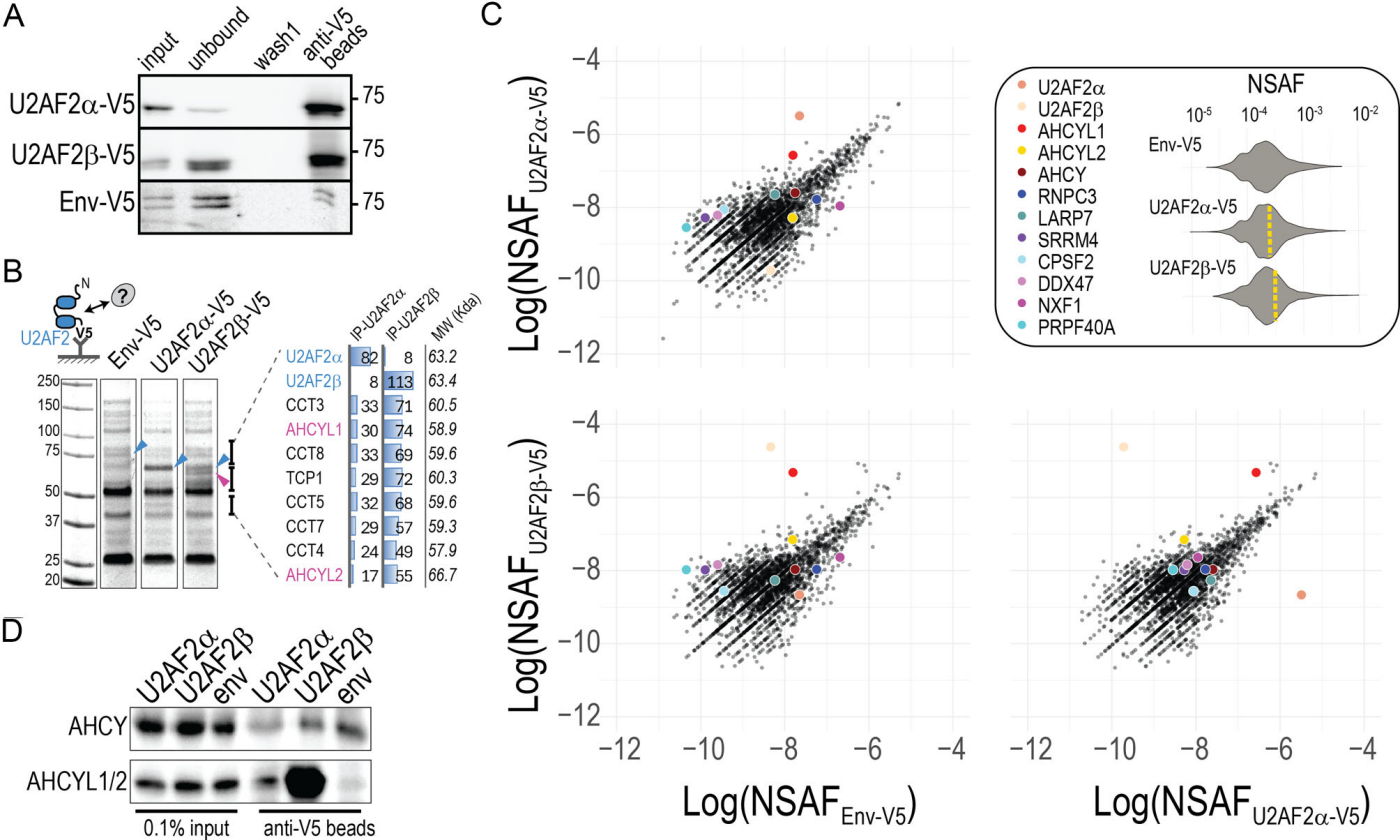
AHCYL1 interacts with U2AF2β-V5 and regulates m6A levels on snRNA. (**A**) IP results. Panels show detection of V5-tagged proteins with western blot, during IPs using lysates of HEK293T cells transfected with either *F. borealis* U2AF2-V5 or Env-V5. (**B**) Identification of *F. borealis* U2AF2-bound partners with co-IP. Coomassie-stained gel shows proteins bound to V5-tagged U2AF2 after expression in HEK293T cells and IP. Brackets show the areas analyzed with MS. The panel on the right shows proteins with most diverse peptides for each U2AF2 co-IPs. Blue and pink arrows show bands corresponding to U2AF2-V5 baits and AHCYL proteins, respectively. (**C**) Relative abundance of proteins in MS datasets. Scatterplots show, for each protein identified with MS, the variation of NSAF between the three co-IPs. Colored dots correspond to a selection of splicing-related proteins and AHCY homologs, present above the 90th percentile (Q90) of proteins enriched in U2AF2 co-IPs compared to Env co-IP. Violin plots represent the sample distribution of NSAF score, with dotted line showing the lowest value found in the Q90 selection. (**D**) Validation of the interaction between U2AF2-V5 and AHCY-like proteins. Lanes 1–3: cell lysates expressing baits; lanes 4–6: material recovered with IP. Samples were tested with an AHCY antibody (top) or an antibody recognizing both AHCYL1 and AHCYL2 (bottom).

A functional divergence of U2AF2 paralogs in *F. borealis* could involve interactions with different protein partners. By using a co-immunoprecipitation (co-IP) assay to recover proteins bound to U2AF2α and U2AF2β in mammalian cells (Fig. 8A and extended methods 16), we expected to identify candidates with conserved homologs in *F. borealis*, which could provide additional clues about the function of U2AF2 in this organism. Mass spectrometry (MS) identified several proteins whose relative abundance is significantly higher (>4.8-fold change in spectral abundance) in U2AF2 co-IPs, compared to an Env co-IP (Fig. 8B and C). Proteins enriched in both U2AF2α and U2AF2β co-IPs include known regulators of splicing such as SRRM4 and PRPF40A [31]. Proteins that were preferentially enriched in U2AF2β included adenosine homocysteinase (AHCY)-like proteins. Proteins AHCYL1 and AHCYL2 (respectively known as SAHH2/IRBIT and SAHH3) share strong sequence identity and they are homologs of AHCY (also known as SAHH). Diagnostic peptides identified with MS suggest AHCYL1 as the primary partner of U2AF2β. AHCYL1/2 has also been reported to interact with AHCY. By using specific antibodies, we could confirm that U2AF2β specifically interacts with AHCYL1/2, but not AHCY (Fig. 8D).

The AHCY enzyme contributes to stabilizing cellular pools of SAM, the substrate of methyltransferases. AHCYL1/2 are considered catalytically inactive, and while they could interfere with AHCY function, the impact on methylation is debated [32]. Interestingly, AHCYL1/2 bind to ZCCHC4, the methyltransferase responsible for N6-methyladenosine (m6A) modification of 28S rRNA [33]. Recent studies also showed how m6A could regulate splicing [34, 35], and we decided to check whether its presence is impacted by AHCYL1 expression. We used CRISPR/cas9 to introduce double-stranded breaks in the AHCYL1 gene of HEK293T cells, and we selected AHCYL1 knockout cells that integrated a puromycin resistance gene during DNA repair. After several subculture pass, we tested for the presence of m6A using an immunoblot assay, but we could not observe significant difference between knockout and control cells on m6A levels present either on the snRNA or on poly-adenylated RNA (Supplementary Fig. S14).

### Larvacean snRNA shows specific N6-methyladenosine modification patterns

The sequence and secondary structure of U2-type snRNA remain highly conserved across deuterosteomes [4, 13], consistent with the critical function of these molecules during splicing of GT/AG introns. However, for genomes with an abundance of non-GT/AG introns, it remains unclear how sequence motifs that conserved a complementarity to canonical splice sites—such as the 5′ end of U1 snRNA—could bind to more divergent introns. We hypothesized that chemical modifications present on RNA could mediate the recognition of non-canonical splice sites and decided to test the presence of m6A on snRNA extracted from a variety of organisms, including the larvacean *Oikopleura dioica* and the ascidian *Ciona intestinalis* (extended methods 17).

**Figure 9.**
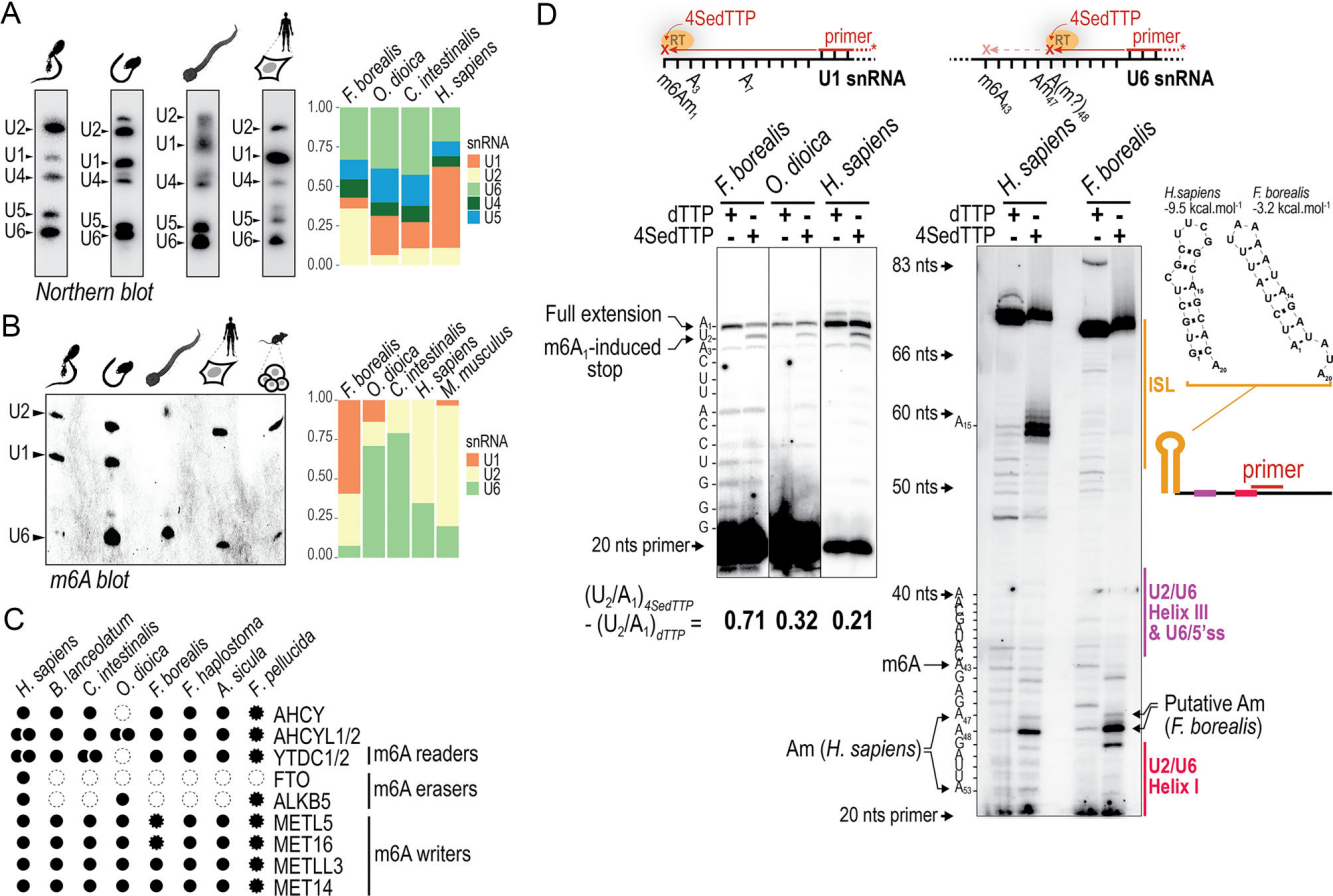
Divergent m6A patterns in larvacean snRNA. (**A**) From left to right, U2-type snRNA identified in maturing adults of *F. borealis* and *O. dioica, C. intestinalis* larvae, and HEK293T cells; graph shows the relative signal intensity measured for each snRNA. (**B**) Detection of m6A on snRNA. Bands were identified based on northern blots; graph shows the distribution of m6A signal; and last lane shows results from mouse ES cells. (**C**) Conservation of m6A regulatory genes. Dots represent gene copy number, full border indicates evidence from transcripts and genomic DNA, and dashed border indicates evidence from genomic DNA only. Empty dots represent gene losses. (**D**) Mapping modified adenines at the 5′ end of U1 snRNA and in the ACAGAGA box of U6 snRNA. Schematics represent RT elongation of a radiolabeled primer annealed to either U1 or U6 snRNA (left and right side, respectively). On U1 snRNA, the 5′ terminal m6A induces RT stop in the presence of thymidine analog 4SedTTP. On U6 snRNA, 4SedTTP provokes RT stop at 2′-O-methylated adenines A_47_ and A_48_, and pauses provoked at m6A_43_ are weaker. Gel images shows RT elongation products. For U1 snRNA, we quantified the intensity of m6A-associated pause by measuring the ratio of cDNA stopped at position U_2_ of U1 snRNA relative to full-length cDNA. Bottom values show ratios measured with 4SedTTP minus ratios measured with dTTP.

We could identify U2-type snRNAs in all samples (Fig. 9A). The relative expression level of U1 snRNA appears lower in *F. borealis* compared to other species. While the reduced intensity could be related to the probe used in northern blot (labeling efficiency, for instance), we cannot exclude it reflects reduced U1 snRNA concentration, perhaps linked to different roles during splicing. Remarkably, signals corresponding to m6A modification were distributed differently in larvaceans (Fig. 9B and Supplementary Fig. S15A). First, m6A levels decreased considerably on *F. borealis* U6 snRNA. After testing two different samples of this species, we could show that U6 snRNA represents on average 8.5% of the total m6A signal, versus 51% in *O. dioica* and 32% in human. This is striking since the genome possesses known m6A writers, including the methyltransferase METTL16 responsible for modifying A_43_ in U6 snRNA [36] (Fig. 9C). Therefore, we cannot exclude that m6A loss on the U6 snRNA is indirectly linked to the activity of lineage-specific molecules, which could include U2AF2 paralogs. Second, both *F. borealis* and *O. dioica* showed prominent m6A signals on U1 snRNA, whereas using the same technique, the modification is detected in mammals only in an FTO^−/−^ background [37].

We mapped the modification on U1 and U6 snRNA with a primer extension technique [25] that relies on RT pauses induced by the incorporation of Se-modified deoxythymidine triphosphate (4SedTTP) opposite m6A sites (extended methods 18). During primer extension assays conducted on U1 snRNA extracted from either larvacean or HEK293T cells, we could observe an RT pause induced by the presence of 4SedTTP at position U_2_. We measured the strongest pauses on the *F. borealis* template, followed by the *O. dioica* template, and the weakest on the human template (Fig. 9D). While values obtained with larvacean templates appeared consistent with m6A signals measured with immunoblots (Fig. 9B), the value obtained with the *H. sapiens* template seemed at odds with the virtual absence of m6A in U1 snRNA of normal cells [37]. Nearly all human U1 snRNA molecules are modified with a 2′-O-methylation at the first adenine (Am_1_) [38] and in fact, results gained with U6 snRNA indicate that this modification can account for some of the abortive cDNA produced at the U_2_ position.

When applying the primer extension assay to human U6 snRNA, 4SedTTP-induced elongation pauses were largely uncorrelated with the N6-methyl at A_43_ (m6A_43_). Instead, we measured stronger pauses at downstream Am positions (A_47_, A_53_, A_70_) [38] and at the A_15_ present in the ISL structure (Fig. 9D and Supplementary Fig. S15B). The 2′-O-methylation on adenines decreases RT affinity for dTTP [39], suggesting that pauses associated with Am positions in our experiments are caused by misincorporation of 4SedTTP. While they provide an explanation to pauses associated with Am_1_ in human U1 snRNA, these results show that sensitivity of the 4SedTTP extension assay for detecting m6A can be significantly challenged by Am frequency and by the secondary structure of the template. These limitations make the immunoblot assay more reliable to quantify m6A levels in U6 snRNA. In the absence of secondary structure elements, we suppose that elongation pauses in the region 40–48 of *F. borealis* U6 snRNA are caused by adenine decorated with 4SedTTP-sensitive modifications which, for the positions homologous to A_47_ and A_48_ in human U6 snRNA, could correspond to Am rather than m6A.

## Discussion

By placing a variety of organisms under molecular scrutiny, recent genomics have led to fundamental discoveries about the origins of introns and the evolution of the spliceosome. Studying the repertoire of spliceosome genes in unicellular eukaryotes proved valuable for appreciating how splicing function is preserved during evolution and how its mechanisms have adapted to intron variety [14, 40]. Splicing flexibility has been discussed in the context of vertebrate genomes [41] and their degenerate splice sites. Here, we demonstrate that novel insights can be gained from larvacean tunicates, animals whose streamlined genomes have conserved a large part of the genetic equipment linked to chordate development. Our findings reveal mechanisms that could contribute to splicing flexibility in these organisms. The functional differences between U2AF that we revealed with heterologous assays remain to be confirmed with specific approaches in *F. borealis*. However, our results indicate that the functional divergence of splicing factors could be a key element behind the emergence of non-canonical splicing, and it is likely that other molecules than U2AF have evolved to recognize diverse classes of splice sites. On the other hand, changing the pattern of modified bases on snRNA could represent a simpler way, from an evolutionary perspective, to modulate the specific recognition of splice sites. The information gained with this study will open new perspectives about the evolvability of splicing across eukaryotes, and it could be instrumental for understanding the regulation of splicing in the complex genomes of mammals.

### The divergence of U2AF and its impact on 3′ss recognition

While U2AF1 and U2AF2 have been extensively studied in mammals, our results bring novel knowledge by showing how evolution can change the biochemistry of these crucial splicing factors. Our results already provide indications that U2AF divergence can be linked to novel splice sites. Biophysical approaches should reveal whether these new proteins recognize introns with the same mechanisms as human U2AF [42], and how binding dynamics are impacted by residues changed in U2AF1β and U2AF2β. The RS domain of human U2AF2 is required for binding to Prp19 [43], and our result suggests it could also determine the interactions of *F. borealis* U2AF with nascent pre-mRNA and the splicing machinery. The inhibitory effect of U2AF2β RS domain on RNA-binding could be controlled by phosphorylation, as was shown for SRSF1 [44]. U2AF2α maintains a strong affinity for RNA when the RS domain is present, and this is perhaps due to a proline stretch located a few positions upstream the ULM, which could reduce the flexibility of the disordered N-terminus and constrain its interaction with RRMs (Supplementary Fig. S9). Further experiments employing mutant U2AF2 should figure out the impact of phosphorylation and proline conformation on the affinity for RNA.

### Approaching the new functions of U2AF

Expression of U2AF genes *in situ* shows distinctive patterns in *F. borealis*, which may reflect developmental functions (Supplementary Fig. S3). Divergent paralogs are expressed in the germline, while the conserved paralogs are preferentially expressed in tissues like the tail muscle and gut cells. Spatial transcriptomics could reveal whether cell types process certain classes of introns differently, and whether U2AF paralog expression is linked to AS. Addressing spliceosome functions directly in *F. borealis* represents a challenging task, and in the short term, we believe that heterologous systems will remain instrumental for gaining mechanistic information about non-canonical splicing.

By showing that U2AF2β binds AHCYL1 in mammalian cells, we revealed an unexpected link between splicing factors and regulators of the SAM metabolism. The protein sequence of AHCYL1 is remarkably well-conserved in tunicate genomes, which are otherwise characterized by frequent gene loss and remarkably high evolution rates [45]. It suggests a strong selective pressure for preserving AHCYL1 functions, and future studies should test the roles played during animal development and gene expression.

### Impact of m6A re-patterning for the recognition of 5′ss

**Figure 10.**
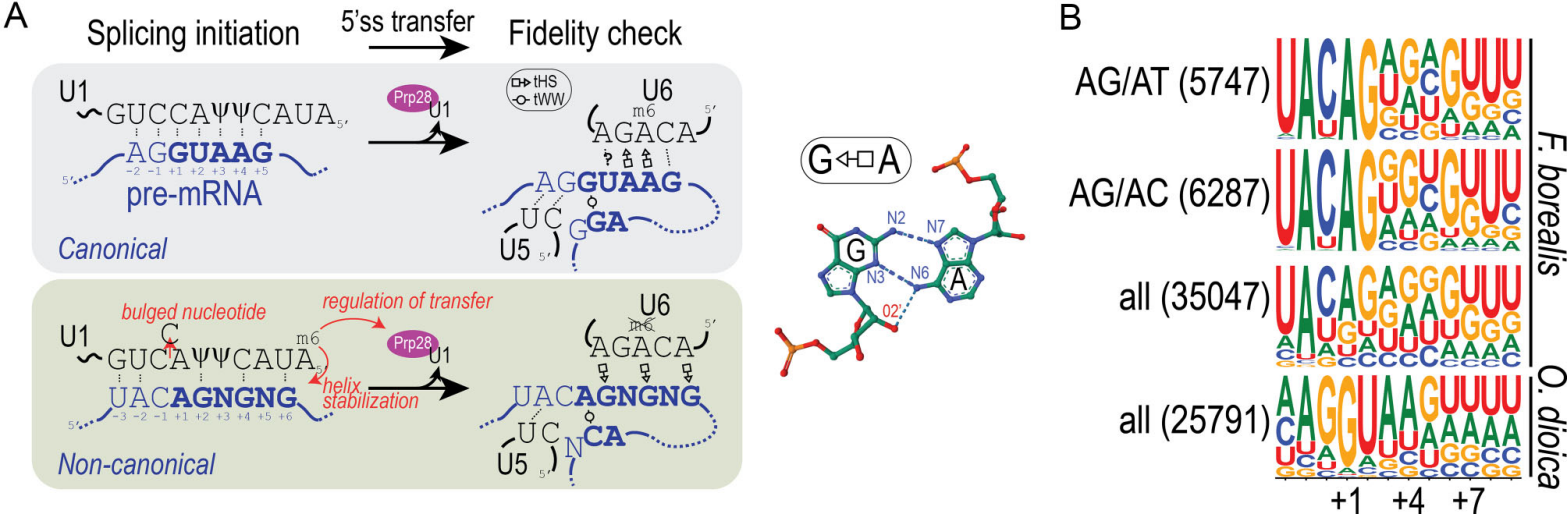
RNA interactions involved in 5′ss recognition. (**A**) Top panel represents base-pair interactions [10] between the 5′ss (intronic residues shown in bold) and U1 snRNA during the initiation of splicing, and between the 5′ss and U6 snRNA, after pre-mRNA transfer. The assembly of a network of RNA interactions between snRNA U5, U6, and the pre-mRNA provides a checkpoint for splicing accuracy. Dashed lines represent Watson–Crick interactions, symbols represent non-Watson–Crick pairs. Bottom panel proposes a model of interactions with a non-canonical 5′ss representative of *F. borealis* introns. The 3D model shows an example of tHS interaction between the sugar edge of G_2657_ and the Hoogsteen edge of A_2664_ in the sarcin/ricin domain from *E. coli* 23S rRNA (PDB 3DVZ) [58]. (**B**) Sequence logos of 5′ss showing overrepresentation of guanidine in *F. borealis* introns.

In the absence of a strong complementarity with non-canonical 5′ss of *F. borealis*, it remains difficult to predict whether the 5′ end of U1 snRNA anneals to the same position on the pre-mRNA or it would instead use a shifted register, as shown by others [46]. Because it has a stabilizing effect on base stacking [47], unpaired m6A_1_ at duplex end could compensate for mismatched positions between U1 snRNA and non-canonical 5′ss (Fig. 10A). The presence of m6A at this position could also impact 5′ss transfer from U1 snRNP to the U4/U6.U5 tri-snRNP. During this step, Prp28 unwinds the 5′ end of U1 snRNA, promoting 5′ss accessibility to the _41_ACAGAGA_47_ box of U6 snRNA [48].

While the role of m6A in U1 remains uncertain, we found a compelling link between the loss of m6A in U6 and the composition of *F. borealis* 5′ss. Compared to other species, adenines are less frequent beyond the position +2 of introns and we see instead an over-representation of guanines. We propose that methylation loss on A_43_ of U6 snRNA is instrumental for recognizing the composition bias in non-canonical 5′ss. When interacting with the eukaryotic 5′ss consensus, m6A_43_ establishes a non-Watson–Crick base pair with the adenine at position +4 of the intron under the *trans* Hoogsteen/Sugar geometry (tHS) [49–51] (Fig. 10A). Reducing m6A levels in the ACAGAGA box [50] favors splicing when a uridine is present at position +4, suggesting that the 5′ss/U6 duplex can accommodate *trans* Watson–Crick/Watson–Crick (tWW) interactions without impairing splicing. High-resolution structures of the spliceosome assembled on various pre-mRNA substrates also show that the ACAGAGA box can interact with a variety of sequences by mobilizing a mixture of *trans* base pair geometries [52, 53]. By aligning the U6 snRNA on the 5′ss consensus of two main classes of *F. borealis* introns, we predict that tHS interactions can be established between adenines of the ACAGAGA box and intronic guanines at positions +2, +4, and +6. In this model, methylation loss on A_43_ could play a critical role in stabilizing tHS pairing, by enabling an additional hydrogen bond between the N6 amine of A_43_ and the sugar of the opposite guanine (Fig. 10B).

### Evolutionary and chemical constraints to the selectivity of the spliceosome

Nematodes and larvacean tunicates are the only metazoan phyla that have lost all snRNA components of the U12-type spliceosome, but it did not coincide with a significant reduction of non-GT/AG introns compared to other species [4, 7, 13]. Losing the U12-type equipment may reduce the capacity to process a larger variety of introns. Losing the extra flexibility provided by the U12-type spliceosome could amplify negative impacts on splicing efficiency that are caused by splice site divergence, which could originate from splice site erosion or from the activity of introner transposons carrying weak splice sites [4, 54]. A sustainable solution would be to reintroduce flexibility in the splicing machinery, through the gain of new components or the modification of existing ones.

Mutation of essential genes such as U2AF1 and U2AF2 can compromise cellular functions, and gaining extra copies also bears the risk of giving rise to negative dominants. Human ZRSR2 is a functional analog of U2AF1, whose loss has been linked to myelodysplastic syndrome [55]. Therefore, risks associated with functional redundancy could be outweighed by the benefits of removing introns that contribute to developmental disorders and under this perspective, the case of *F. borealis* U2AF can be viewed as a common example of neofunctionalization mediated by gene duplication. Considering the key role during splicing, U1 snRNA could have been likely to provide the genetic substrate for evolving molecules dedicated to non-canonical splicing. This was not the alternative employed during the evolution of *F. borealis*, whose two U1 snRNA versions possess identical 5′ ends [4]. Instead, implementing m6A_1_ in U1 snRNA could promote new interactions with non-canonical 5′ss, while preserving binding to the minority of canonical 5′ss. Duplication and evolution of divergent U1 snRNA could be constrained at other levels, for instance functions during pre-mRNA processing or the necessity of scaling snRNP expression for gaining a significant functional output. The presence of m6A on U1 snRNA has been examined only by few studies, and its impact on splicing remains unknown. Engaging in this direction will require novel experimental approaches, since current information based on FTO^−/−^ transcriptomes cannot permit to distinguish the effects caused by the presence of m6A_1_ on U1 snRNA [37] from those caused by the loss of m6A on pre-mRNA sequences [56].

Base modifications present on snRNAs modulate the interactions with the pre-mRNA [50], and we propose they can potentiate the inherent flexibility of the spliceosome, which is reflected in the large diversity of intron ends found in the *F. borealis* genome. Splicing depends on the conservation of non-Watson–Crick pairs between the first and the last base of the intron [9]. During splicing, G−G and A−C terminal pairs (respectively formed with GT/AG and AT/AC introns) adopt a tWW conformation [9, 57]. These pairs are isosteric, explaining why the U2-type spliceosome can process AT/AC introns in certain conditions. In *F. borealis*, the termini of at least 55% of introns could form tWW interactions isosteric or nearly isosteric to G−G, with isodiscrepancy indexes under 3.5 [51] (Supplementary Table S2). If we consider an alternative *cis* Watson−Crick/Hoogsteen conformation that is observed in the *S. cerevisiae* spliceosome [11], this proportion will correspond to 45% of *F. borealis* introns. Definitive answers will be provided by examining how modified bases contribute to organizing the placement of introns during the splicing reaction, and ultimately, by gaining experimental structures of the *F. borealis* spliceosome.

## Supplementary Material

gkag659_Supplemental_Files

## Data Availability

The NCBI BioProject (https://www.ncbi.nlm.nih.gov/bioproject) PRJNA1345077 includes the animal sequencing data generated in this study: Draft genome assemblies (accessions SAMN52661553 and SAMN52661559-60), PacBio full-length non-concatemer cDNA reads (accession SRR35789923-26), Illumina RNA-seq reads (accession SRR37993504-7), and transcripts produced with the Isoseq pipeline. Sequencing data produced from HEK293T cells transfected with U2AF expression constructs were deposited at ArrayExpress (https://www.ebi.ac.uk/biostudies/) under accessions E-MTAB-15845 (CLiP-seq experiments), E-MTAB-15864 (RNA-seq experiments), and E-MTAB-15870 (Long-read sequencing of cDNA amplicons). Illumina sequencing reads from RNAcompete assays were deposited at NCBI under BioProject (https://www.ncbi.nlm.nih.gov/bioproject) PRJNA1346918. The *F. pellucida* draft genome assembly is available under the NCBI BioProject (https://www.ncbi.nlm.nih.gov/bioproject) PRJNA1456366. Spectra files from MS runs are available at the Zenodo repository with DOI https://doi.org/10.5281/zenodo.20524812.

## References

[1] Roca X, Krainer AR, Eperon IC. Pick one, but be quick: 5' splice sites and the problems of too many choices. Genes Dev. 2013;27:129–44. 10.1101/gad.209759.112 23348838 PMC3566305

[2] Kielkopf CL, Rodionova NA, Green MR et al. A novel peptide recognition mode revealed by the X-ray structure of a core U2AF35/U2AF65 heterodimer. Cell. 2001;106:595–605. 10.1016/S0092-8674(01)00480-9 11551507

[3] Guminska N, Plecha M, Zakrys B et al. Order of removal of conventional and nonconventional introns from nuclear transcripts of *Euglena gracilis*. PLoS Genet. 2018;14:e1007761. 10.1371/journal.pgen.1007761 30365503 PMC6221363

[4] Henriet S, Colom Sanmarti B, Sumic S et al. Evolution of the U2 spliceosome for processing numerous and highly diverse non-canonical introns in the Chordate *Fritillaria borealis*. Curr Biol. 2019;29:3193–9.e3194. 31543449 10.1016/j.cub.2019.07.092

[5] Shao C, Yang B, Wu T et al. Mechanisms for U2AF to define 3' splice sites and regulate alternative splicing in the human genome. Nat Struct Mol Biol. 2014;21:997–1005. 10.1038/nsmb.2906 25326705 PMC4429597

[6] Yoshida H, Park SY, Oda T et al. A novel 3' splice site recognition by the two zinc fingers in the U2AF small subunit. Genes Dev. 2015;29:1649–60. 10.1101/gad.267104.115 26215567 PMC4536312

[7] Olthof AM, Schwoerer CF, Girardini KN et al. Taxonomy of introns and the evolution of minor introns. Nucleic Acids Res. 2024;52:9247–66. 10.1093/nar/gkae550 38943346 PMC11347168

[8] Erkelenz S, Theiss S, Kaisers W et al. Ranking noncanonical 5' splice site usage by genome-wide RNA-seq analysis and splicing reporter assays. Genome Res. 2018;28:1826–40. 10.1101/gr.235861.118 30355602 PMC6280755

[9] Parker R, Siliciano PG. Evidence for an essential non-Watson–Crick interaction between the first and last nucleotides of a nuclear pre-mRNA intron. Nature. 1993;361:660–2. 10.1038/361660a0 8437627

[10] Artemyeva-Isman OV, Porter ACG. U5 snRNA interactions with exons ensure splicing precision. Front Genet. 2021;12:676971. 10.3389/fgene.2021.676971 34276781 PMC8283771

[11] Bai R, Yan C, Wan R et al. Structure of the post-catalytic spliceosome from *Saccharomyces cerevisiae*. Cell. 2017;171:1589–1598 e1588. 29153833 10.1016/j.cell.2017.10.038

[12] Costa M, Walbott H, Monachello D et al. Crystal structures of a group II intron lariat primed for reverse splicing. Science. 2016;354:aaf9258. 27934709 10.1126/science.aaf9258

[13] Marz M, Kirsten T, Stadler PF. Evolution of spliceosomal snRNA genes in metazoan animals. J Mol Evol. 2008;67:594–607. 10.1007/s00239-008-9149-6 19030770

[14] Sales-Lee J, Perry DS, Bowser BA et al. Coupling of spliceosome complexity to intron diversity. Curr Biol. 2021;31:4898–4910.e4. 34555349 10.1016/j.cub.2021.09.004 PMC8967684

[15] Stark MR, Dunn EA, Dunn WS et al. Dramatically reduced spliceosome in *Cyanidioschyzon merolae*. Proc Natl Acad Sci USA. 2015;112:E1191–1200. 25733880 10.1073/pnas.1416879112 PMC4371933

[16] Henriet S, Aasjord A, Chourrout D. Laboratory study of *Fritillaria* lifecycle reveals key morphogenetic events leading to genus-specific anatomy. Front Zool. 2022;19:26. 10.1186/s12983-022-00471-y 36307829 PMC9617304

[17] Henriet S, Sumic S, Doufoundou-Guilengui C et al. Embryonic expression of endogenous retroviral RNAs in somatic tissues adjacent to the *Oikopleura* germline. Nucleic Acids Res. 2015;43:3701–11. 10.1093/nar/gkv169 25779047 PMC4402516

[18] Naville M, Henriet S, Warren I et al. Massive changes of genome size driven by expansions of non-autonomous transposable elements. Curr Biol. 2019;29:1161–1168.e6. 30880010 10.1016/j.cub.2019.01.080

[19] Zhou P, Lugovskoy AA, Wagner G. A solubility-enhancement tag (SET) for NMR studies of poorly behaving proteins. J Biomol NMR. 2001;20:11–4. 10.1023/A:1011258906244 11430750

[20] Tari M, Manceau V, de Matha Salone J et al. U2AF(65) assemblies drive sequence-specific splice site recognition. EMBO Rep. 2019;20:e47604. 31271494 10.15252/embr.201847604 PMC6681011

[21] Buchbender A, Mutter H, Sutandy FXR et al. Improved library preparation with the new iCLIP2 protocol. Methods. 2020;178:33–48. 10.1016/j.ymeth.2019.10.003 31610236

[22] Busch A, Bruggemann M, Ebersberger S et al. iCLIP data analysis: a complete pipeline from sequencing reads to RBP binding sites. Methods. 2020;178:49–62. 10.1016/j.ymeth.2019.11.008 31751605

[23] Krakau S, Richard H, Marsico A. PureCLIP: capturing target-specific protein-RNA interaction footprints from single-nucleotide CLIP-seq data. Genome Biol. 2017;18:240. 10.1186/s13059-017-1364-2 29284540 PMC5746957

[24] McIlwain S, Tamura K, Kertesz-Farkas A et al. Crux: rapid open source protein tandem mass spectrometry analysis. J Proteome Res. 2014;13:4488–91. 10.1021/pr500741y 25182276 PMC4184452

[25] Hong T, Yuan Y, Chen Z et al. Precise antibody-independent m6A identification via 4SedTTP-involved and FTO-assisted strategy at single-nucleotide resolution. J Am Chem Soc. 2018;140:5886–9. 10.1021/jacs.7b13633 29489347

[26] Ray D, Ha KCH, Nie K et al. RNAcompete methodology and application to determine sequence preferences of unconventional RNA-binding proteins. Methods. 2017;118-119:3–15. 10.1016/j.ymeth.2016.12.003 27956239 PMC5411283

[27] Olthof AM, Hyatt KC, Kanadia RN. Minor intron splicing revisited: identification of new minor intron-containing genes and tissue-dependent retention and alternative splicing of minor introns. BMC Genomics. 2019;20:686. 10.1186/s12864-019-6046-x 31470809 PMC6717393

[28] Clark F, Thanaraj TA. Categorization and characterization of transcript-confirmed constitutively and alternatively spliced introns and exons from human. Hum Mol Genet. 2002;11:451–64. 10.1093/hmg/11.4.451 11854178

[29] Martins de Araújo M, Bonnal S, Hastings ML et al. Differential 3' splice site recognition of SMN1 and SMN2 transcripts by U2AF and U2 snRNP. RNA. 2009;15:515–23. 10.1261/rna.1273209 19244360 PMC2661831

[30] Zarnack K, Konig J, Tajnik M et al. Direct competition between hnRNP C and U2AF65 protects the transcriptome from the exonization of *Alu* elements. Cell. 2013;152:453–66. 10.1016/j.cell.2012.12.023 23374342 PMC3629564

[31] Choudhary B, Norris A. Conserved role for spliceosomal component PRPF40A in microexon splicing. RNA. 2024;31:43–50. 39389624 10.1261/rna.080142.124 PMC11648925

[32] Budnik N, Leroux AE, Cooke M et al. The role of S-adenosylhomocysteine hydrolase-like 1 in cancer. Biochim Biophys Acta Mol Cell Res. 2024;1871:119819. 10.1016/j.bbamcr.2024.119819 39154900

[33] Pinto R, Vagbo CB, Jakobsson ME et al. The human methyltransferase ZCCHC4 catalyses N6-methyladenosine modification of 28S ribosomal RNA. Nucleic Acids Res. 2020;48:830–46. 10.1093/nar/gkz1147 31799605 PMC6954407

[34] Ishigami Y, Ohira T, Isokawa Y et al. A single m^6^A modification in U6 snRNA diversifies exon sequence at the 5' splice site. Nat Commun. 2021;12:3244. 10.1038/s41467-021-23457-6 34050143 PMC8163875

[35] Pendleton KE, Chen B, Liu K et al. The U6 snRNA m^6^A methyltransferase METTL16 regulates SAM synthetase intron retention. Cell. 2017;169:824–835 e814. 28525753 10.1016/j.cell.2017.05.003 PMC5502809

[36] Flamand MN, Tegowski M, Meyer KD. The proteins of mRNA modification: writers, readers, and erasers. Annu Rev Biochem. 2023;92:145–73. 10.1146/annurev-biochem-052521-035330 37068770 PMC10443600

[37] Mauer J, Sindelar M, Despic V et al. FTO controls reversible m^6^Am RNA methylation during snRNA biogenesis. Nat Chem Biol. 2019;15:340–7. 10.1038/s41589-019-0231-8 30778204 PMC6984009

[38] Krogh N, Kongsbak-Wismann M, Geisler C et al. Substoichiometric ribose methylations in spliceosomal snRNAs. Org Biomol Chem. 2017;15:8872–6. 10.1039/C7OB02317K 29048444

[39] Decombe A, Peersen O, Sutto-Ortiz P et al. Internal RNA 2'-O-methylation on the HIV-1 genome impairs reverse transcription. Nucleic Acids Res. 2024;52:1359–73. 10.1093/nar/gkad1134 38015463 PMC10853786

[40] Larue GE, Elias M, Roy SW. Expansion and transformation of the minor spliceosomal system in the slime mold *Physarum polycephalum*. Curr Biol. 2021;31:3125–3131.e4. 34015249 10.1016/j.cub.2021.04.050

[41] Hoskins AA, Moore MJ. The spliceosome: a flexible, reversible macromolecular machine. Trends Biochem Sci. 2012;37:179–88. 10.1016/j.tibs.2012.02.009 22480731 PMC3508674

[42] Voith von Voithenberg L, Sanchez-Rico C, Kang HS et al. Recognition of the 3' splice site RNA by the U2AF heterodimer involves a dynamic population shift. Proc Natl Acad Sci USA. 2016;113:E7169–75. 27799531 10.1073/pnas.1605873113 PMC5135374

[43] David CJ, Boyne AR, Millhouse SR et al. The RNA polymerase II C-terminal domain promotes splicing activation through recruitment of a U2AF65-Prp19 complex. Genes Dev. 2011;25:972–83. 10.1101/gad.2038011 21536736 PMC3084030

[44] Cho S, Hoang A, Sinha R et al. Interaction between the RNA binding domains of Ser–Arg splicing factor 1 and U1-70K snRNP protein determines early spliceosome assembly. Proc Natl Acad Sci USA. 2011;108:8233–8. 10.1073/pnas.1017700108 21536904 PMC3100968

[45] Denoeud F, Henriet S, Mungpakdee S et al. Plasticity of animal genome architecture unmasked by rapid evolution of a pelagic tunicate. Science. 2010;330:1381–5. 21097902 10.1126/science.1194167 PMC3760481

[46] Kramarek M, Soucek P, Reblova K et al. Splicing analysis of STAT3 tandem donor suggests non-canonical binding registers for U1 and U6 snRNAs. Nucleic Acids Res. 2024;52:5959–74. 10.1093/nar/gkae147 38426935 PMC11162779

[47] Roost C, Lynch SR, Batista PJ et al. Structure and thermodynamics of N6-methyladenosine in RNA: a spring-loaded base modification. J Am Chem Soc. 2015;137:2107–15. 10.1021/ja513080v 25611135 PMC4405242

[48] Charenton C, Wilkinson ME, Nagai K. Mechanism of 5' splice site transfer for human spliceosome activation. Science. 2019;364:362–7. 10.1126/science.aax3289 30975767 PMC6525098

[49] Galej WP, Nguyen TH, Newman AJ et al. Structural studies of the spliceosome: zooming into the heart of the machine. Curr Opin Struct Biol. 2014;25:57–66. 10.1016/j.sbi.2013.12.002 24480332 PMC4045393

[50] Shen A, Hencel K, Parker MT et al. U6 snRNA m6A modification is required for accurate and efficient splicing of *C. elegans* and human pre-mRNAs. Nucleic Acids Res. 2024;52:9139–60. 10.1093/nar/gkae447 38808663 PMC11347140

[51] Stombaugh J, Zirbel CL, Westhof E et al. Frequency and isostericity of RNA base pairs. Nucleic Acids Res. 2009;37:2294–312. 10.1093/nar/gkp011 19240142 PMC2673412

[52] Zhang Z, Kumar V, Dybkov O et al. Cryo-EM analyses of dimerized spliceosomes provide new insights into the functions of B complex proteins. EMBO J. 2024;43:1065–88. 10.1038/s44318-024-00052-1 38383864 PMC10943123

[53] Zhang Z, Kumar V, Dybkov O et al. Structural insights into the cross-exon to cross-intron spliceosome switch. Nature. 2024;630:1012–9. 10.1038/s41586-024-07458-1 38778104 PMC11208138

[54] Huff JT, Zilberman D, Roy SW. Mechanism for DNA transposons to generate introns on genomic scales. Nature. 2016;538:533–6. 10.1038/nature20110 27760113 PMC5684705

[55] Madan V, Kanojia D, Li J et al. Aberrant splicing of U12-type introns is the hallmark of ZRSR2 mutant myelodysplastic syndrome. Nat Commun. 2015;6:6042. 10.1038/ncomms7042 25586593 PMC4349895

[56] Nabeel-Shah S, Pu S, Burke GL et al. Recruitment of the m^6^A/m6Am demethylase FTO to target RNAs by the telomeric zinc finger protein ZBTB48. Genome Biol. 2024;25:246. 10.1186/s13059-024-03392-7 39300486 PMC11414060

[57] Deirdre A, Scadden J, Smith CW. Interactions between the terminal bases of mammalian introns are retained in inosine-containing pre-mRNAs. EMBO J. 1995;14:3236–46. 10.1002/j.1460-2075.1995.tb07326.x 7621835 PMC394385

[58] Olieric V, Rieder U, Lang K et al. A fast selenium derivatization strategy for crystallization and phasing of RNA structures. RNA. 2009;15:707–15. 10.1261/rna.1499309 19228585 PMC2661828

