# Supplementary material for: Deployment of non-canonical splicing in tunicate genomes is mediated by divergent U2AF function and changing m6A modification in U1 and U6 snRNA
Source: Nucleic Acids Res. 2026 Jul 2;54(12):gkag659. doi: 10.1093/nar/gkag659 (PMC13326754; doi:10.1093/nar/gkag659)
Supplement: gkag659_Supplemental_Files [file gkag659_supplemental_files.zip › NAR_revised_supplemental.pdf]

Supplemental data

The supplemental data includes:

Extended methods

Figures S1 to S15

Tables S1 to S3

Legends for Datasets S1, S2 and supplemental spreadsheets

Supplemental References

Extended methods

1: Reagents

We used the following kits for preparing Illumina and PacBio sequencing libraries from animal samples: Nextera XT DNA Library Preparation Kit (Illumina, FC-131-1024), NEBNext single-cell/low input RNA library preparation kit (New England Biolabs, E6420). We used NEBNext Small RNA library prep kit (New England Biolabs, E7560) for sequencing U2AF1-bound RNA oligos. Mammalian cells transcriptomes were prepared for Illumina sequencing with NEBNext Ultra II RNA Library Prep kit (New England Biolabs, E7770). Animal genomes were prepared for long-read sequencing using Ligation Sequencing Kit V14 (Oxford Nanopore Technology, SQK-LSK114). Total RNA was extracted from animal samples with Nucleospin RNAXS (Macherey-Nagel, 740902).

The following plasmids were used for constructing expression vectors: pRSFDuet-1 (Sigma, 71341), pSF-CMV-GST(NH2) (Sigma, OGS1147), pGEX-6P-1 (Sigma, GE28-9546-48) and pCDNA3.1/V5-His (Thermo, K480001). We used AHCYL1 CRISPR/Cas9 KO Plasmid (Santa Cruz Biotechnology, sc-417109) to inactivate AHCYL1 in mammalian cells. Protein immunoprecipitation was conducted with Anti-V5 magnetic beads (Sigma, SAE0203). We used 0.2 ml Strep-Tactin XT 4Flow columns (IBA, 2-5011-005) for U2AF purification.

We used the following antibodies to test protein expression: SAHH Antibody (Santa Cruz Biotechnology, sc-271389), AHCYL1/SAHH-3 Antibody (Santa Cruz Biotechnology, sc-271581), m6A antibody (ab151230, Abcam), V5 Tag Monoclonal Antibody, HRP (Thermo, R961-25). We performed ECL detection of Western blots using SuperSignal West Femto and Atto substrates (34094 and A38555, Thermo).

2: Biological resources

Specimens of *Fritillaria borealis* originate from laboratory populations maintained at the Michael Sars Centre[1]. Specimens of *Fritillaria haplostoma* and *Appendicularia sicula* were collected near Bergen[1, 2]. Specimens of *Fritillaria pellucida* were collected near La Jolla, (CA, USA)[2]. Cultures of HEK293T cells were established from frozen stocks [3] originally sourced from ATCC and were grown in complete DMEM supplemented with Fetal bovine Serum and penicillin/streptomycin.

We produced a cell culture defective for AHCYL1 expression by transfecting HEK293T cells with AHCYL1 CRISPR/Cas9 KO Plasmid (Santa Cruz), together with AHCYL1 HDR plasmid (Santa Cruz). Transfected cells were grown in complete DMEM for 72 hours, before replacing the media with complete DMEM supplemented with 4 $\mu g.ml^{-1}$ puromycin. Stable knock-out cells were selected with fresh DMEM-puromycin during two rounds lasting 72

hours each. Selected cells were subsequently grown in complete DMEM, and sub-cultured three times before assaying AHCYL1 expression and m6A levels.

3: Data Base Referencing

Sequence analyses included accessions retrieved from the Minor Intron Database[4], Uniprot[5], Ensembl[6] and Aniseed[7]. We used the STREME web served for motif discovery[8] and the WebLogo3 web server for producing sequence logos[9].

4: Statistical analyses

Dissociation constants were determined for up to two replicates with with the nlsfit function of the R package easynls, using an equation for a one-site model: $Kd= [P][R]/[PR]$ where P and R are free protein and RNA, and PR is the protein-RNA complex.

After long-read cDNA sequencing, isoform expression was scaled with: $E= (C - Mc)/Sc$ where E is the expression value, C is the isoform read count, Mc and Sc are the mean and standard deviation of read count. We classified unique splicing events for each sample, based on isoforms with $E > 0.75$. Sequence composition was examined with a non-redundant collection of unique introns. PPT scores were calculated with the rules established by Clark & Thanaraj[10].

For RNAcompete assays, we analyzed on an average 945.10^3 reads per library. We filtered out introns with low read count (no sample with >10 reads). Next, read counts were normalized against the total number of reads per sample, and for each intron, we measured enrichment using: $E=\log[(C1+2)/(C2+2)]$, where C1 and C2 correspond to read counts measured for different U2AF1 paralogues. We first selected introns with $E>0.12$ when comparing differential enrichment between either *F. borealis* U2AF1α and U2AF1β, or *O. dioica* U2AF1 and F. borealis U2AF1β. We scored the read count per intron, and we selected a group of top-scoring introns representing 80% of the total read count for sequence analysis. Composition statistics and motif detection were performed on RNA pool sequences clipped from their terminal dinucleotides

We used Salmon v1.10.3[11] to quantify gene expression (-p 30 --gcBias -l A) on three replicates of transfected cells, and we conducted Differential Gene Expression (DGE) analysis with R packages tximport v1.32.0 and DeSeq2 v1.44.0[12, 13]. Differentially expressed genes (DEGs) were filtered with a significance threshold of $-0.58 \leq$ Log2FoldChange value ≥ 0.58 and adjusted p value $padj < 0.05$. To examine enriched functional categories and pathways in DEGs, we performed GO and KEGG Over-representation analysis (ORA) using the clusterProfiler R package v4.12.6(19). We determined AS events with two-pass STAR genome-aligned bam files using the rMATS

turbo software v4.3.0 (--task both --nthread 30 --readLength 250 --variable-read-length --allow-clipping --novelSS --individual-counts -t paired) (20) with --novelSS function enabled to allow the detection of novel splice sites. The AS events detected were filtered using a significance threshold of FDR ≤ 0.05. We determined AS events with two-pass STAR genome-aligned bam files using the rMATS turbo software v4.3.0[14] with --novelSS function enabled to allow the detection of novel splice sites. The AS events detected were filtered using a significance threshold of FDR ≤ 0.05.

We used the $2^{-\Delta\Delta Cq}$ method for quantifying gene expression in RT-qPCR assays, using β-actin as a control.

For iCLiP assays, we used Pureclip[15] to call peaks corresponding to U2AF2 crosslinks, using Env crosslinks as an input control data and model parameters learning on chromosomes 1 to 4. Sequence analysis was conducted with a selection of unique U2AF2 peaks withs scores higher than 5.

5: Genome sequencing

We extracted high-molecular-weight genomic DNA from mature *F. borealis* gonads dissected under a microscope, with the Chomczynski procedure. The DNA corresponding to 190 individuals was pooled, and we used 1.5 µg for long-read sequencing with Oxford Nanopore Technologies (ONT) platforms. We prepared ONT libraries following the manufacturer's recommendations (Ligation sequencing DNA V14, ONT), but increasing incubation times for adapter ligation to one hour and bead elution time to 14 hours to improve DNA recovery. Between 250 and 300 ng of ONT library were sequenced on a MinION flow cell (R10.4.1). Available pores rapidly decreased during the run, presumably due to pore blocking by polysaccharide contaminants present in DNA preparations. To get enough coverage for genome assembly, we collected over 29.10^4 reads with N50=4.9 kb produced by sequencing three independent libraries.

The DNA from whole bodies of four mature *F. haplostoma* individuals was prepared as described above and prepared for Illumina sequencing (Nextera XT DNA Library Preparation Kit). These libraries were barcoded and pooled with *A. sicula* libraries prepared for an earlier study, using whole-genome amplified DNA (WGA-DNA) obtained from individual specimens [2]. Pooled libraries were sequenced with 150 bp PE on a 1.5B NovaSeq X flow cell at the Norwegian Sequencing Centre (NSC, Oslo, Norway), producing 56.10^7 and 87.10^6 reads for the *F. haplostoma* and the *A. sicula* libraries, respectively. We prepared ONT libraries for *A. sicula* using WGA-DNA produced from single individuals, as described above. We sequenced 400 ng of *A. sicula* ONT library on a PromethION 2 solo flow cell (R10.4.1) for 16.5 hours, producing 2.10^6 reads with reads N50=6.4 kb.

6: Transcriptome sequencing

For long-read RNA-seq, we extracted total RNA (Nucleospin RNA XS, Macherey-Nagel) from *F. borealis* (24 larvae representing three developmental stages, 3 immature and 8 mature adults), *F. haplostoma* (6 mature adults), and *A. sicula* specimens (one mature adult). We assessed RNA integrity during pilot experiments using RNA extracted from pooled animals. Extractions were performed on single animals, and the whole RNA sample was used for conversion to cDNA using NEBNext Single Cell/Low Input RNA Library Prep Kit (New England Biolabs, NEB). Individual transcriptomes were amplified with barcodes for a total of 17 cycles, following Pacbio's guidelines for isoform sequencing (Iso-Seq Express Template preparation). SMRTbell libraries were prepared from pools of barcoded cDNA and sequenced with two SMRT cells on a Sequel II instrument at the NSC. Circular Consensus Sequencing (CCS) reads and Full-Length Non-Concatemer reads were produced with the Iso-Seq pipeline (SMRT Link v10.2).

For short-read RNA-seq, we pooled total RNA extracted from the same *F. borealis* individuals mentioned above (17 larvae representing two developmental stages, 3 mature adults) to prepare four Illumina RNA-seq libraries using the NEBNext Single Cell/Low Input RNA Library Prep Kit. Barcoded libraries were pooled and sequenced 150 bp PE on a Novaseq 6000 SP flow cell at the NSC.

7: Genome assembly

We used long-read sequencing data to improve an existing assembly of the *F. borealis* genome produced with short-read data from a single individual[2]. We processed ONT reads with Guppy software v6.4.8 configured for GPU-accelerated basecalling with a superior accuracy model (dna_r10.4.1_e8.2_260bps_sup). We assembled ONT reads using Flye v2.9.4 (--nano-hq), and we merged long- and short-read assemblies together using Quickmerge v0.3. The hybrid assembly was polished with Illumina reads using Pilon v1.24, resulting in a final version with 4063 contigs, for a total size of 79.7 Mb and N50=39.9 kb.

To produce an *A. sicula* genome assembly, we used sequencing information obtained from WGA-DNA corresponding to a single individual. ONT reads were basecalled with Dorado V0.8.3, configured with a superior accuracy model. We assembled ONT reads with Flye v2.9.4 and polished the assembly with Illumina reads produced from the same individual. The final assembly contains 13.10^3 contigs, for a total size of 65 Mb and N50=6.9 kb.

We assembled 75.10^6 paired-end Illumina reads from the *F. haplostoma* samples with Megahit assembler [16] (-1). Heterozygous contigs were removed from the assembly with the Redundans pipeline (--minLength 1000), using the initial set of Illumina reads for

scaffolding and gap-closing steps. The final assembly contains 55.10^3 contigs, with a total size of 120 Mb and N50=2.2 kb.

8: Gene and intron annotations

Gene evidence and intron positions were obtained by aligning Pacbio transcripts to the genome assemblies. Only high-quality Pacbio isoforms were employed, corresponding to clusters produced by Full-Length Non-Concatemer (FLNC) reads that belong to the same barcode.

For *F. borealis*, we first used Cutadapt v1.16 to remove the splice leader sequence present at the beginning of transcripts (-g ACTCATCTCTAGAGTCTCAAGTCGAACTTACAG) [2]. We mapped a collection of 249123 trimmed transcripts on the genome assembly with minimap2, without an alignment guiding option that uses conservative positions flanking canonical splice sites (--splice-flank=no). We produced 34034 unique isoforms after collapsing transcripts with Python scripts (https://github.com/Magdoll/cDNA_Cupcake/blob/master/cupcake/tofu/collapse_isoforms_by_sam.py) [17]. After aligning transcripts to the genome, gap intervals were used to determine intron positions. We first collected 28845 introns whose borders could be aligned without ambiguity, corresponding to situations where the last position of the upstream exon and the first position of the downstream exon have different bases. In these non-ambiguous introns, we could detect 88% of the time an adenine at the penultimate position, consistent with the strong conservation observed in various intron classes of other eukaryotes [18, 19]. To annotate introns in the *F. haplostoma* and the *A. sicula* genomes, we used PacBio's Iso-Seq package to align transcripts over genome assemblies and to collapse redundant isoforms. As described above, we first established intron borders based on splice signals discovered with a set of non-ambiguous intron positions (1507 and 640 introns for *F. haplostoma* and *A. sicula*, respectively). To annotate introns in the *F. pellucida* genome, we first used BLASTX to find matches against a collection of 30987 open reading frames predicted from the *F. borealis* isoform collection. Intron borders were found based on gap intervals. We kept 450 non-ambiguous cases for which no gaps are present and amino-acid identity is strictly conserved in at least two codons out of five, in each flanking exon.

We produced a transcriptome reference by assembling genome coordinates that matched Pacbio Iso-seq transcripts, to which we aligned *F. borealis* Illumina RNA-seq reads using bowtie2 (--very-sensitive -N 1 -L 20 --mp 4,2 --score-min L,-0.6, -0.6). Illumina RNA-seq reads included those obtained with a Hiseq run during our previous study [2]. We excluded reads shorter than 151 bp (Novaseq run) or 101 bp (Hiseq run), and we used samtools to measure read depth and to extract reads that aligned to at least 20 bp on each side of exon

junctions. For junction enrichment, we measured local coverage over a 100 bp window centered on exon junctions and filtered out all cases with coverage under three. Junction enrichment was calculated by dividing read depth at the junction position by the average read depth 50 bp upstream and 50 bp downstream the junction.

9: Homology search and protein sequence analysis

Gene orthologs were annotated with reciprocal blast. First, we used human protein sequences as queries against transcriptomes and genome assemblies of the species of interest. We searched for orthologs in the annotated transcriptome and in the genome assembly of *O. dioica* (7), and for other larvaceans, we employed the genomic resources described in this study. Orthologs from other organisms were searched in the ENSEMBL and ANISEED (8) databases. Top hits were checked for protein length and domain conservation, then used as queries for reciprocal BLAST against the UNIPROT database to confirm annotation.

We used the PhyML/OneClick pipeline of NGphylogeny.fr [20] to build protein multiple sequences alignments (MSA) and phylogenies. Distance matrices were computed from MSA with the distmat tool from the EMBOSS package, using the Kimura protein distance algorithm for multiple substitution correction [21].

10: Expression and purification of U2AF heterodimers

The full-length open-reading frames (ORFs) of U2AF subunits were amplified from the cDNA of *F. borealis* with Taq polymerase, and A-tailed PCR products were cloned into the pGEM-T vector for sequencing. We employed a seamless cloning strategy (In-Fusion HD, Takara) to subclone U2AF ORFs into a bacterial expression vector. We first replaced positions 100-168 of MCS-1 in pRSFDUET-1 with the U2AF2 ORF in frame with the 6xHis tag. Then, we replaced the whole MCS-2 with the U2AF1 ORF, in frame with an N-terminal tag that consists of 30 amino acids of the Twin-Strep-tag, followed by amino acids 2-56 of the B1 domain of Streptococcal Protein G [22]. Deletion mutants were engineered with seamless cloning.

We transformed tandem expression constructs into *E. coli* BL21-AI. Clones were grown to OD=0.8 in terrific broth (TB) and protein expression was induced with 50 µM IPTG, in the presence of 0.4% L-arabinose and 0.1 M $ZnCl_2$ during 16 h at 15 °C. Cell pellets were lysed on ice in HHS buffer (HHS: Hepes 20 mM, NaCl 0.5 M, 10% glycerol, 1% Tween-20, pH 7.8) supplemented with 2 mg/mL lysozyme, 1 mM TCEP, 1 mM PMSF, and 1x Protease Inhibitor Cocktail (cOmplete, EDTA-free; Roche). Lysates were sonicated on ice three times with 10-second pulses, then incubated 30 minutes on ice with DNase I, 2U/mL RNase cocktail (Ambion), and 3% BioLock (IBA). Lysates were centrifuged at 18000 g for 30 minutes at

4 °C, and protein complexes assembled with U2AF1 were captured on a Strep-Tactin XT 4 flow gravity flow column (IBA). Column-bound complexes were washed with 20 column volumes (CV) of HHS, 5 CV of HLS buffer (HLS: Hepes 20 mM, NaCl 0.1 M, 5% glycerol, 0.5% Tween-20, pH 7.8), and 10 CV of HLS buffer supplemented with 5 mM biotin. Purified complexes were eluted six times with 1 CV of HLS supplemented with 0.1 M biotin. We pooled all eluted fractions and performed buffer exchange by centrifugation on 10 kDa MWCO columns (Zeba Spin, Thermo) equilibrated with HLS buffer.

11: Electrophoretic mobility shift assays

We used seamless cloning to insert the EMSA probe sequence in pUC19 downstream of a T7 promoter and immediately upstream of a SapI restriction site. Constructs were linearized with Sap I and used as templates for in vitro transcription (IVT) reactions (MEGAshortscript T7 transcription kit, Ambion). IVT products were treated with Turbo DNase (Ambion), purified with Phenol/Chloroform extraction, and treated with Calf Intestinal Alkaline Phosphatase (Thermo). Dephosphorylated probes were purified on RNA Clean & Concentrator-5 (RNA-CC5, Zymo Research). We end-labelled 100 ng of probe with T4 PNK (NEB) and 10 µCi [γ^{32}P]-ATP at 6000 Ci/mmol, and reaction products were purified on 8% PAGE/TBE-Urea 8M. For EMSA, 100 cpm probes were denatured for 2 minutes at 80 °C, placed on ice, and incubated 1 h on ice in EMSA buffer (HLS supplemented with 50 ng/µL BSA, 1% RNase inhibitor (Protector, Roche), 0.5 mM TCEP, 0.5 mM EDTA) in the presence of purified U2AF. RNPs were run on 6% PAGE/TBE 0.5x for 2 h at 4 °C under 5V/cm. Gels were fixed with a 10% EtOH/5% AcOH solution and dried under vacuum. We recorded signals with phosphor screens and a Typhoon FLA scanner (GE) and measured signal intensity with ImageJ. Dissociation constants were determined with the nlsfit function of the R package easynls, using an equation for a one-site model: $Kd= [P][R]/[PR]$ Where P and R are the free U2AF and probe, respectively, and PR is the U2AF-probe complex. Our conditions provide at least a 5-fold molar excess of U2AF compared to the probe.

12: RNA-compete assays

We designed a pool of 5633 unique sequences derived from *F. borealis* introns, which include the 20 last nucleotides of the introns, followed by 10 nucleotides of the downstream exon. The T7 promoter sequence, followed by an extra guanine residue, was added at the 5' end of the pool. A SapI restriction site was added at the 3' end, followed by a target sequence for PCR priming. After chemical synthesis (Twist Biosciences), we produced IVT templates from oligo pools with PCR reactions and primers specific to intron-flanking sequences. To preserve complexity and to avoid the formation of unspecific products, amplification rounds were performed with at least 60% of the initial pool as

template during a maximum of 6 PCR cycles. Template linearization, IVT reactions, and 5' phosphate removal were performed as described above. We checked the integrity of the RNA pool on 15%PAGE/TBE-Urea 8M.

We used seamless cloning to insert the full-length ORF of U2AF1 in pGEX-6P-1, in frame with the N-terminal GST. Constructs were transformed into *E. coli* BL21, selected clones were grown to OD=0.8 in TB and protein expression was induced for 16 h at 18 °C with 0.4 mM IPTG. Cell lysis and nucleic acid removal were performed as described above, in LY buffer (LYB: 25 mM Hepes, 0.35 M NaCl, 10% glycerol, 0.5% Tween-20, pH 7.5). After 10000 g centrifugation for 30 minutes at 4 °C, fusion proteins were captured on Glutathione-sepharose 4B (Cytiva) for 2 h at room temperature, washed with 30 bead volumes of LYB and eluted 4 times with 1.5 bead volumes of LYB supplemented with 50 mM glutathione. We exchanged the elution buffer to LYB as described above.

For RNA-binding assays, 2 picomoles of GST-U2AF1 were bound to 2 µL Glutathione High-Capacity Magnetic Agarose Beads (Millipore) for 1 h at room temperature in 0.2 mL of RB buffer (RBB: 25 mM Hepes, 0.5 M NaCl, 5% glycerol, 1 mM EDTA, 1 mM DTT, 2 mM $MgCl_2$, 0.5% Tween-20). After three washes with RBB to remove unbound proteins, we added 1 µM of RNA pool to the beads in a final volume of 0.5 mL RBB, together with 10 µg BSA, 4 µg Heparin, and 2 µL of RNase inhibitor. Samples were incubated for 2 h at 4 °C on a wheel, and beads were washed three times with 0.5 mL ice-cold RBB. Proteins were digested with Proteinase K in 10 mM Tris, 1% SDS, 2 mM EDTA for 15 minutes at 37 °C, and we recovered RNA with RNA-CC5. We prepared Illumina libraries using the NEBNext Small RNA library prep kit (NEB), and samples were sequenced on a MiSeq instrument at the NSC. After adapter trimming, an average of 84% reads could be mapped on the original collection of introns with Bowtie2 (--very-sensitive-local).

13: Long-read sequencing of mammalian cDNA amplicons

Cells transfected with U2AF1 and U2AF2 constructs were treated with 0.15 M cycloheximide for 8 h before harvest. Controls were treated with 0.1% DMSO. Cell pellets were washed in PBS, and RNA was extracted with TRIzol reagent (Invitrogen), following the manufacturer’s procedure. Samples were treated with Turbo DNase, and 1 µg RNA was converted to cDNA with Superscript IV reverse transcriptase, using a combination of random 10-mers and oligo-dT primers. For each target gene, we amplified cDNA with specific primers in separate PCR reactions, for a total of 20 cycles. PCR products from the same cDNA were pooled together and purified with AMPure XP SPRI beads (Beckman). We prepared libraries with barcodes to separate cDNA samples, following ONT’s procedure for barcoding and sequencing amplicons (SQK-LSK114). We sequenced 55 ng of library pool on a PromethION 2 solo flow cell (R10.4.1) for 2 h, producing 6.10^6 reads with N50=3.4

kb. Reads over 500 bp with a quality score over Q9 were basecalled with Dorado V0.8.3, configured with a high accuracy model. We trimmed adapters and primers with porechop, and processed the reads with TALON v6.0 [23], with a database corresponding to regions of the human genome targeted by the gene-specific primers. For each sample, we considered only isoforms whose sequencing coverage across samples is superior to 50, and we found splicing events based on exon positions mapped by TALON. Introns were classed as canonical (ending with GT/AG) or non-canonical (other combinations of terminal dinucleotides). Isoforms were classed as canonical unless their splicing involved at least one non-canonical splice site.

For measuring isoform diversity of target genes, we scaled expression across samples with: $E = (C - Mc)/Sc$ Where E is the scaled isoform expression value in the sample, C is the isoform read count in the sample, Mc is the mean of the isoform read count across samples, and Sc is the standard deviation of the isoform read count across samples. To score the splicing of intron classes, we first normalized each isoform read count over the total read count of transcripts from the same gene. We then established a score for each transfection by averaging the normalized read count of all isoforms produced by splicing the same intron class, divided by the average expression of these isoforms across all experimental conditions.

14: Transcriptome analysis of U2AF2-expressing cells

We used Trizol to extract total RNA from HEK293T cells transfected with either U2AF2 constructs described above, or with the pCDNA3.1 vector containing the *env* gene of an LTR retrotransposon [3]. We enriched mRNA from 10 µg of total RNA with the Dynabeads mRNA Purification kit (Ambion), and barcoded sequencing libraries were prepared from 50 ng poly-A^+ RNA with the NEBNext Ultra II RNA Library Prep kit (NEB). Pooled libraries were sequenced 150 bp PE on a 1.5B NovaSeqXPlus flow cell at the NSC.

Basic trimming of raw reads was performed with FastP v0.24.0 (-w 16 -q 20 -c -g --cut_front --cut_right) to remove adapter sequences and keep Q20 reads [24]. The data was mapped with STAR v2.5.2b (--outSAMunmapped Within --runThreadN 30 --outSAMtype BAM Unsorted --quantMode TranscriptomeSAM GeneCounts --quantTranscriptomeBan Singleend --outFilterType BySJout --outFilterMultimapNmax 20 --alignSJoverhangMin 8 --alignSJDBoverhangMin 1 --outFilterMismatchNmax 999 --outFilterMismatchNoverReadLmax 0.04 --alignIntronMin 20 --alignIntronMax 1000000 --alignMatesGapMax 1000000 --outStd BAM_Unsorted BAM Quant --readFilesCommand zcat) [25] to *H. sapiens* GRCh38.p14 primary genome assembly using the latest gene annotation file retrieved from GENCODE [26]. The mapping results were used to run an additional STAR two-pass mapping (--outSAMunmapped Within --runThreadN 30 --

outSAMtype BAM Unsorted --quantMode TranscriptomeSAM GeneCounts --quantTranscriptomeBan Singleend --outFilterType BySJout --outFilterMultimapNmax 20 --alignSJoverhangMin 8 --alignSJDBoverhangMin 1 --outFilterMismatchNmax 999 --outFilterMismatchNoverReadLmax 0.04 --alignIntronMin 20 --alignIntronMax 1000000 --alignMatesGapMax 1000000 --outStd BAM_Unsorted BAM Quant --readFilesCommand zcat –sjdbFileChrStartEnd) for further analysis of AS events.

For measuring the expression of endogenous U2AF in transfected cells, total RNA was first treated with Turbo DNAse before extraction with Phenol/Chloroform. We converted 250 ng of RNA to cDNA using Superscript IV reverse transcriptase (Invitrogen), and used 0.5% cDNA to conduct qPCR on a CFX opus 96 thermocycler with the IQ SYBR Green supermix (Bio-Rad).

15: Crosslinking and Immunoprecipitation followed by Sequencing

We followed the iCLiP2 procedure [27] to isolate RNA bound to tagged U2AF2 paralogs expressed in HEK293T cells after transfection. Two days post-transfection, cells were washed with PBS and irradiated on ice with 254 nm light (150 mJ). Cells were resuspended in lysis buffer supplemented with 1 mM PMSF and protease inhibitors (cOmplete-EDTA, Roche), before sonication on ice twice with 10-second pulses. Transcripts were fragmented for 3 minutes at 37 °C in the presence of 1 U/mL TURBO DNase and 20-1000 U/mL RNase I (Ambion). Insoluble material was pelleted by 10 minutes of centrifugation at 22000 g at 4 °C, and supernatant was added to Anti-V5 magnetic beads (Sigma, equivalent to 70 µL suspension), prewashed in lysis buffer. After binding 150 minutes on a wheel at 4 °C, RNPs were washed twice with ice-cold High-salt wash buffer and three times with ice-cold PNK buffer. RNA fragments were prepared for adapter ligation and purified as described previously [27]. Barcoded libraries corresponding to U2AF2- or Env-bound RNA were pooled and sequenced on NextSeq 150 bp PE at the NSC. Reads were trimmed and demultiplexed with Flexbar [28], mapped to the *H. sapiens* GRCh38.p14 primary genome assembly with Bowtie2 (-- very-sensitive-local), and PCR duplicates were removed with UMI-tools [29].

16: Identification of U2AF2 partners

Cells transfected with either U2AF constructs or a control containing the env gene were washed in PBS and lysed in HLS, supplemented with 1 mM TCEP, 1 mM PMSF, and protease inhibitors (cOmplete, Roche). Protein concentration was determined with bicinchoninic acid assay, and for each transfection, a sample equivalent to 6 µg protein was used for immunoprecipitation. Samples diluted in 1 mL HLS were first pre-cleared by incubation with Anti-HA magnetic beads (Sigma, equivalent to 70 µL suspension) for 1 h at

4 °C on a wheel. The supernatant was transferred to Anti-V5 magnetic beads (Sigma, equivalent to 70 µL suspension) and incubated for 2 h at 4 °C on a wheel. Beads were washed five times with 1.5 mL ice-cold HLS and resuspended in Laemmli buffer. Bound proteins were eluted for 5 min at 70 °C and separated on Bis-Tris SDS-8% PAGE. After Coomassie staining, bands between 37 and 100 kDa were excised. Peptides were digested in-gel and analyzed on an Orbitrap Eclipse Mass Spectrometer at the Proteomics Unit of the University of Bergen. After peptide identification, we established a database that include all proteins identified in the samples, and without common contaminants such as keratins. For each sample, we used the Crux pipeline [30] to determine protein abundance with a semi-quantitative approach (--threshold 0.01 --missed-cleavages 2 --precursor-window 10 --mods-spec 1M+15.99491 --num-decoys-per-target 5 --score-function xcorr --spectral-counting-fdr 0.01). We used the Normalized Spectral Abundance Factor (NSAF) computed for each protein of the database to score its relative abundance across IPs. For validation of protein interactions, samples separated on gel were transferred to a PVDF membrane and tested with either SAHH Antibody (sc-271389, Santa Cruz Biotechnology) or AHCYL1/SAHH-3 Antibody (sc-271581, Santa Cruz Biotechnology).

17: Detection of N6-methyladenosine in snRNA

Total RNA was extracted with either Trizol or miRVana miRNA isolation kit (Ambion), following the manufacturer's recommendations. To examine m6A levels, RNA was separated on 10% PAGE-Urea and transferred to a positively charged nylon membrane (Hybond N^+, Amersham). We detected N6-methyladenosine using a m6A antibody (ab151230, Abcam). To quantify total snRNA amounts, we performed Northern Blot using a pool of radioactive, end-labeled oligoribonucleotide probes complementary to U1, U2, U4, U5, and U6. Membranes were hybridized to probes in ULTRAhyb-Oligo (Ambion) during 14 h at 42 °C, then washed twice at 42 °C with 2x SSC / 0.5 % SDS. Signals were recorded and quantified as described above.

18: Mapping of m6A in U1 snRNA

We designed primer extension assays based on procedures described by Hong *et al.* [31]. Briefly, 1.5 µg total RNA was annealed to 150000 cpm of gel-purified DNA primer complementary to nucleotides 13-33 of U1 snRNA and 5' end-labelled with [γ^{32}P]-ATP. After 2 minutes at 90 °C, the hybridization mix was placed for 2 minutes at 50 °C, and 10 U Superscript IV RT (Invitrogen) was added in RT buffer (50 mM Tris-HCl, 4 mM $MgCl_2$, 10 mM DTT, 50 mM KCl, 30 µM dATP, 30 µM dGTP, 30 µM dCTP, pH 8.3) in the presence of 30 µM of either dTTP or 4SedTTP (Jena Biosciences). After 20 minutes of incubation at 50 °C, cDNA synthesis was stopped by the addition of Urea gel loading buffer, and denatured elongation

products were resolved on 15-20% PAGE-Urea sequencing gels. Signal was recorded on fixed gels and processed as described above.

Figures

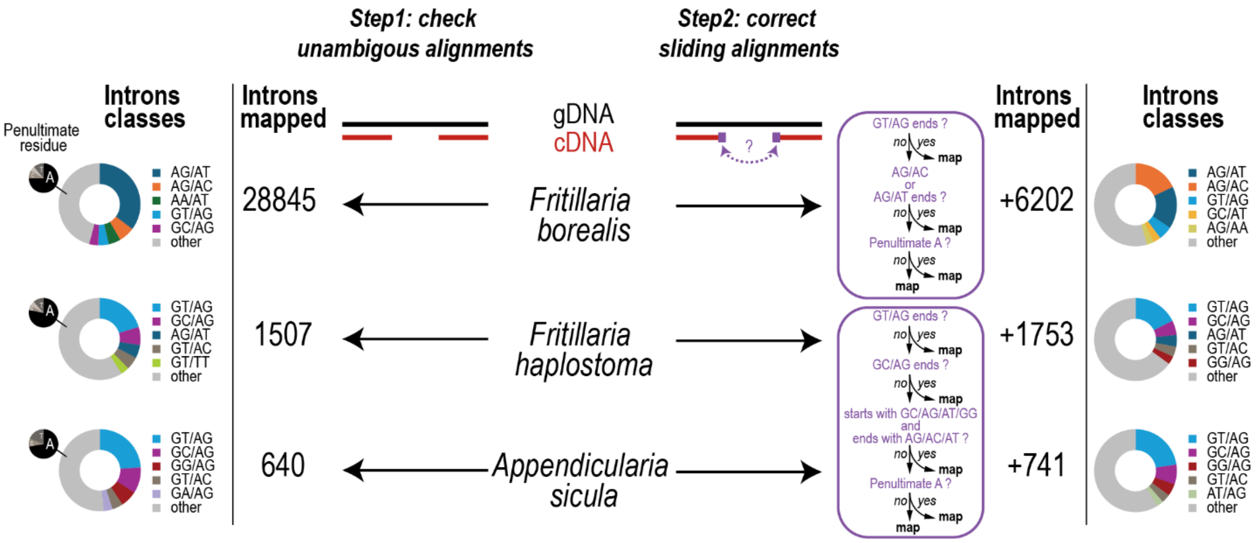

<u>Fig. S1</u>: Procedure for mapping intron borders in larvacean Fritillaridae genomes. We map introns from unambiguous gaps in alignments between cDNA and gDNA. This first step allows us to establish consensus rules to guide the correction of sliding gaps.

PUF60

	H. sapiens	B. lanceolatum	S. purpuratus	C. intestinalis	O. dioica	A. sicula	F. haplostoma	F. borealis
H. sapiens	0	19,75	39,44	31,93	83,06	58,52	55,29	61,87
B. lanceolatum		0	34,36	43,48	99,75	53,72	53,72	56,89
S. purpuratus			0	58,52	123,54	69	65,36	78,81
C. intestinalis				0	99,75	65,36	65,36	74,75
O. dioica					0	130,58	120,21	120,21
A. sicula						0	8,34	16,73
F. haplostoma							0	18,73
F. borealis								0

U1.70K

	H. sapiens	B. lanceolatum	S. purpuratus	C. intestinalis	O. dioica	A. sicula	F. haplostoma	F. borealis
H. sapiens	0	46,39	42,23	42,23	69,5	91,57	84,69	79,85
B. lanceolatum		0	51,89	54,19	86,36	88,06	91,57	88,06
S. purpuratus			0	53,03	69,5	97,1	89,8	79,85
C. intestinalis				0	64,09	83,04	84,69	83,04
O. dioica					0	91,57	95,22	86,36
A. sicula						0	46,39	45,33
F. haplostoma							0	54,19
F. borealis								0

CAPERα

	H. sapiens	B. lanceolatum	S. purpuratus	C. intestinalis	O. dioica	A. sicula	F. haplostoma	F. borealis
H. sapiens	0	27,85	32,64	40,96	74,02	62	60,61	64,86
B. lanceolatum		0	33,64	38,8	86,09	69,32	60,61	75,65
S. purpuratus			0	43,18	80,72	84,26	72,43	80,72
C. intestinalis				0	86,09	89,85	72,43	86,09
O. dioica					0	106,72	89,85	91,8
A. sicula						0	46,63	44,32
F. haplostoma							0	50,22
F. borealis								0

U1.A

	H. sapiens	B. lanceolatum	S. purpuratus	C. intestinalis	O. dioica	A. sicula	F. haplostoma	F. borealis
H. sapiens	0	18,55	25,89	26,67	63,29	68,26	85,1	65,74
B. lanceolatum		0	21,41	25,13	66,99	63,29	85,1	64,51
S. purpuratus			0	29,84	69,55	64,51	88,25	63,29
C. intestinalis				0	70,85	70,85	89,86	65,74
O. dioica					0	102,06	109,84	94,89
A. sicula						0	94,89	42,04
F. haplostoma							0	93,18
F. borealis								0

SF3b14

	H. sapiens	B. lanceolatum	S. purpuratus	C. intestinalis	O. dioica	A. sicula	F. haplostoma	F. borealis
H. sapiens	0	14,51	22,37	20,04	36,56	45,49	45,49	42,41
B. lanceolatum		0	15,58	14,51	32,44	45,49	45,49	48,7
S. purpuratus			0	15,58	36,56	47,08	45,49	45,49
C. intestinalis				0	37,98	43,94	39,43	42,41
O. dioica					0	43,94	52,03	53,74
A. sicula						0	18,9	20,04
F. haplostoma							0	22,37
F. borealis								0

U1.C

	H. sapiens	B. lanceolatum	S. purpuratus	C. intestinalis	O. dioica	F. haplostoma	F. borealis
H. sapiens	0	14,27	7,81	14,27	47,26	50,72	43,94
B. lanceolatum		0	7,81	16,55	43,94	50,72	50,72
S. purpuratus			0	16,55	40,74	43,94	43,94
C. intestinalis				0	43,94	47,26	43,94
O. dioica					0	37,67	58,09
F. haplostoma						0	29,08
F. borealis							0

SF3b155

	H. sapiens	B. lanceolatum	S. purpuratus	C. intestinalis	O. dioica	F. haplostoma	F. borealis
H. sapiens	0	7,38	9,82	11,17	20,68	21,12	25,68
B. lanceolatum		0	8,68	11,17	19,15	22,23	25,44
S. purpuratus			0	13,54	21,56	23,59	25,91
C. intestinalis				0	20,46	23,36	25,44
O. dioica					0	28,06	27,82
F. haplostoma						0	18,08
F. borealis							0

U2AF2

	H. sapiens (L4)	B. lanceolatum	S. purpuratus	C. intestinalis	O. dioica	A. sicula	F. haplostoma	F. borealis (α)	F. borealis (β)
H. sapiens	0	24,05	24,57	23,53	38,96	33,48	31,74	35,87	59,57
B. lanceolatum		0	26,71	31,74	40,87	40,23	40,87	42,17	69,8
S. purpuratus			0	28,35	38,34	37,71	35,27	36,48	66,25
C. intestinalis				0	35,27	31,16	32,9	35,87	63,68
O. dioica					0	38,34	39,59	35,87	68,01
A. sicula						0	18,49	19,47	45,51
F. haplostoma							0	18,98	46,2
F. borealis (α)								0	52,62
F. borealis (β)									0

U2AF1

	H. sapiens	H. sapiens (L4)	B. lanceolatum	S. purpuratus	C. intestinalis	O. dioica	A. sicula	F. haplostoma	F. borealis (α)	F. borealis (β)
H. sapiens	0	14,24	5,5	9,41	12,13	24,94	28,28	25,76	24,13	47,46
H. sapiens (L4)		0	14,24	19,41	19,41	30,89	35,42	35,42	30,01	61,51
B. lanceolatum			0	9,41	10,08	24,94	26,59	23,32	20,95	44,28
S. purpuratus				0	14,24	25,76	30,01	26,59	24,94	48,54
C. intestinalis					0	27,44	29,14	26,59	24,94	53,03
O. dioica						0	36,36	35,42	34,5	65,41
A. sicula							0	17,15	15,68	43,25
F. haplostoma								0	16,41	41,22
F. borealis (α)									0	43,25
F. borealis (β)										0

ZRSR2

	H. sapiens	B. lanceolatum	S. purpuratus	C. intestinalis
H. sapiens	0	62,39	70,47	115,85
B. lanceolatum		0	67,97	117,26
S. purpuratus			0	121,63
C. intestinalis				0

0 20 40 60 80 100 120 150

Kimura protein distance

<u>Fig. S2</u>: Distance matrices produced from multiple sequence alignments of proteins involved in splice site recognitions.

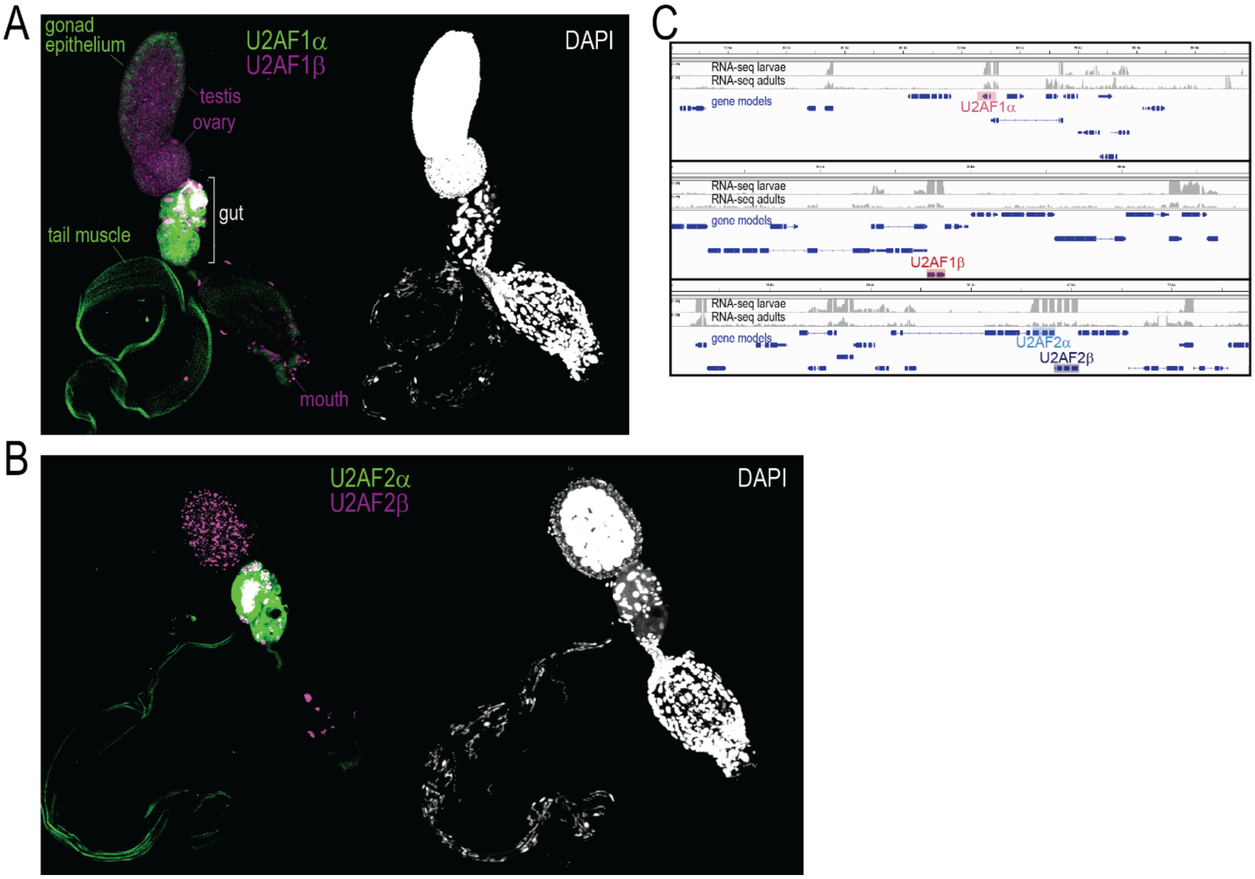

<u>Fig. S3:</u> Expression of U2AF paralogues in *F. borealis*. **A)** Whole-mount HCR *in situ* hybridization performed on maturing adults, with fluorescent probes targeting either U2AF1α (green) or U2AF1β (magenta). Nuclei were simultaneously stained with DAPI (white). **B)** Same as **A)**, with fluorescent probes targeting either U2AF2α (green) or U2AF2β (magenta). **C)** Genome browser screenshots showing the gene models for U2AF1 and U2AF2 paralogues, and the corresponding RNA-seq coverage during early or late developmental stages.

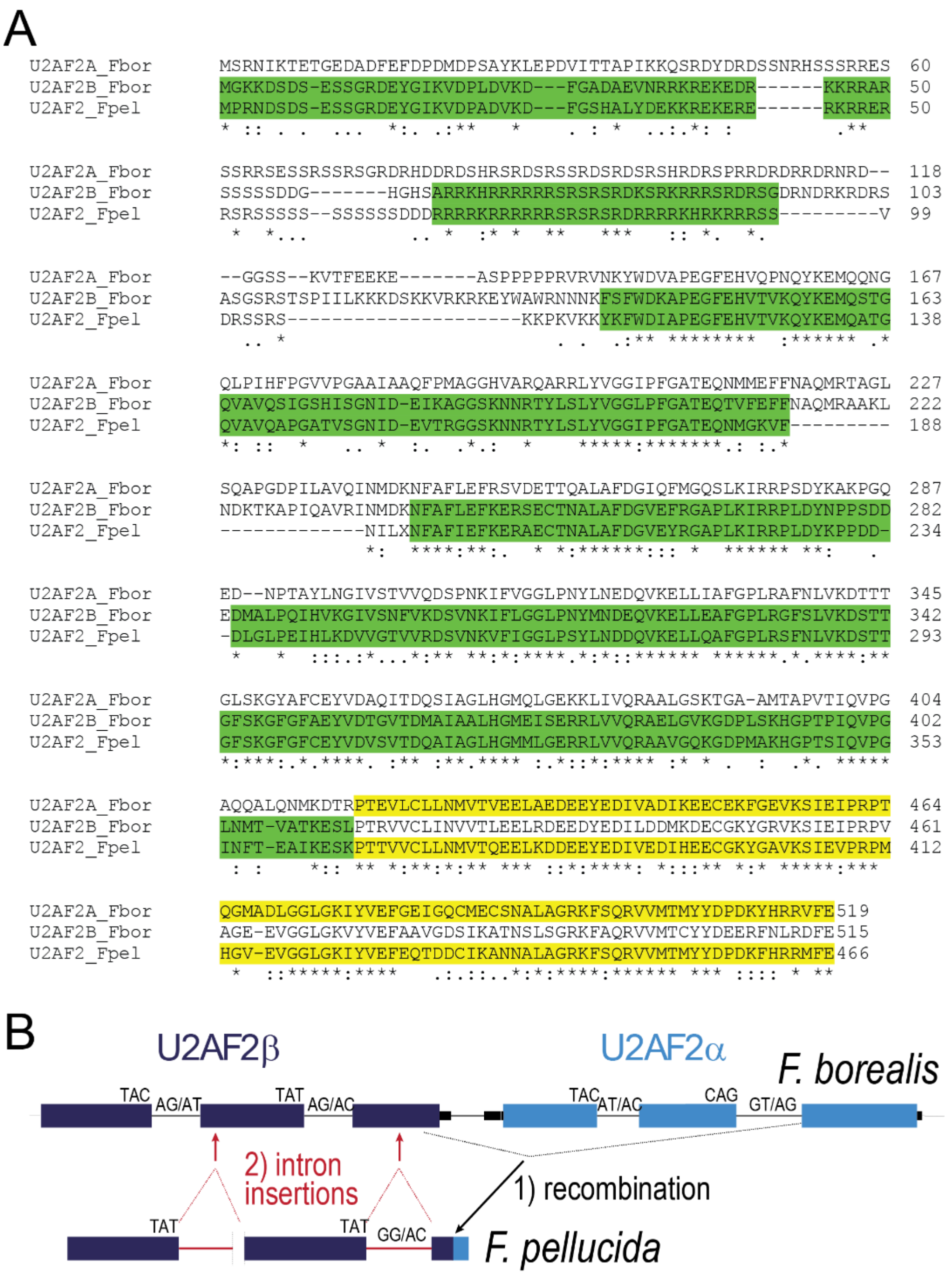

Fig. S4: Evolution of U2AF2 in *F. pellucida*. **A)** Multiple sequence alignment of *F. borealis* U2AF2 paralogues with the *F. pellucida* U2AF2. Color highlight shows the strongest similarity between *F. pellucida* U2AF2 and *F. borealis* U2AF2α (yellow) or U2AF2β (green). **B)** Duplicates of U2AF2 form a gene cluster in *F. borealis*. The *F. pellucida* U2AF2 gene has features of U2AF2α and U2AF2β, indicating a secondary reduction of the cluster by recombination between duplicates, followed by rearrangements of intron positions.

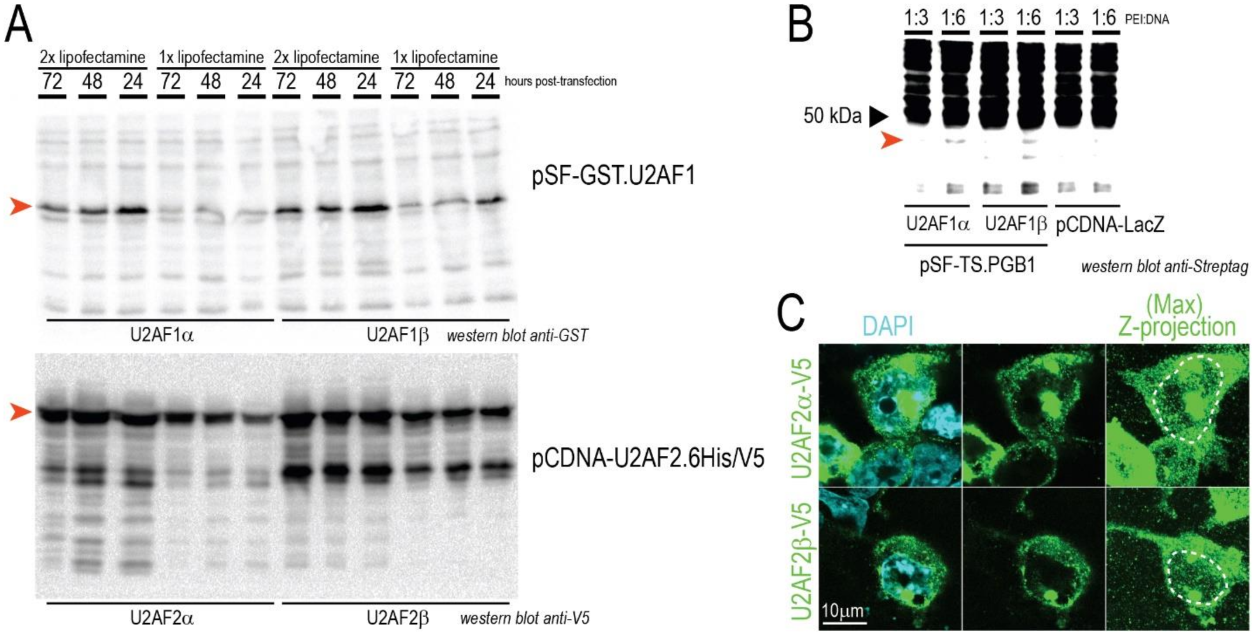

<u>Fig. S5:</u> Expression of *F. borealis* U2AF2 constructs in HEK293T cells. **A)** Lipofectamine transfections. Cells were transfected with two ratios of lipofectamine and DNA constructs, that carry either U2AF1 (top) or U2AF2 (bottom) paralogs α and β in fusion with protein tags. Cells were harvested after 1 to 3 days post-transfection, and protein expression was monitored with western blot against protein tags. Arrowheads mark the band corresponding to tagged U2AF. **B)** Polyethylenimine (PEI) transfections. Cells were transfected with two ratios of PEI and DNA construct carrying either U2AF1paralogs α and β fused to a streptavidin tag, or an untagged lacZ gene. Arrowheads mark the band corresponding to tagged U2AF, detected with a western blot against the protein tag. **C)** Immunofluorescence confocal microscopy of cells transfected with pCDNA-U2AF2.6His/V5 constructs. Tagged proteins were detected with a primary anti-V5 antibody coupled to fluorescein. Dashed lines mark the contour of nuclei.

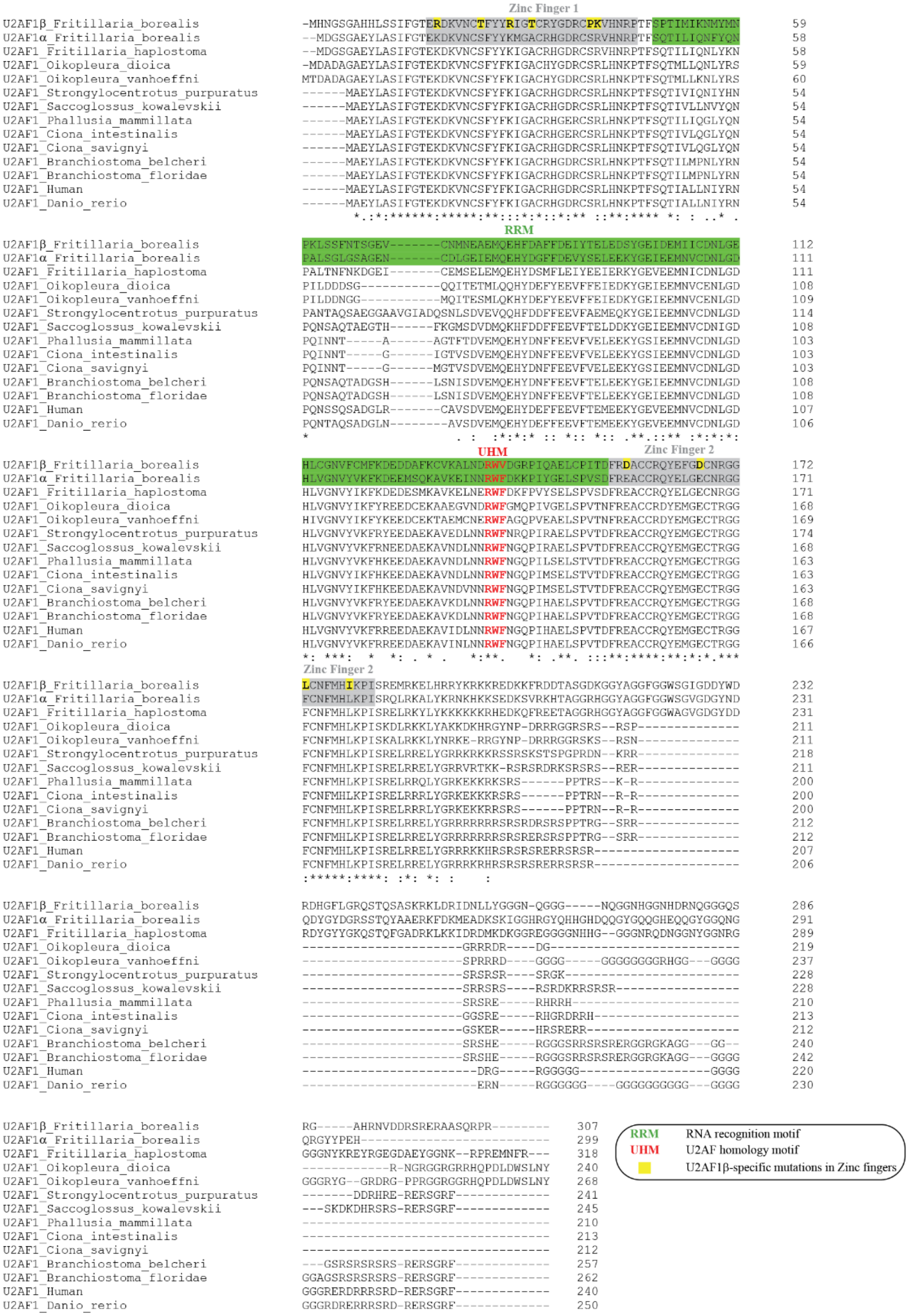

Fig. S6: Multiple sequence alignment of *F. borealis* U2AF1 paralogues with deuterostome U2AF1.

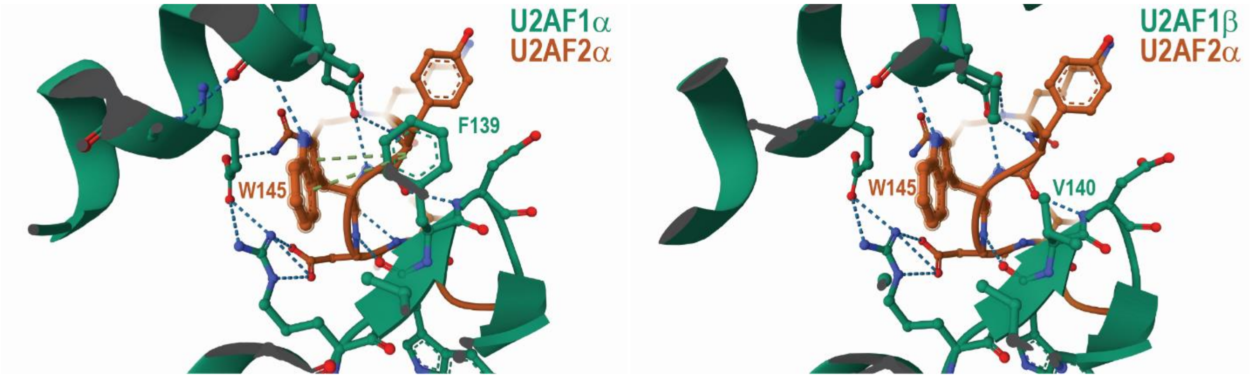

<u>Fig. S7:</u> Models of interactions established between F. borealis U2AF2α and U2AF1α (top) or U2AF1β (bottom), showing the UHM/ULM interface. Interactions between aromatic rings are shown with dashed green lines, dashed blue lines represent hydrogen interactions. We predicted a set of structures with AlphaFold 3[32] and kept those aligning best with the crystal structure of human U2AF[33].

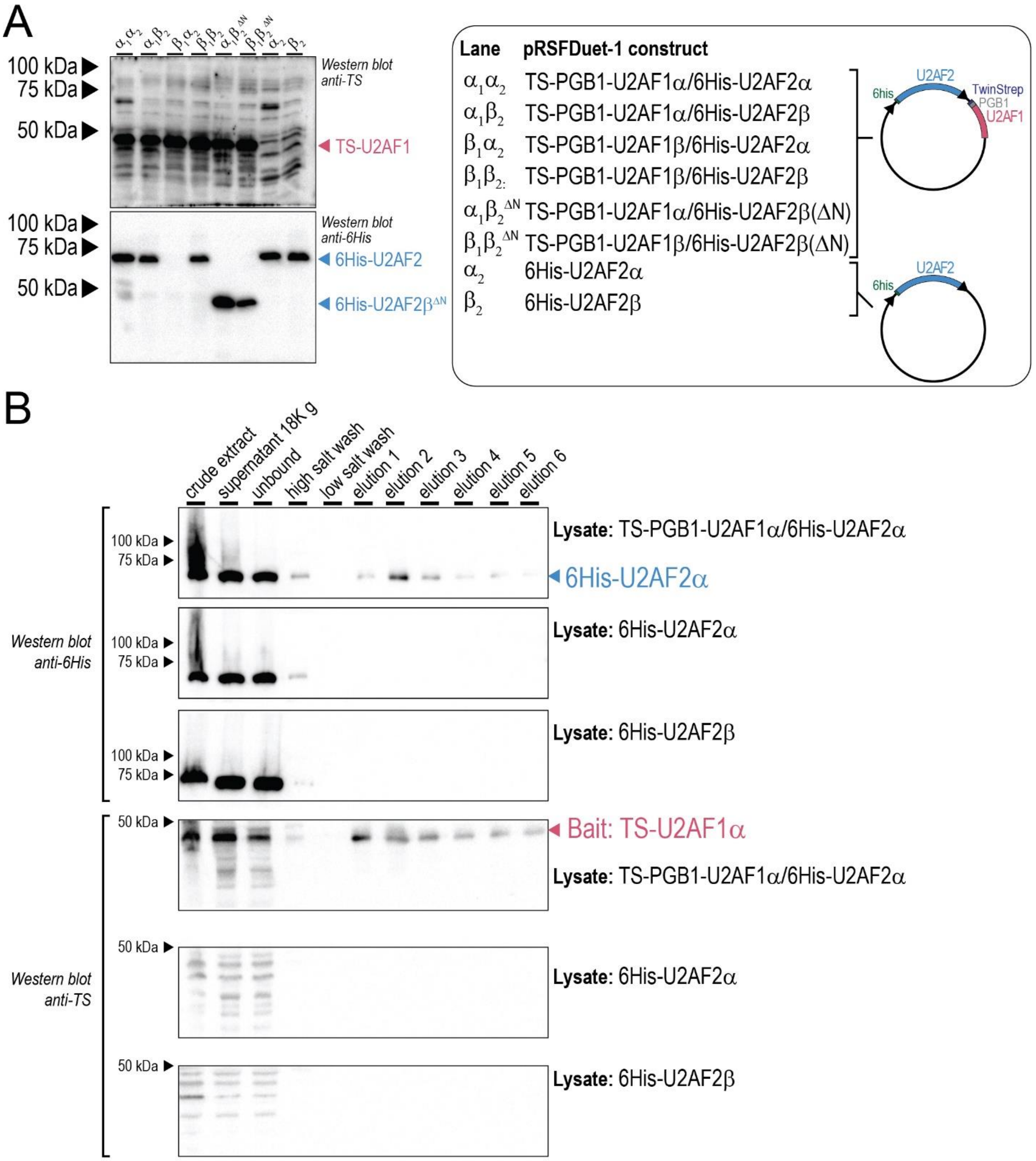
A
α1α2 α1β2 β1α2 β1β2 α1β2ΔN β1β2ΔN α2 β2
100 kDa
75 kDa
50 kDa
Western blot anti-TS
TS-U2AF1
Western blot anti-6His
6His-U2AF2
6His-U2AF2βΔN
Lane pRSFDuet-1 construct
α1α2 TS-PGB1-U2AF1α/6His-U2AF2α
α1β2 TS-PGB1-U2AF1α/6His-U2AF2β
β1α2 TS-PGB1-U2AF1β/6His-U2AF2α
β1β2: TS-PGB1-U2AF1β/6His-U2AF2β
α1β2ΔN TS-PGB1-U2AF1α/6His-U2AF2β(ΔN)
β1β2ΔN TS-PGB1-U2AF1β/6His-U2AF2β(ΔN)
α2 6His-U2AF2α
β2 6His-U2AF2β
6his
U2AF2
TwinStrep
PGB1
U2AF1
B
crude extract
supernatant 18K g
unbound
high salt wash
low salt wash
elution 1
elution 2
elution 3
elution 4
elution 5
elution 6
Lysate: TS-PGB1-U2AF1α/6His-U2AF2α
6His-U2AF2α
Lysate: 6His-U2AF2α
Lysate: 6His-U2AF2β
Western blot anti-6His
Bait: TS-U2AF1α
Lysate: TS-PGB1-U2AF1α/6His-U2AF2α
Lysate: 6His-U2AF2α
Lysate: 6His-U2AF2β
Western blot anti-TS

<u>Fig. S8:</u> Co-purification of *F. borealis* U2AF subunits expressed in *E. coli*. **A)** Gel images show the detection of U2AF proteins expression in crude bacterial extracts with western blots against the fusion tag. **B)** U2AF proteins eluted after binding to a strep-tactin affinity resin. Gel images show proteins detected with western blots against fusion tags, in samples corresponding to either bacterial lysate (lane 1), supernatant of the centrifuged lysate (lane 2), unbound fraction after passing the lysate on strep-tactin (lane 3), material washed from strep-tactin (lanes 4 and 5), proteins eluted from strep-tactin (lanes 6 to 11). We tested lysates from bacteria expressing both U2AF1α and U2AF2α, or only U2AF2α or U2AF2β.

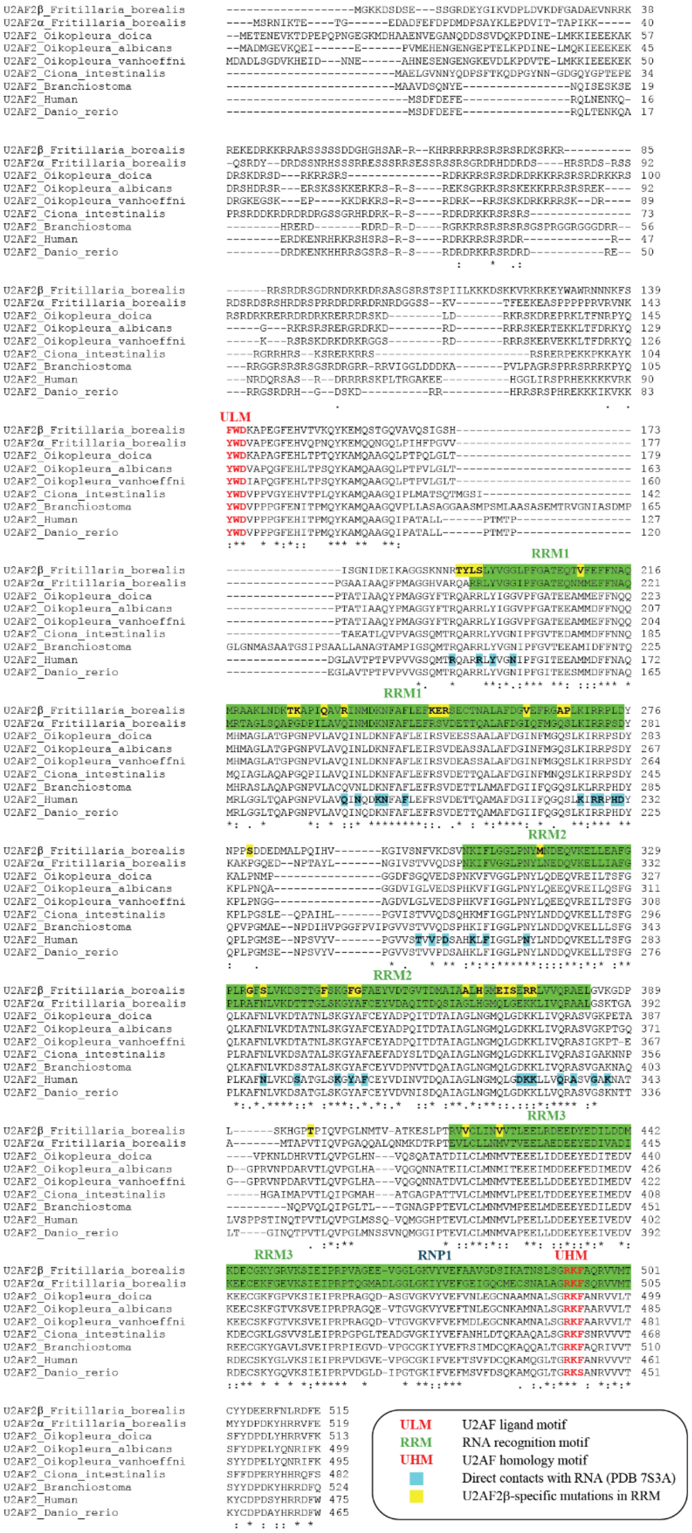

Fig. S9: Multiple sequence alignment of *F. borealis* U2AF2 paralog~~ue~~s with deuterostome U2AF2.

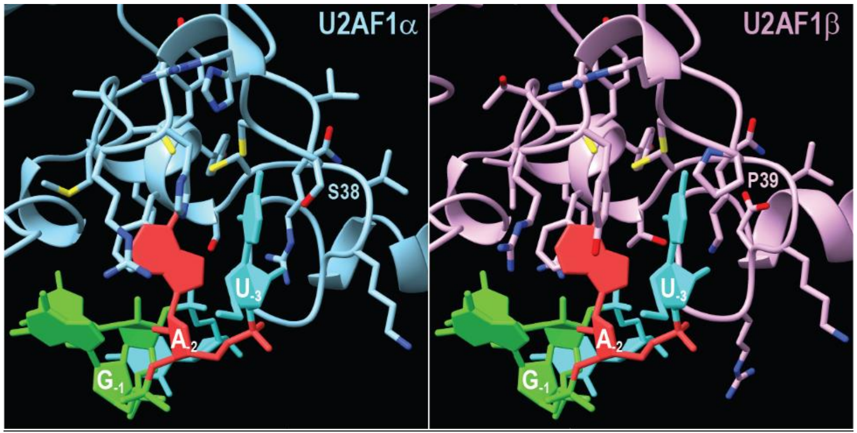

<u>Fig. S10:</u> Model of *F. borealis* U2AF1 paralogues bound to UAGGU, showing how amino acid change in the first zinc finger could impact the recognition of 3'ss. The model was established with SWISS-MODEL[34], using the crystal structure of yeast U2AF bound to UAGGU as a template[35].

2nd/3rd base — U2AF1β vs U2AF1Od

1st base	AA	AC	AG	AU
A	1,2	1,4	1,4	0,5
C	2,5	1,5	0,7	1,1
G	0,4	1,1	1	0,6
U	0,7	1,1	0,7	0,6

2nd/3rd base — U2AF1Od vs U2AF1β

1st base	AA	AC	AG	AU
A	0,8	0,9	0,3	1,5
C	1,7	0,9	1,1	1,2
G	0,6	0,9	1,3	1
U	1,1	1,1	1,8	1,5

2nd base — U2AF1β vs U2AF1Od

1st base	A	C	G	U
A	1,1	1,3	0,9	0,7
C	1,3	1,7	1,3	1,3
G	0,9	1,5	0,4	0,9
U	0,8	1,2	0,9	0,7

2nd base — U2AF1Od vs U2AF1β

1st base	A	C	G	U
A	0,9	0,9	1,1	1,4
C	1	1,3	0,8	1,2
G	1,1	0,8	0,9	1,1
U	1,3	1,3	1,1	1,2

Fig. S11: Results obtained with *F. borealis* U2AF1β and *O. dioica* U2AF1 during RNAcompete experiments. High affinity oligos were identified by comparing RNA enriched in one sample but depleted in the other sample. The tables show the abundance of 3- and 2-mer motifs in the sequence of high affinity oligos. Values correspond to the average frequency in collected oligos relative to the average frequency measured in the initial library.

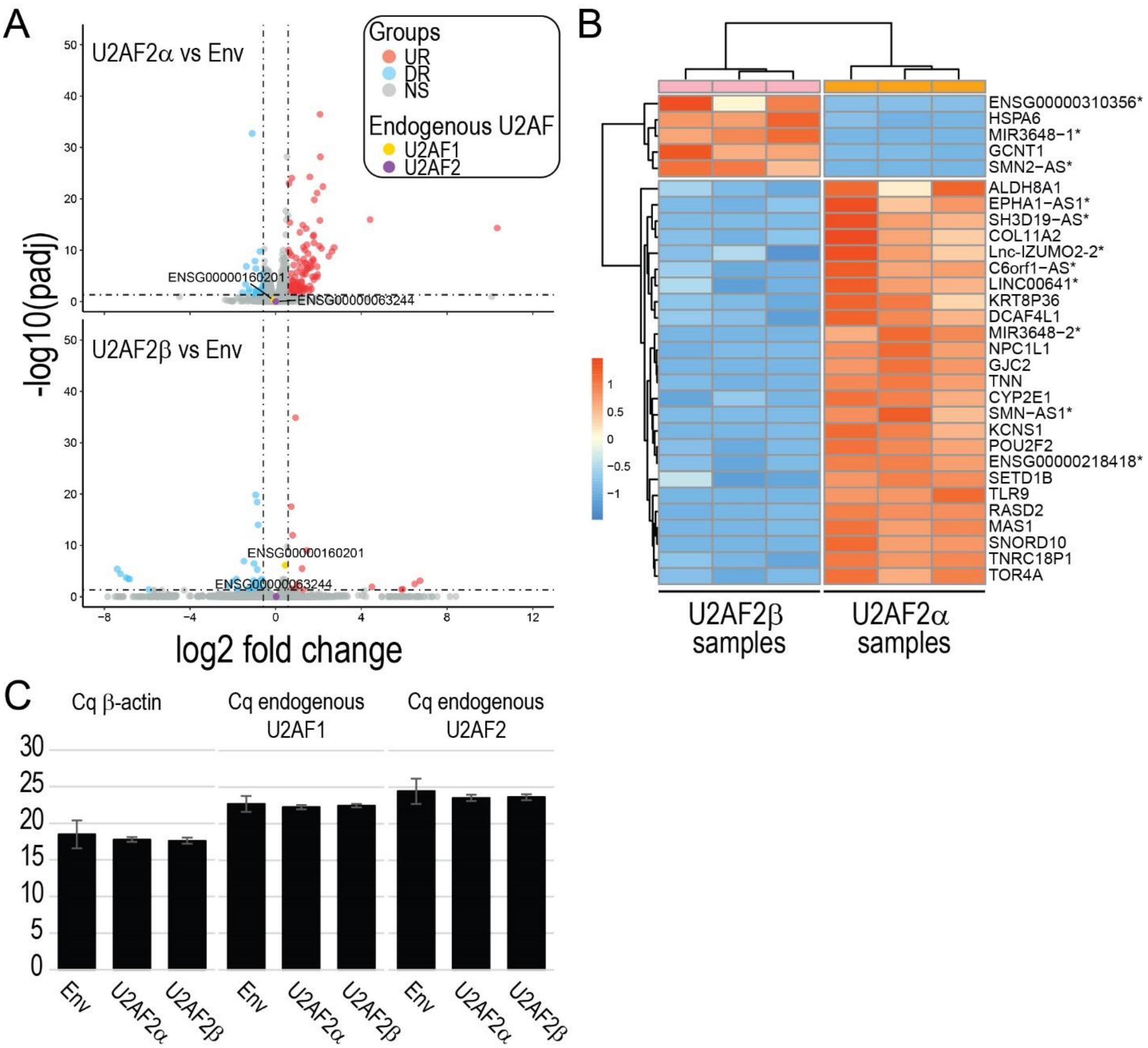

Fig. S12: Transcriptome analysis of HEK293T cells expressing *F. borealis* U2AF2. **A)** Volcano plots showing DEGs found by comparing the transcriptome of cells transfected with either U2AF2 paralogs or control cells expressing an Env protein. UR, upregulated genes; DR, down-regulated genes; NS, non-significant change. **B)** Top 30 DEGs between cells transfected with U2AF2α and cells transfected with U2AF2β. Stars show non-protein-coding genes. **C)** Quantification Cycle (Cq) measured in two-step RT-qPCR experiments targeting either a reference (β-actin) or endogenous U2AF genes, in RNA extracted from cells transfected with *F. borealis* U2AF2 or the Env control.

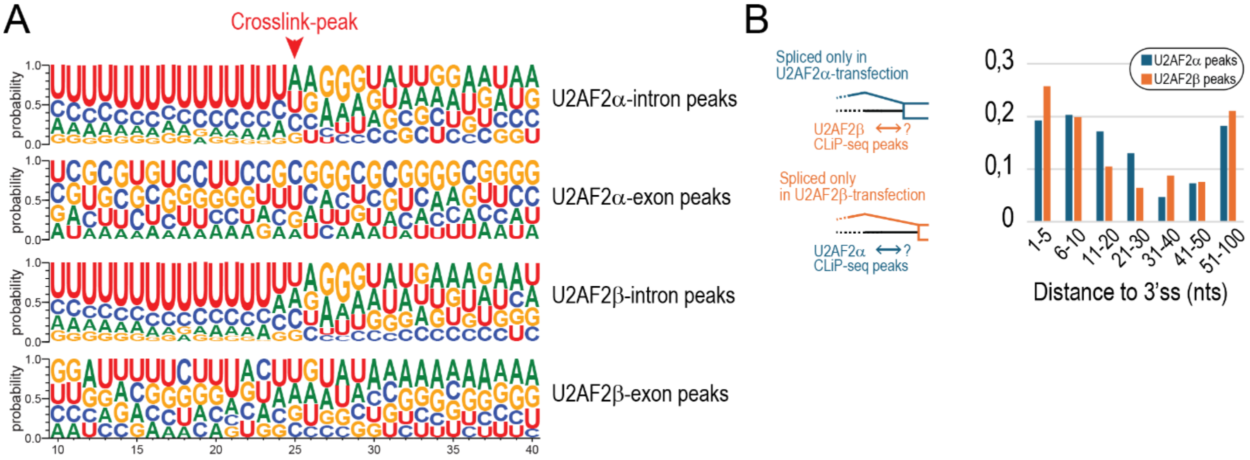

<u>Fig. S13:</u> Results of CLiP-seq analysis. **A)** Sequence logos of sequences surrounding the binding sites of *F. borealis* U2AF2. **B)** Control showing the distribution of U2AF2 crosslink peaks relative to non-specific 3'ss.

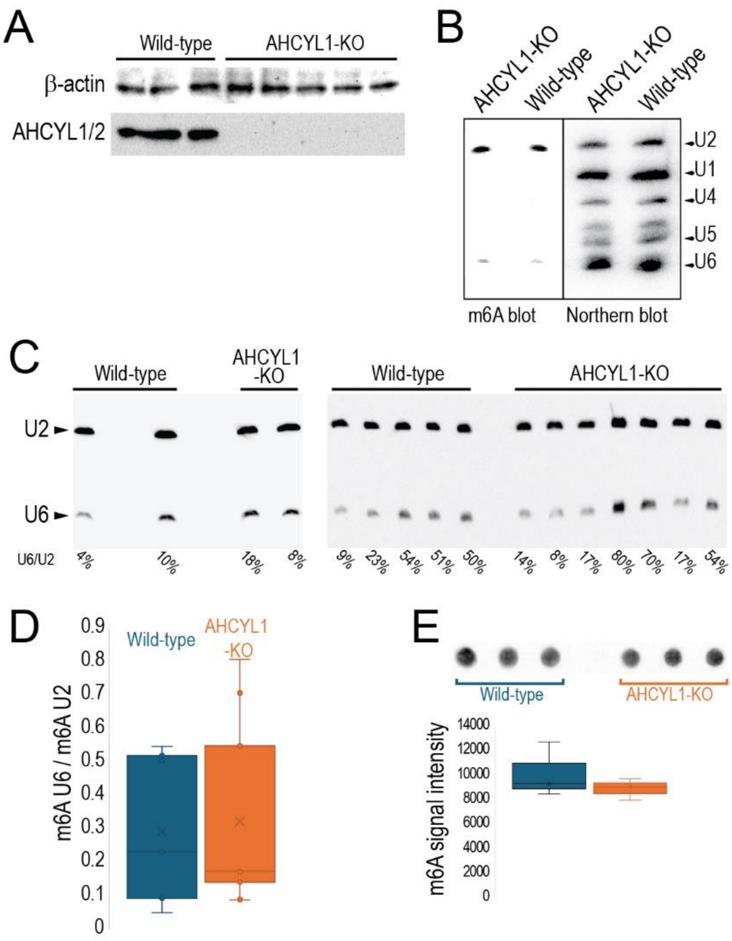

<u>Fig. S14:</u> Evaluating the impact of AHCYL1 on m6A levels. **A)** Western blot showing loss of AHCYL1 expression in HEK293T cells engineered with a CRISPR-Cas9 knock-out. **B)** m6A immunoblot and snRNA Northern blot of AHCYL1-KO cells, compared to wild-type cells. **C)** Immunoblots showing m6A levels in short RNA extracted either with the miRvana procedure or the Trizol reagent (gel on the left and right side, respectively), from either wild-type or AHCYL1-KO cells. For the same cell type, different gel lanes correspond to independent RNA extractions. Percentages below gel lanes correspond to the intensity of the U6 snRNA signal relative to the U2 snRNA signal. **D)** Plot of relative m6A signal intensities measured on U6 and U2 snRNA in wild-type and AHCYL1-KO cells. **E)** Dot blot showing m6A levels measured on poly-adenylated RNA extracted from either wild-type or AHCYL1-KO cells. For the same cell type, different dots correspond to independent RNA extractions.

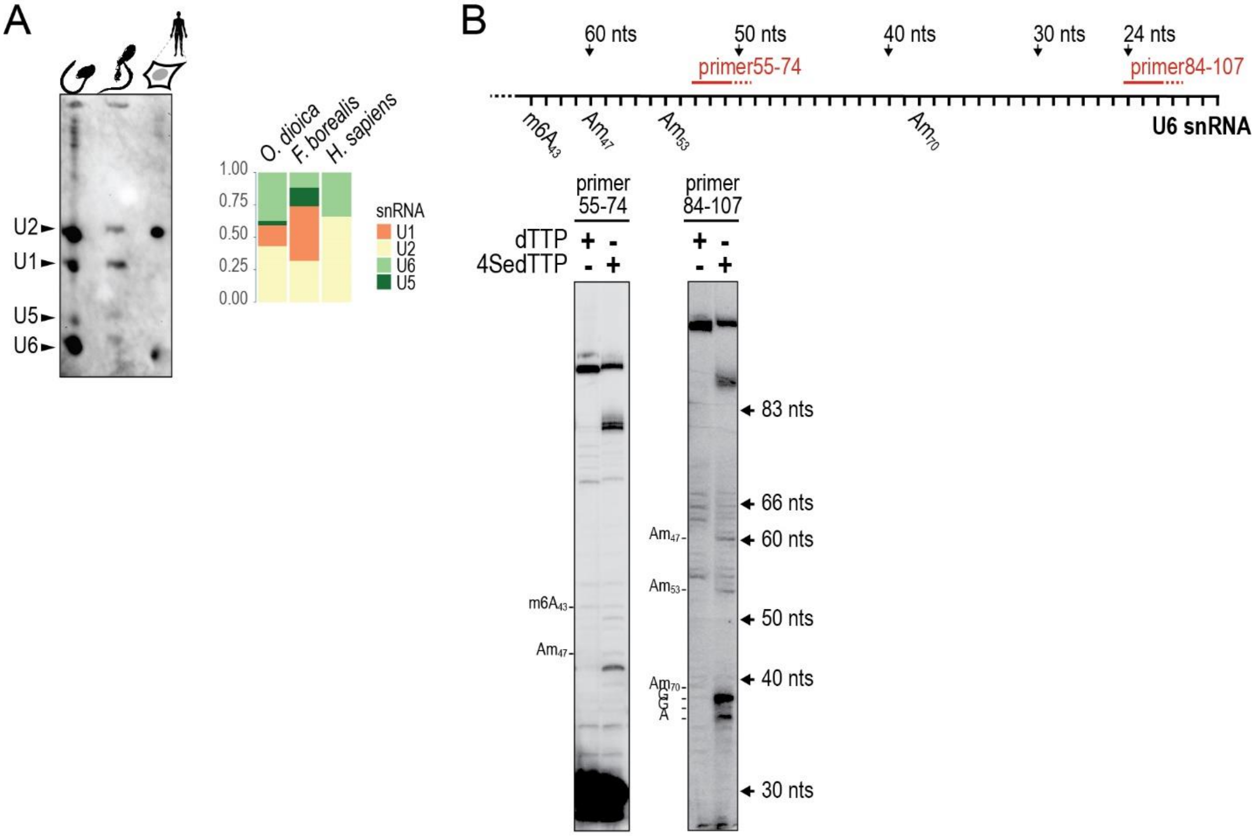

Fig. S15: Detection of modified adenines in snRNA. **A)** Antibody-based detection of m6A on snRNA extracted from *O. dioica*, *F. borealis* and HEK293T cells. The graph shows the distribution of m6A signal between snRNA. **B)** Detection of modified adenosines in human U6 snRNA using a 4SedTTP-sensitive primer extension assay. The schematic represents positions 43-107 of human snRNA with modified adenines [36]. We used two different primers for the RT, annealing either to position 55-74 or 84-107. The size ladder is shown only for the primer annealing to 84-107.

Tables

Table S1: Genome assemblies of larvacean Fritillaridae.

Species	*Fritillaria borealis*	*Fritillaria borealis*	*Fritillaria haplostoma*	*Appendicularia sicula*
Version	2018	2024	2024	2024
Material	Single animal	Pooled animals	Pooled animals	Single animal
Sequencing	Illumina	Illumina + ONT	Illumina	Illumina + ONT
Size (Mb)	89	79	109	65
Contigs	32542	4083	54335	13000
N50 (bp)	10252	39913	2150	6900

Table S2: Base pairing between intron termini of *F. borealis* and their isodiscrepancy indexes

Termini	A-U	A-C	G-G	G-U	A-A	U-U	A-G	G-C	C-U	C-C	U-G	U-A	C-A	U-C	G-A	C-G
tWW	3.71	**2.47**	**0.00**	**3.14**	**1.09**	**3.41**	*n.o.*	6.49	5.40	4.00	**3.24**	3.77	**2.58**	5.42	*n.o.*	6.54
cWH	**3.42**	*n.o.*	**0.00**	*n.o.*	*n.o.*	**2.58**	**2.85**	*n.o.*	3.67	**2.76**	7.25	5.64	*n.o.*	*n.o.*	**2.22**	5.41
Frequency	0.23	0.22	0.10	0.07	0.06	0.05	0.04	0.03	0.03	0.03	0.03	0.03	0.02	0.02	0.02	0.02

Table S3: Oligonucleotides used in the study

Name	Sequence	Experiment
**FbU2AF35a Start**	ATGGACGGATCCGGCGCCGAG	cloning of U2AF1a ORF from cDNA
**FbU2AF35a Stop**	TCAATGCTCTGGATAATACCCTCGTTG	cloning of U2AF1a ORF from cDNA
**FbU2AF35b Start**	ATGCACAACGGTTCGGGCGCTCAC	cloning of U2AF1b ORF from cDNA
**FbU2AF35b Stop**	TTATCGGGGCCTTTGACTGGCTG	cloning of U2AF1b ORF from cDNA
**FbU2AF65a Start**	CATCCCTTCACTTCAATATGTCCAG	cloning of U2AF2a ORF from cDNA
**FbU2AF65a Stop**	CTACTCAAAAACACGCCTATGATATTTG	cloning of U2AF2a ORF from cDNA
**FbU2AF65b Start**	CTTACAGAAATGGGTAAAAAAGACTCAGAC	cloning of U2AF2b ORF from cDNA
**FbU2AF65b Stop**	GTCACTATCATGTCAGATTTAAGTTTCTC	cloning of U2AF2b ORF from cDNA
**INFUpCDN Afwd**	AAGGGTCAAGACAATTCTGCAGATATCC	seamless cloning into pCDNA3.1
**INFUpCDN Arev**	CATGGTGAAGGGTACTGGATCCGAG	seamless cloning into pCDNA3.1
**INFUpSFfw d**	ATGAATTCGAACTCCTCCTGCACGGTAC	seamless cloning into pSF-CMV
**INFUpSFrev**	GCGGCCGCTGGCGGGTGTGTCGAGTG	seamless cloning into pSF-CMV
**pCDNAU2A F65afwd**	GTACCCTTCACCATGTCCAGAAATATAAAAACAGAAACC	subcloning of U2AF2a ORF in pCDNA3.1
**pCDNAU2A F65arev**	AATTGTCTTGACCCTTCTCAAAAACACGCCTATGATATTTGTC	subcloning of U2AF2a ORF in pCDNA3.1
**pCDNAU2A F65bfwd**	GTACCCTTCACCATGGGTAAAAAAGACTCAGACTCC	subcloning of U2AF2b ORF in pCDNA3.1
**pCDNAU2A F65brev**	AATTGTCTTGACCCTTTTCAAAATCTCTCAGATTGAACCTCTC	subcloning of U2AF2b ORF in pCDNA3.1
**pSFU2AF35 afwd**	CCCGCCAGCGGCCGCATGGACGGATCCGGCGCCGAG	subcloning of U2AF1a ORF in pSF-CMV-GST(NH_2)
**pSFU2AF35 arev**	GGAGTTCGAATTCATATGCTCTGGATAATACCCTCGTTG	subcloning of U2AF1a ORF in pSF-CMV-GST(NH_2)
**pSFU2AF35 bfwd**	CCCGCCAGCGGCCGCATGCACAACGGTTCGGGCGCTC	subcloning of U2AF1b ORF in pSF-CMV-GST(NH_2)
**pSFU2AF35 brev**	GGAGTTCGAATTCATTCGGGGCCTTTGACTGGC	subcloning of U2AF1b ORF in pSF-CMV-GST(NH_2)
**IF_GST_U2 AF35a3**	GAGACAACTTCTAGATCAATGCTCTGGATAATACCCTCGTTG	subcloning of U2AF1a ORF in pGEX-6P-1
**IF_GST_U2 AF35a5**	CCAGGGCATGAATTCGGACGGATCCGGCGCCGAGTAC	subcloning of U2AF1a ORF in pGEX-6P-1
**IF_GST_U2 AF35b3**	GAGACAACTTCTAGATTATCGGGGCCTTTGACTGGC	subcloning of U2AF1b ORF in pGEX-6P-1
**IF_GST_U2 AF35b5**	AACTCCTCCTGCACGGTACCGCACAACGGTTCGGGCGCTC	subcloning of U2AF1b ORF in pGEX-6P-1
**ODIOU1.AS 13-33**	TTGATCGAGGTTACCCTCCG	mapping m6A by primer extension - Oikopleura dioica U1
**HSAPU1.AS 13-33**	GTGATCATGGTATCTCCCCT	mapping m6A by primer extension - Human U1
**FBORU1-1.AS13-33**	TTGATCACACTGTACCGCCC	mapping m6A by primer extension - Fritillaria borealis U1a
**FBORU1-2.AS13-33**	TTGATCACTCTGTACCGCCC	mapping m6A by primer extension - Fritillaria borealis U1b
**HSAPU6.AS 55-74**	ATCCTTGCACAGGGGCCATG	mapping m6A by primer extension - Human U6
**HSAPU6.AS 84-107**	AAAAATATGGAACGCTTCACGAAT	mapping m6A by primer extension - Human U6
**FBORU6.AS 53-72**	ATCCTTGCGCAGGGGCCATG	mapping m6A by primer extension – Fritillaria borealis U6

**U2.NB3.AS_ FBOR**	GTCAAACTTTGAAGAACAGATACTACACTTGATCTTAGCCACAAAGGCCGGAAGC	Northern blot U2 snRNA probe - Fritillaria borealis
**U1.NB2.AS_ FBOR**	GCTATCCCCCACTCTCAGAAATTTTACGGGACGCTGAGCCTGTTTGGCCCAGTG	Northern blot U1 snRNA probe - Fritillaria borealis
**U4.NB2.AS_ FBOR**	GAGAAAGTCGGGTATTTCACTGTCAAACCGCGGGGTATTGGGAATAGTTTTC	Northern blot U4 snRNA probe - Fritillaria borealis
**U5.NB2.AS_ FBOR**	GAGATATATATCTCATAAGCGTTCTCTCAACACGGTGTTGGACAGTAAAAGGCGGTTC	Northern blot U5 snRNA probe - Fritillaria borealis
**U6.NB2.AS_ FBOR**	GAACCACTTCTCGATTTGTGCGTGTCATCCTTGCGCAGGGGCCATGCTAATCTTC	Northern blot U6 snRNA probe - Fritillaria borealis
**U1.NB2.AS_ ODIO**	GACACTCCACCGATTTGGCAAAGAGACACAGGTCAGCCGCGCCGGTGTGCAATGGTAC	Northern blot U1 snRNA probe - Oikopleura dioica
**U2.NB2.AS_ ODIO**	GTACTGCAATAGCGAGTCAACCCGTGGGAAGAGCGGAGCAAGCCCCTAACTGCATC	Northern blot U2 snRNA probe - Oikopleura dioica
**U4.NB2.AS_ ODIO**	GTTTTCAATTAGCAATAATCGCAACTCGGATAAACCTCATCGTTTACGATACTGCCAC	Northern blot U4 snRNA probe - Oikopleura dioica
**U5.NB2.AS_ ODIO**	GAGCTTCAGAAAAATCAAATACTCATAAGAGTGTCCCTCTCCACGGAATTCTTTAG	Northern blot U5 snRNA probe - Oikopleura dioica
**U6.NB2.AS_ ODIO**	GAACGCTTCACGATTTTGCGTGTCATCCTTGCGCAGGGGCCATGCTAATCTTCTCTG	Northern blot U6 snRNA probe - Oikopleura dioica
**U1.NB2.AS_ HSAP**	GCAGTCGAGTTTCCCACATTTGGGGAAATCGCAGGGGTCAGCACATCCGGAGTGC	Northern blot U1 snRNA probe - Human
**U2.NB2.AS_ HSAP**	GTTGATGCATGGAGTGGACGGAGCAAGCTCCTAGTCCATGTCCCTGCTCCAAAAATCC	Northern blot U2 snRNA probe - Human
**U4.NB2.AS_ HSAP**	GTTTTCAATTAGCAATAATCGCGCCTCGGATAAACCTCATTGGCTACGATACTGCCAC	Northern blot U4 snRNA probe - Human
**U5.NB2.AS_ HSAP**	GTTTCAGACTCACACTCGTTTCTCTCCACGGAAATCTTTAGTAAAAGGCGAAAG	Northern blot U5 snRNA probe - Human
**U6.NB2.AS_ HSAP**	GAACGCTTCACGAATTTGCGTGTCATCCTTGCACAGGGGCCATGCTAATCTTCTC	Northern blot U6 snRNA probe - Human
**U1.NB2.AS_ CINT**	GAAGCCTCACCTTGGACCCACTGCCTGCCTGATCGCAGTGAAGGCCCTCGTCAGG	Northern blot U1 snRNA probe - Ciona intestinalis
**U2.NB2.AS_ CINT**	GACAACCCGTGAGCAGGGTGGAGCAAGCTCCAGCACCATGCCCTATGTTCAAAAATC	Northern blot U2 snRNA probe - Ciona intestinalis
**U4.NB2.AS_ CINT**	GAATAGTTTTCAACTAGCAATAATCGCGCCTCGGATGAACCTCATCGGCTACGATAC	Northern blot U4 snRNA probe - Ciona intestinalis
**U5.NB2.AS_ CINT**	GTCAGGTGTGGAAAAATAATCAAAGACTCATTGAAATGCTCCTCTCCACGGAATTC	Northern blot U5 snRNA probe - Ciona intestinalis
**U6.NB2.AS_ CINT**	GAACGCTTCACGATTTTGCGTGTCATCCTTGCGCAGGGGCCATGCTAATCTTCTCTG	Northern blot U6 snRNA probe - Ciona intestinalis
**DELNt.U2A F2b.F**	TGGGCGTGGAGAAACAATAATAAATTCAGTTTTTG	Engineering N-terminal deletion mutant of U2AF2b
**DELNt.U2A F2b.R**	GTTTCTCCACGCCCACGGATCCTGGCTGTGGTGATGATGGTGATG	Engineering N-terminal deletion mutant of U2AF2b
**Fb3ssPoolS aplv2**	AGTCAGTCTGTATCAGCTAGCTCTTCA	amplification of intron library for RNA compete
**Fb3ssPoolT 7v2**	AGACCAGCTCTAATACGACTCACTATAGG	amplification of intron library for RNA compete
**Hs.ALG13.e 27AS**	TGCAAAAGACACCAATTCAATACAGTGCA	amplification of human ALG13 cDNA
**Hs.ALG13.e 4S**	TGGGCTGCTTTCCGGATACCTGCA	amplification of human ALG13 cDNA
**Hs.EIF1B.e1 S**	CACTCCAGCCTAATCCCAACCCCAGG	amplification of human eIF1b cDNA
**Hs.EIF1B.e4 AS**	AGCTGCAACGTACATGGTTTAATACAACA	amplification of human eIF1b cDNA
**Hs.FMR1.e1 6AS**	TGTCACATCACACACATTTCAGGGTCC	amplification of human FMRI cDNA
**Hs.FMR1.e1 S**	GAGCTGGTGGTGGAAGTGCGGG	amplification of human FMRI cDNA
**Hs.HSP90B 1.e18AS**	TGCAGGGGAGAAGGGGATTAGGGG	amplification of human HSP90b1 cDNA
**Hs.HSP90B 1.e1S**	GACTTGAGACTCACCGGCCGCAC	amplification of human HSP90b1 cDNA
**Hs.TARDBP .e1S**	TGCTCTCCACGGTTACAGCCCAGT	amplification of human TARDBP cDNA
**Hs.TARDBP .e6AS**	GGGATTAAACCATGCCGTTGACAAGT	amplification of human TARDBP cDNA

**pRSFVECTwinStrepF**	CCGCAGTTCGAAAAACTGGAAGTTCTGTTCCAGGGGC	Insertion of the Twin-strep tag
**pRSFVECTwinStrepR**	GCTCCAAGCGCTGGCCATATGTATATCTCCTTCTTATAC	Insertion of the Twin-strep tag
**TwinStrepF**	GCCAGCGCTTGGAGCCACCCGCAGTTCGAGAAAGGTGGAGGTTCCGGAGGTGGATCG	Insertion of the Twin-strep tag
**TwinStrepR**	TTTTTCGAACTGCGGGTGGCTCCACGCCGAACCTCCCGATCCACCTCCGGAACCTCCACC	Insertion of the Twin-strep tag
**PGB1FWD**	CCGCAGTTCGAAAAAACCTATAAACTGATTCTGAACGGCAAAACCCTGAAAGGCGAAACCACCAC CGAAGCGGTGGATGCGGCGACCGCGGAAAAAGTGTTTAAACAGTATGCGAACGAT	Insertion of the PGB1 solubilization domain
**PGB1FWD-ATG**	ATGACCTATAAACTGATTCTGAACGGCAAAACCCTGAAAGGCGAAACCACCACCGAAGCGGTGGA TGCGGCGACCGCGGAAAAAGTGTTTAAACAGTATGCGAACGAT	Insertion of the PGB1 solubilization domain
**PGB1REV35a**	CGGCGCCGGATCCGTCGCTCTGAAAATACAGGTTTTCTTCGGTCACGGTAAAGGTTTTGGTCGCA TCATCATAGGTCCATTCGCCATCCACGCCGTTATCGTTCGCATACTGTTTAAACA	Insertion of the PGB1 solubilization domain
**PGB1REV35b**	CGCCCGAACCGTTGTGGCTCTGAAAATACAGGTTTTCTTCGGTCACGGTAAAGGTTTTGGTCGCA TCATCATAGGTCCATTCGCCATCCACGCCGTTATCGTTCGCATACTGTTTAAACA	Insertion of the PGB1 solubilization domain
**pRSFINSFb35aR**	GCAGCAGCCTAGGTTAATTAATGCTCTGGATAATACCCTCG	subcloning of U2AF1a ORF in pRSF-DUET
**pRSFINSFb35bR**	GCAGCAGCCTAGGTTAATTATCGGGGCCTTTGACTGGC	subcloning of U2AF1b ORF in pRSF-DUET
**pRSFINSFb65aF**	ATCACCACAGCCAGGATCCGTCCAGAAATATAAAAACAGAAACCG	subcloning of U2AF2a ORF in pRSF-DUET
**pRSFINSFb65aR**	GACTTAAGCATTACTCAAAAACACGCCTATG	subcloning of U2AF2a ORF in pRSF-DUET
**pRSFINSFb65bF**	ATCACCACAGCCAGGATCCGGGTAAAAAAGACTCAGACTCCG	subcloning of U2AF2b ORF in pRSF-DUET
**pRSFINSFb65bR**	GACTTAAGCATTATTCAAAATCTCTCAGATTGAAC	subcloning of U2AF2b ORF in pRSF-DUET
**pRSFVECF**	TAATTAACCTAGGCTGCTGCCACC	subcloning in pRSF-DUET
**pRSFVECLINKFb65aF**	TTTTGAGTAATGCTTAAGTCGAACAGAAAG	subcloning of U2AF2a ORF in pRSF-DUET
**pRSFVECLINKFb65bF**	TTTTGAATAATGCTTAAGTCGAACAGAAAG	subcloning of U2AF2b ORF in pRSF-DUET
**pRSFVECLINKR**	TAGGGGACATATGTATATCTCCTTCTTATACTTAA	subcloning in pRSF-DUET
**pRSFVECR**	CGGATCCTGGCTGTGGTGATGATGG	subcloning in pRSF-DUET
**QPCRf_U2AF1_HSAP**	GAACGTCTGTGACAACCTGGGA	RT-qPCR primer, human U2AF1
**QPCRr_U2AF1_HSAP**	AGGCTTCTCTGAAGTCCGTCAC	RT-qPCR primer, human U2AF1
**QPCRf_U2AF2_HSAP**	TACGGGCTTGTCAAGTCCATCG	RT-qPCR primer, human U2AF2
**QPCRf_U2AF2_HSAP**	CTGGCAGTCAAACACAGAGGTG	RT-qPCR primer, human U2AF2
**QPCRf_BACT_HSAP**	CACCATTGGCAATGAGCGGTTC	RT-qPCR primer, human β-actin
**QPCRr_BACT_HSAP**	AGGTCTTTGCGGATGTCCACGT	RT-qPCR primer, human β-actin

Supplemental data included in compressed folder:

- **Dataset S1 (separate file: gels.tar.gz).** Uncropped gel pictures.
- **Dataset S2 (separate file: Crux_output.tar.gz)**. Protein database used in the Crux pipeline, and output from percolator and spectral-counts steps.

Supplemental spreadsheets:

- **SI_coIPMS.xlsx**. Mass spectrometry results on proteins extracted from co-IP experiments
- **SI_homology.xlsx**. BLAST top hits of gene homologs mentioned in the study
- **SI_TALONcount.xlsx.** Summary of TALON output, including read count on detected isoforms, total read number per sample, isoforms and introns retained for analysis, and gene models used for amplicon cDNA alignment.
- **SI_U2AF1count.xlsx.** Summary of RNAcompete sequence processing, including the composition of RNA oligonucleotide pool, the read count per RNA oligonucleotide in protein-bound material, and normalized counts for U2AF1α and U2AF1β experiments.

Supplemental references

1. Henriet, S., Aasjord, A., and Chourrout, D. (2022). Laboratory study of Fritillaria lifecycle reveals key morphogenetic events leading to genus-specific anatomy. Front Zool *19*, 26.
2. Henriet, S., Colom Sanmarti, B., Sumic, S., and Chourrout, D. (2019). Evolution of the U2 Spliceosome for Processing Numerous and Highly Diverse Non-canonical Introns in the Chordate Fritillaria borealis. Curr Biol *29*, 3193-3199.e3194.
3. Henriet, S., Sumic, S., Doufoundou-Guilengui, C., Jensen, M.F., Grandmougin, C., Fal, K., Thompson, E., Volff, J.N., and Chourrout, D. (2015). Embryonic expression of endogenous retroviral RNAs in somatic tissues adjacent to the Oikopleura germline. Nucleic Acids Res *43*, 3701-3711.
4. Olthof, A.M., Hyatt, K.C., and Kanadia, R.N. (2019). Minor intron splicing revisited: identification of new minor intron-containing genes and tissue-dependent retention and alternative splicing of minor introns. BMC Genomics *20*, 686.
5. UniProt, C. (2025). UniProt: the Universal Protein Knowledgebase in 2025. Nucleic Acids Res *53*, D609-D617.
6. Dyer, S.C., Austine-Orimoloye, O., Azov, A.G., Barba, M., Barnes, I., Barrera-Enriquez, V.P., Becker, A., Bennett, R., Beracochea, M., Berry, A., et al. (2025). Ensembl 2025. Nucleic Acids Res *53*, D948-D957.
7. Dardaillon, J., Dauga, D., Simion, P., Faure, E., Onuma, T.A., DeBiasse, M.B., Louis, A., Nitta, K.R., Naville, M., Besnardeau, L., et al. (2020). ANISEED 2019: 4D exploration of genetic data for an extended range of tunicates. Nucleic Acids Res *48*, D668-D675.

8. Bailey, T.L. (2021). STREME: accurate and versatile sequence motif discovery. Bioinformatics *37*, 2834-2840.
9. Crooks, G.E., Hon, G., Chandonia, J.M., and Brenner, S.E. (2004). WebLogo: a sequence logo generator. Genome Res *14*, 1188-1190.
10. Clark, F., and Thanaraj, T.A. (2002). Categorization and characterization of transcript-confirmed constitutively and alternatively spliced introns and exons from human. Hum Mol Genet *11*, 451-464.
11. Patro, R., Duggal, G., Love, M.I., Irizarry, R.A., and Kingsford, C. (2017). Salmon provides fast and bias-aware quantification of transcript expression. Nat Methods *14*, 417-419.
12. Love, M.I., Huber, W., and Anders, S. (2014). Moderated estimation of fold change and dispersion for RNA-seq data with DESeq2. Genome Biol *15*, 550.
13. Soneson, C., Love, M.I., and Robinson, M.D. (2015). Differential analyses for RNA-seq: transcript-level estimates improve gene-level inferences. F1000Res *4*, 1521.
14. Wang, Y., Xie, Z., Kutschera, E., Adams, J.I., Kadash-Edmondson, K.E., and Xing, Y. (2024). rMATS-turbo: an efficient and flexible computational tool for alternative splicing analysis of large-scale RNA-seq data. Nat Protoc *19*, 1083-1104.
15. Krakau, S., Richard, H., and Marsico, A. (2017). PureCLIP: capturing target-specific protein-RNA interaction footprints from single-nucleotide CLIP-seq data. Genome Biol *18*, 240.
16. Li, D., Liu, C.M., Luo, R., Sadakane, K., and Lam, T.W. (2015). MEGAHIT: an ultra-fast single-node solution for large and complex metagenomics assembly via succinct de Bruijn graph. Bioinformatics *31*, 1674-1676.
17. Pardo-Palacios, F.J., Arzalluz-Luque, A., Kondratova, L., Salguero, P., Mestre-Tomas, J., Amorin, R., Estevan-Morio, E., Liu, T., Nanni, A., McIntyre, L., et al. (2024). SQANTI3: curation of long-read transcriptomes for accurate identification of known and novel isoforms. Nat Methods *21*, 793-797.
18. Artemyeva-Isman, O.V., and Porter, A.C.G. (2021). U5 snRNA Interactions With Exons Ensure Splicing Precision. Front Genet *12*, 676971.
19. Olthof, A.M., Schwoerer, C.F., Girardini, K.N., Weber, A.L., Doggett, K., Mieruszynski, S., Heath, J.K., Moore, T.E., Biran, J., and Kanadia, R.N. (2024). Taxonomy of introns and the evolution of minor introns. Nucleic Acids Res *52*, 9247-9266.
20. Lemoine, F., Correia, D., Lefort, V., Doppelt-Azeroual, O., Mareuil, F., Cohen-Boulakia, S., and Gascuel, O. (2019). NGPhylogeny.fr: new generation phylogenetic services for non-specialists. Nucleic Acids Res *47*, W260-W265.
21. Rice, P., Longden, I., and Bleasby, A. (2000). EMBOSS: the European Molecular Biology Open Software Suite. Trends Genet *16*, 276-277.
22. Zhou, P., Lugovskoy, A.A., and Wagner, G. (2001). A solubility-enhancement tag (SET) for NMR studies of poorly behaving proteins. J Biomol NMR *20*, 11-14.
23. Wyman, D., Balderrama-Gutierrez, G., Reese, F., Jiang, S., Rahmanian, S., Forner, S., Matheos, D., Zeng, W., Williams, B., Trout, D., et al. (2020). A technology-agnostic long-read analysis pipeline for transcriptome discovery and quantification. bioRxiv, 672931.

24. Chen, S. (2023). Ultrafast one-pass FASTQ data preprocessing, quality control, and deduplication using fastp. Imeta *2*, e107.
25. Dobin, A., Davis, C.A., Schlesinger, F., Drenkow, J., Zaleski, C., Jha, S., Batut, P., Chaisson, M., and Gingeras, T.R. (2013). STAR: ultrafast universal RNA-seq aligner. Bioinformatics *29*, 15-21.
26. Mudge, J.M., Carbonell-Sala, S., Diekhans, M., Martinez, J.G., Hunt, T., Jungreis, I., Loveland, J.E., Arnan, C., Barnes, I., Bennett, R., et al. (2025). GENCODE 2025: reference gene annotation for human and mouse. Nucleic Acids Res *53*, D966-D975.
27. Buchbender, A., Mutter, H., Sutandy, F.X.R., Kortel, N., Hanel, H., Busch, A., Ebersberger, S., and Konig, J. (2020). Improved library preparation with the new iCLIP2 protocol. Methods *178*, 33-48.
28. Roehr, J.T., Dieterich, C., and Reinert, K. (2017). Flexbar 3.0 - SIMD and multicore parallelization. Bioinformatics *33*, 2941-2942.
29. Smith, T., Heger, A., and Sudbery, I. (2017). UMI-tools: modeling sequencing errors in Unique Molecular Identifiers to improve quantification accuracy. Genome Res *27*, 491-499.
30. McIlwain, S., Tamura, K., Kertesz-Farkas, A., Grant, C.E., Diament, B., Frewen, B., Howbert, J.J., Hoopmann, M.R., Kall, L., Eng, J.K., et al. (2014). Crux: rapid open source protein tandem mass spectrometry analysis. J Proteome Res *13*, 4488-4491.
31. Hong, T., Yuan, Y., Chen, Z., Xi, K., Wang, T., Xie, Y., He, Z., Su, H., Zhou, Y., Tan, Z.J., et al. (2018). Precise Antibody-Independent m6A Identification via 4SedTTP-Involved and FTO-Assisted Strategy at Single-Nucleotide Resolution. J Am Chem Soc *140*, 5886-5889.
32. Abramson, J., Adler, J., Dunger, J., Evans, R., Green, T., Pritzel, A., Ronneberger, O., Willmore, L., Ballard, A.J., Bambrick, J., et al. (2024). Accurate structure prediction of biomolecular interactions with AlphaFold 3. Nature *630*, 493-500.
33. Kielkopf, C.L., Rodionova, N.A., Green, M.R., and Burley, S.K. (2001). A novel peptide recognition mode revealed by the X-ray structure of a core U2AF35/U2AF65 heterodimer. Cell *106*, 595-605.
34. Waterhouse, A., Bertoni, M., Bienert, S., Studer, G., Tauriello, G., Gumienny, R., Heer, F.T., de Beer, T.A.P., Rempfer, C., Bordoli, L., et al. (2018). SWISS-MODEL: homology modelling of protein structures and complexes. Nucleic Acids Res *46*, W296-W303.
35. Yoshida, H., Park, S.Y., Sakashita, G., Nariai, Y., Kuwasako, K., Muto, Y., Urano, T., and Obayashi, E. (2020). Elucidation of the aberrant 3' splice site selection by cancer-associated mutations on the U2AF1. Nat Commun *11*, 4744.
36. Krogh, N., Kongsbak-Wismann, M., Geisler, C., and Nielsen, H. (2017). Substoichiometric ribose methylations in spliceosomal snRNAs. Org Biomol Chem *15*, 8872-8876.
